# Evaluation of droplet digital PCR and next generation sequencing for characterizing DNA reference material for *KRAS* mutation detection

**DOI:** 10.1038/s41598-018-27368-3

**Published:** 2018-11-30

**Authors:** Lianhua Dong, Shangjun Wang, Boqiang Fu, Jing Wang

**Affiliations:** 1 National Institute of Metrology, Beijing, 100013 P. R. China; 2 Nanjing Institute of Measurement and Testing Technology, Nanjing, 210049 P. R. China

## Abstract

*KRAS* gene mutations are predictive markers of non-response to anti-epidermal growth factor receptor. An increasing number of techniques are being developed to detect *KRAS* mutations. To obtain consistent and comparable results, a traceable reference material (RM) is necessary for validation the routinely used method. However, a lack of reference methods is a main impediment for deriving traceability and measurement comparability. In this study, droplet digital PCR (ddPCR) and next generation sequencing (NGS) were evaluated. No cross- reactivity was detected with any of the probe by ddPCR. The measured fraction of *KRAS* mutant allele by ddPCR and NGS agreed with the prepared value by gravimetrical dilution (concordance (*k*) >0.95 and >0.93 for ddPCR and NGS, respectively). The reliable limit of quantification (LOQ) was 0.1% and 1% for ddPCR and NGS, respectively. In conclusion, the validated ddPCR and NGS are suitable to characterize the *KRAS* RM due to the high specificity and accuracy. Verification of the LOD of three commercial kits by using the NIM-KRAS-8 RM showed that the LOD was inconsistent with the claimed LOD of the kits (1%) for some assays. This indicates a traceable RM was important for setting up the criteria regarding the LOD for the commercial kit.

## Introduction

*KRAS* (v-Ki-ras2 Kirsten rat sarcoma viral oncogene homolog) gene mutations are predictive markers of non response to anti-epidermal growth factor receptor (anti-EGFR)^1–3^ and therefore valuable for prognosis and treatment of pancreatic cancer and colorectal cancer^4,5^. *KRAS* mutations located in codon 12 and codon 13 of exon 2^6^ are among the most frequently detected activating mutations in human cancers, being present in 65% to 100% of pancreatic cancer and 30% to 50% of colorectal cancer cases.

*KRAS* mutations have been reported to appear in the early stages of tumorigenesis in pancreatic and colorectal human cancers^7,8^. Detection of *KRAS* mutations in patient blood samples, pancreatic juice and stool DNA facilitate early diagnosis of pancreatic and colorectal cancer^9,10^. Furthermore, *KRAS* mutations are also used as major prognostic biomarkers for therapies that target the EGFR in patients with metastatic colorectal cancer, as cancer bearing *KRAS* mutations are reportedly unresponsive to anti- EGFR monoclonal antibodies such as cetuximab and panitumumab^3,11–14^.

Development of a method for detection of *KRAS* mutations and a detection kit for clinical diagnostic use is particularly attractive. An increasing number of techniques are being developed to detect *KRAS* mutations. Currently, KRAS mutations are routinely detected by Sanger sequencing, next generation sequencing (NGS), ARMS-PCR (amplification-refractory mutation system), mutant-enriched PCR, COLD-PCR and digital PCR (dPCR). These techniques have different levels of LOD and specificity. Direct sequencing has a reported a LOD of approximately 20% mutant alleles^15,16^, whereas NGS is capable of detecting mutant alleles at levels as low as about 2–6%^17,18^. ARMS-PCR has a LOD of around 1%^19^, while mutant-enriched PCR and COLD-PCR have greater sensitivity for detecting *KRAS* mutations, with a limit of detection of about 0.1%^20–22^. Recently, chip-based dPCR and droplet-based dPCR have been reported to have a *KRAS* mutation LOD of 0.05%^23^ and 0.01%^24^. However, the different LOD of the analyses give rise to inconsistencies and incomparability of results of clinical testing. Furthermore, our recently study showed detection kits from different manufacturers give inconsistence result even though they were with the same claimed LOD for *KRAS* mutation detection.

One solution to the problem of incomparability in molecular diagnostic is to use a SI (international system of unit) traceable RM to validate the protocol. However, a lack of reference methods and materials is a main impediment for deriving traceability and measurement comparability. Currently, national metrology institutes (NMIs) are working to establish higher order reference analytical procedures to provide reliable methods for this purpose. The present study was conducted (1) to establish a highly accurate *KRA*S allele frequency measurement; (2) develop a traceable *KRAS* mutant reference material characterized with the established method to validate *KRAS* mutation detection kits.

## Material and Methods

### Cell lines

Cell lines of RPMI-8226 (KRAS G12A), NCI-H157 (KRAS G12R), A549 (KRAS G12S), SW620 (KRAS G12V), HCT-116 (KRAS G13D) and 293 T (wild type) were obtained from the Institute of Basic Medical Sciences Chinese Academy of Medical Sciences (CAMS). The SW1573 and the SUN-C2B cell lines were purchased from the American Type Culture Collection (ATCC). Culture medium for each cell line was determined according to the information provided by ATCC and CAMS. Cell lines were cultured in a humidified atmosphere of 5% CO_2_ at 37 °C. Subcultures were made at a ratio of 1:3 when the cell density reached 80%–90% every 3 or 4 days.

### DNA extraction

Genomic DNA was extracted from each cell line using a genomic DNA purification kit (CWBIO, Beijing, China) according to the manufacturer’s instructions. The purity of the extracted genomic DNA was checked by measuring the absorbance at 260 nm (A_260_), 280 nm (A_280_) and 230 nm (A_230_) with a Nanodrop 2000 spectrophotometer. Extracted genomic DNA with a A_260_/A_280_ ratio between 1.8 and 2.0 and a A_260_/A_230_ ratio over 2.0 were considered satisfactory to produce DNA reference material.

### Digestion of the genomic DNA

To improve the PCR amplification efficiency, the genomic DNA was digested by restriction digestion enzyme of *EcoR*1. Enzymatic digestion mixture comprised 5 µL 10× buffer, 2.5 µL *EcoR1* restriction enzyme, 25 µL genomic DNA, and 17.5 µL ddH_2_O. No template control was prepared by adding 25 µL 1× TE_0.1_ (10 mMTris-HCl, 0.1 mM EDTA, pH = 8.0) instead of the DNA solution, and no enzyme control was made by pipetting 2.5 µL TE_0.1_ in place of the enzyme when preparing the enzymatic master mix. The enzymatic reaction lasted for 1 h at 37 °C and inactivated for 15 min at 65 °C. After the enzymatic reaction, the DNA was diluted to suitable concentrations to be analyzed on the QX100 platform (Bio-Rad Laboratories, Inc., China).

### Droplet digital PCR measurement

Optimized TaqMan MGB probe PCR assays targeting the mutation site for wild type and 7 mutant types were conducted as described in Table S1 in the supplemental material. The ddPCR analysis was performed on a QX100 system (BioRad Laboratories, Inc., Shanghai, China). The reaction mixture was in a volume of 20 µL and comprised 10 µL of 2× ddPCR Super Mix for Probe (BioRad Laboratories, Inc., Shanghai, China), 1 µL of 5 µM primers mixture, 0.2 µL of 5 µM wild type probe labeled with VIC (Thermo Scientific, Beijing, China), 0.2 µL of 5 µM mutant probe labeled with FAM (Thermo Scientific, Beijing, China), 6.6 µL of ddH_2_O and 2 µL of template DNA with a concentration of 25 ng/µL.

The optimized PCR thermal profile was conducted on a conventional PCR machine (Vetiti, Applied Biosystems). Thermal cycling consisted of a 10 min activation period at 95 °C followed by 40 cycles of a two-step thermal profile of 15 s at 95 °C denaturation and 60 s at 60 °C for combined annealing-extension and 1 cycle of 98 °C for 10 min. All samples were analyzed in three replicates. Results were analyzed with the QuantaSoft v.1.2.10.0 software (BioRad Laboratories, Inc., Shanghai, China). The workflow and data analysis were described in our previous report^25^.

### Next generation sequencing (NGS)

Two cell lines DNA, NCI-H157 and A549, were used for the optimization of NGS library preparation, including different PCR primer pairs and number of PCR cycle. Two primer pairs (MGN and NIM) were designed to amplify the *KRAS* gene fragments containing the mutation in codon 12 and 13 for NGS (Table S2). In order to evaluate the effect of number of PCR cycles on the allelic frequency measurement, 25 and 35 cycles were compared. The reaction was conducted in a 50 µL mixture consisting of 10 µL 5 × PCR buffer, 1 µL of 10 µM primers mixture, 4.8 µL dNTP, 1 µL Phusion Hot Start II DNA polymerase, 4 µL DNA with a concentration of 30 ng/µL and 29.2 µL ddH_2_O. The thermal cycling consisted of a 10 min activation period at 98 °C followed by 25 or 35 cycles of a three step thermal profile of denaturation for 30 s at 98 °C, annealing for 30 s at 55 °C, and extension for 30 s at 72 °C and then 1 cycle of 72 °C for 5 min. The PCR product was subjected to 2% agarose electrophoresis to view the amplicon, then was purified using Agencourt AMPure XP Beads (A63881, Beckman Coulter) according to the manufacturer’s instructions.

The sequencing primers (adaptor and index sequence listed in Table S2) were added to both ends of the fragment by a second round of PCR in a mixture composed of 10 µL 5× HF buffer (Thermo Fisher Scientific), 2 µL of 50 µM adaptor, 2 µL of 50 µM index, 4.8 µL of 2.5 mM dNTP, 1 µL Phusion Hot Start II DNA polymerase (Thermo Fisher Scientific) and 30.2 µL purified PCR product. The thermal cycle program consisted of a 10 min activation period at 98 °C followed by 10 cycles of a three step thermal profile (denaturation for 30 s at 98 °C, annealing for 30 s at 65 °C, and extension for 30 s at 72 °C) and then 1 cycle of 72 °C for 5 min. After the second round of PCR, the product was purified using Agencourt AMPure XP Beads (A63881, Beckman Coulter). The final purified DNA quantity and quality were assessed using a Qubit photometer (Thermo Fisher Scientific, Beijing, China) and a Qubit dsDNA HS (High Sensitivity) Assay Kit according to the manufacturer’s instructions. The proper quantity of DNA was loaded to the flow cell and sequencing was carried out on a NextSeq. 500 (Illumina, Sandiego, CA).

For analysis of the sequencing data, illumina sequencing adaptors with forward index and low quality reads (phred quality score <10) were trimmed by fastq_mcf. After trimming, short reads with less than 40 bp were then removed. Clean data were mapped to the human reference genome GRCh37 by BWA. To improve the accuracy, sequence consistency was evaluated between the reads with their “mates” within pairs using an in-house script, with reads filtered when differences were found between mate reads. Varscan was used to call the SNP. Finally, we annotated the assembly reads using Annovar.

## Results and Discussion

### Confirmation of *KRAS* mutation and copy number variation in the 7 tumor cell lines

Sanger sequencing was performed after the extraction and purification of DNA from the cultured cells to identify the *KRAS* mutation type and homozygosity in each of the 7 cell lines. The results of Sanger sequencing confirmed that each cell line carries its specific target *KRAS* mutation (Table S3 in the supplemental material). Cell lines RPMI-8226, SUN-C2B, NCI-H157 and HCT-116 had heterozygous mutations and Cell lines SW1573, A549 and SW620 had homozygous mutations.

To assess the *KRAS* copy number variation in each of the 7 tumor cell lines, a duplex ddPCR assay targeting *KRAS* mutant and wild type was used to report the total *KRAS* copy number concentration. In addition, a Quantifiler Human DNA Quantification kit (Thermo Scientific) was used on the QX100 to quantify a single copy target of the human telomerase reverse transcriptase gene (*hTERT*)^26^ which can serve as a reference single copy gene. The ratio of *KRAS* to *hTERT*, which clarifies the *KRAS* copy number variation (Table 1), was very close to 1 for cell line RPMI-8226, SNU-C2B, SW1573, A549, HCT116 and 293 T, indicating that no variation in *KRAS* copy number occurs. However, the ratio of *KRAS* to *hTERT* was 2 and 3 for cell line NCI-157 and SW620, respectively, suggesting that there are two and three copies of *KRAS*, respectively, in these cell lines.

**Table 1:** *KRAS* copy number and mutant alleles in digested and undigested cell line DNA determined by droplet digital PCR Table 1.

Cell line	Digested treatment			Undigested treatment			*P* value^1^	*Ratio* ^2^
	*hTERT* (copy/uL)	*KRAS* (copy/uL)	MU/(MU + WT) %	*hTERT* (copy/uL)	*KRAS* (copy/uL)	MU/(MU + WT) %		
RPMI-8226	17101 ± 174	17473 ± 274	66.61 ± 0.55	15186 ± 750	10985 ± 31	66.76 ± 1.17	6.93E-5	1.02
SNU-C2B	17401 ± 194	16451 ± 358	48.68 ± 0.29	14114 ± 705	13773 ± 175	47.89 ± 0.72	2.49E-3	0.95
NCI-157	15724 ± 530	27475 ± 525	51.32 ± 0.61	13901 ± 695	19753 ± 160	51.84 ± 0.09	3.04E-4	1.75
SW1573	16278 ± 244	16178 ± 210	99.92 ± 0.05	14125 ± 706	6184 ± 26	99.88 ± 0.05	1.84E-3	0.99
A549	20330 ± 686	21259 ± 201	99.99 ± 0.07	111819 ± 590	16498 ± 440	99.96 ± 0.02	4.26E-4	1.05
SW620	18840 ± 640	54201 ± 479	100.00 ± 0.18	16828 ± 841	42100 ± 845	99.97 ± 0.06	2.30E-4	2.88
HCT116	16286 ± 124	16049 ± 174	49.99 ± 0.04	12844 ± 642	15290 ± 15	50.36 ± 0.56	0.01	0.99
293 T	18748 ± 104	18641 ± 204	—	11572 ± 549	15682 ± 308	—	1.12E-3	0.99

KRAS copy number and mutant alleles in digested and undigested cell line DNA determined by droplet digital PCR.

^1^P value of T test for KRAS copy number between digested and undigested treatment.

^2^The ratio of KRAS copy number to hTERT copy number.

### Validation of ddPCR for *KRAS* mutation measurement

**Figure 1:**
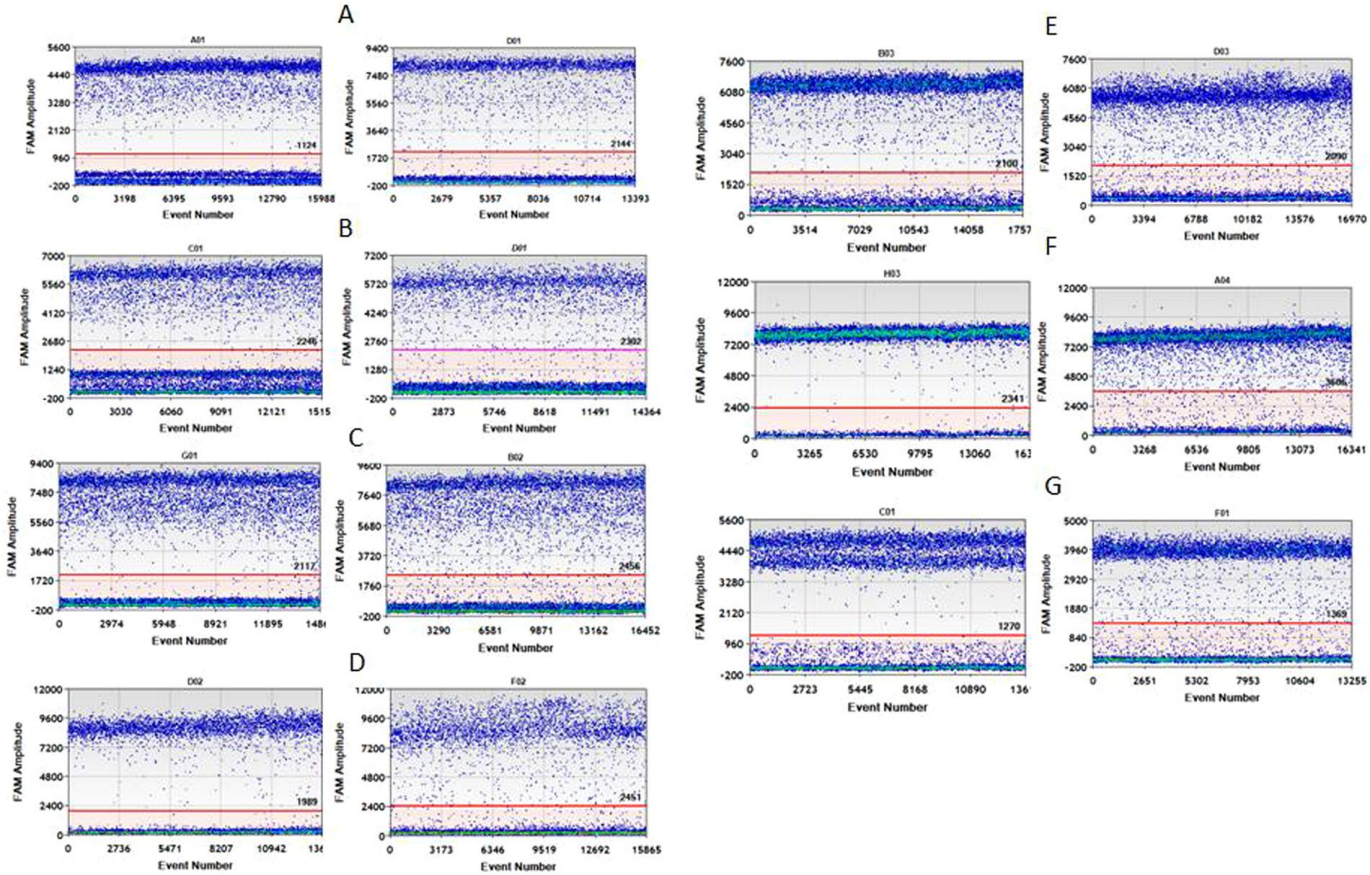
One-dimensional scatter plot for selected wells of digested (left) and undigested (right) treatment (**A**) G12A; (**B**) G12D; (**C**) G12R; (**D**) G12C; (**E**) G12S; (**F**) G12V; (**G**) G13D.

A one-dimensional scatter plot of digested and undigested genomic DNA containing each of the 7 *KRAS* mutations was obtained by ddPCR (Fig. 1). With digestion of the genomic DNA (Fig. 1. left), the amplification delay was improved greatly compared with the undigested treatments (Fig. 1. right). Additionally, the determined *KRAS* copy number (mutant + wild-type) of undigested treatment for each sample was much lower than that of the digested sample (Table 1, *P* < 0.05), which is consistent with the delay in PCR amplification for undigested treatment. This was confirmed by amplifying *hTERT* (Figure S1). These findings agreed well with previous work with plasmid DNA^25^ showing that, while enzymatic restriction can dramatically improve target accessibility. Therefore, all DNA samples were digested for the following ddPCR quantification. Interestingly, in terms of the fraction of the mutation in each cell line, no significant difference was observed between digested and undigested samples. We speculate that digestion improves the amplification of both wild type and mutant in a similar manner.

**Figure 2:**
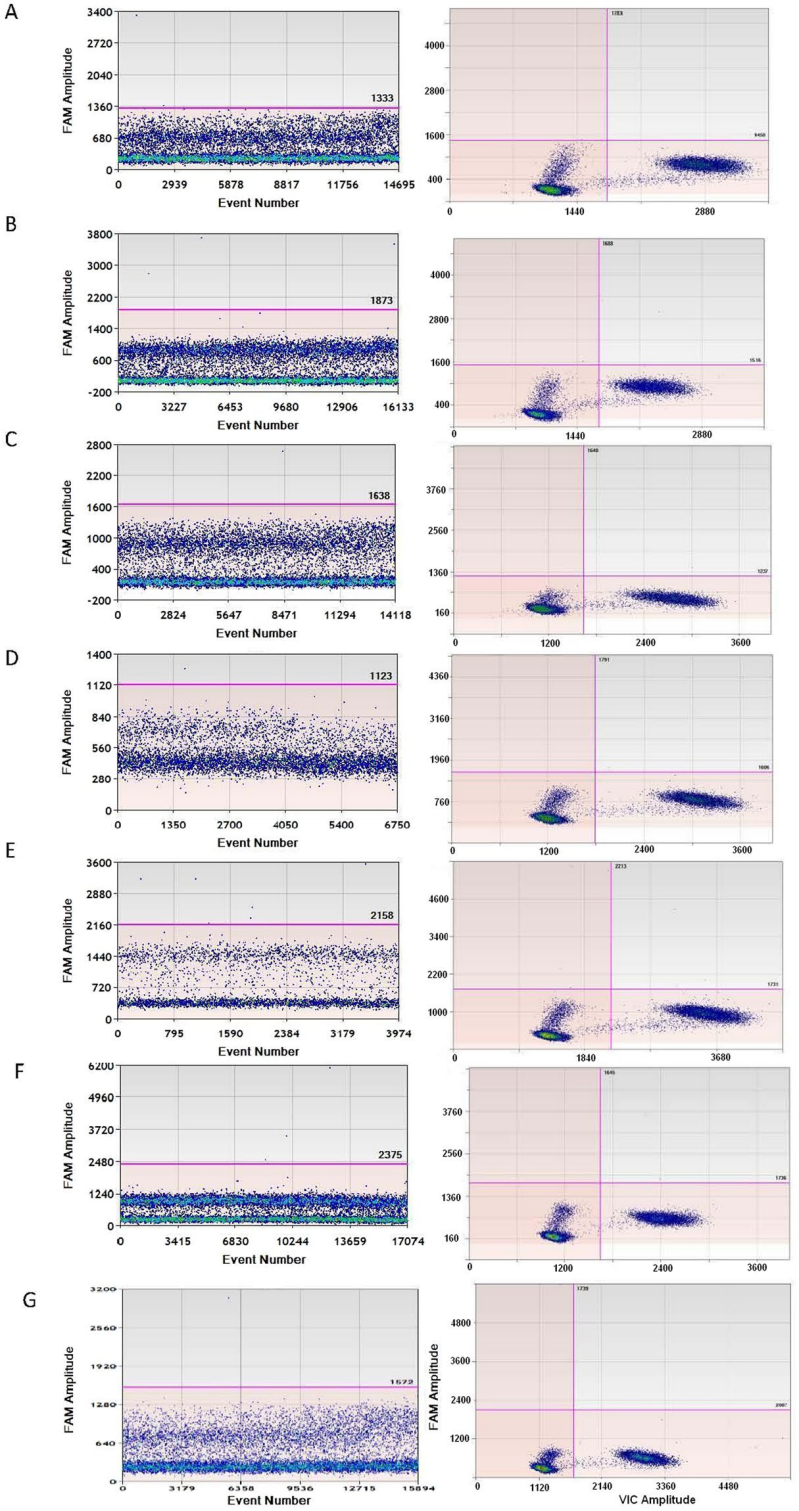
Cross reaction evaluation between wild type and each KRAS mutant by mutant specific simplex assay (left) and each mutant/wild type duplex assay (right) (**A**) G12A simplex/ duplex assay; (**B**) G12D simplex/duplex assay; (**C**) G12R simplex/duplex assay; (**D**) G12C simplex/duplex assay; (**E**) G12S simplex/duplex assay; (**F**) G12V simplex/duplex assay; (**G**) G13D simplex/ duplex assay.

Cross reaction between each mutant and wild type was evaluated by a combination of each mutant-specific FAM labeled assay with wild type DNA (293 T) and a wild type specific VIC labeled assay with each mutant DNA. No positive droplet cluster appeared for each mutant assay when adding 293 T as the template in simplex ddPCR (see Fig. 2, left), indicating no amplification when amplifying each mutant-specific assay with wild type DNA. This was confirmed by a duplex ddPCR for 293 T (Fig. 2, right) due to VIC labeled wild type rather than FAM labeled mutant being amplified.

**Figure 3:**
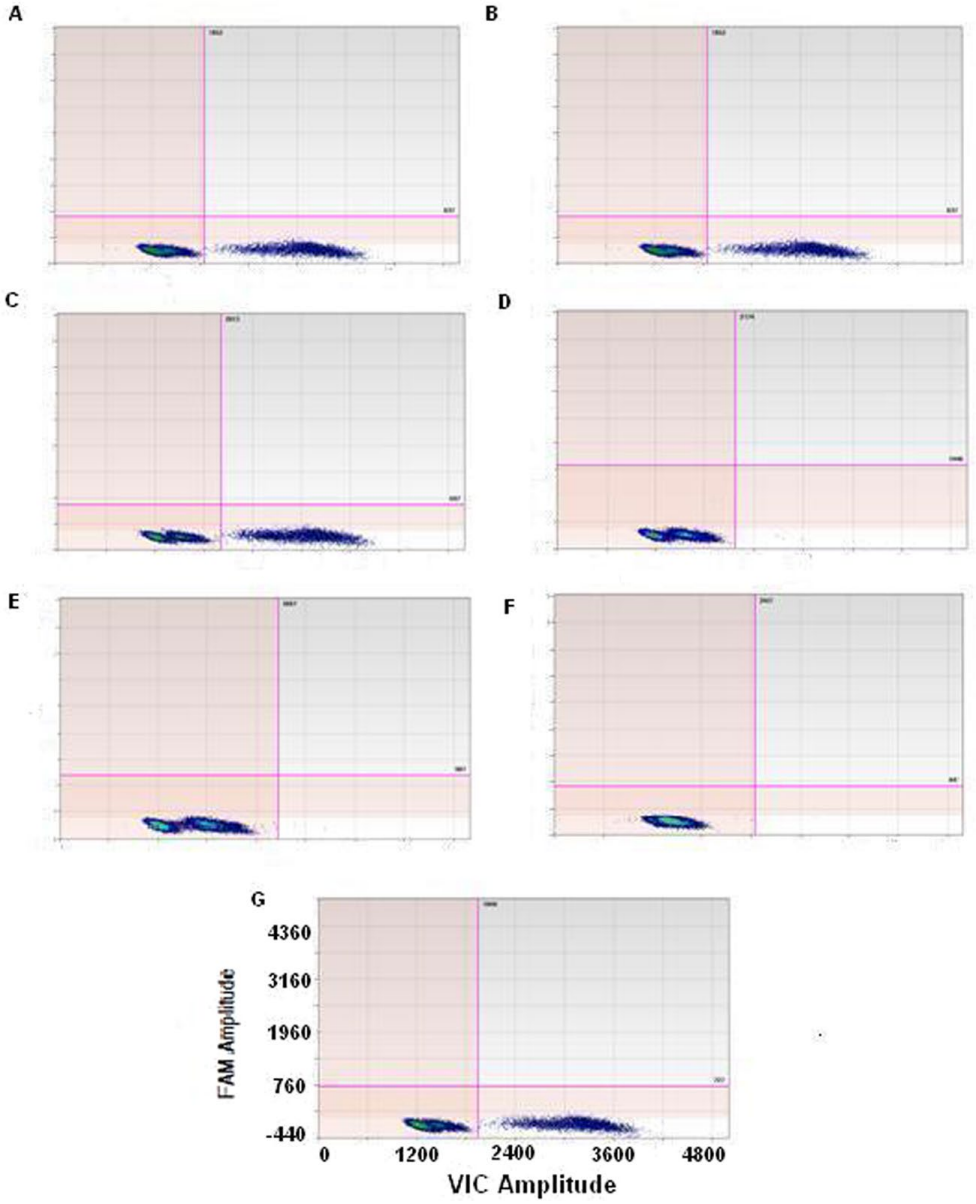
Cross reaction evaluation between wild type and each KRAS mutant by a combination of wild type specific assay and each mutant DNA (**A**) RPMI-8226; (**B**) SUN-C2B; (**C**) NCI-H157; (**D**) SW1573; (**E**) A549; (**F**) SW620; (**G**) HCT-116.

In a simplex ddPCR of wild type specific assay using each mutant DNA, no droplets were amplified for homozygous mutant cell lines of SW1573, A549 and SW620 containing G12C, G12S and G12V; however, a positive cluster in the VIC only panel appeared for the heterozygous mutant cell lines of RPMI-8226, SUN-C2B, NCI-H157 and HCT-116. These findings indicate that no cross reaction occurred when conducting the wild type specific assay with each of the 7 mutants (Fig. 3).

Detection of the 7 common KRAS mutations is particularly challenging for specificity, since they are all harbored in two adjacent codons. The goal of establishing the ddPCR assay was to characterize the reference materials containing the 7 *KRAS* mutations; therefore, it is necessary to confirm whether there is cross reactivity between the mutant assays. To evaluate the cross reaction of one mutant assay with another, each mutant specific assay separately combined with the 7 mutant DNA samples was analyzed by ddPCR (Figures S2–S8). The ddPCR limit of detection for some assays is sometimes compromised by poor discrimination of the end point signal from other clusters in a two-dimensional histogram, which can lead to false positives. For instance, in the G12R assay, the fluorescence cluster from the G12R mutation (Figure S4C) is adjacent to the cluster associated with the G12C mutation (Figure S4D). The better separation of clusters for the other assays suggests it would be less challenging to quantify mixtures of these five mutations by ddPCR.

**Figure 4:**
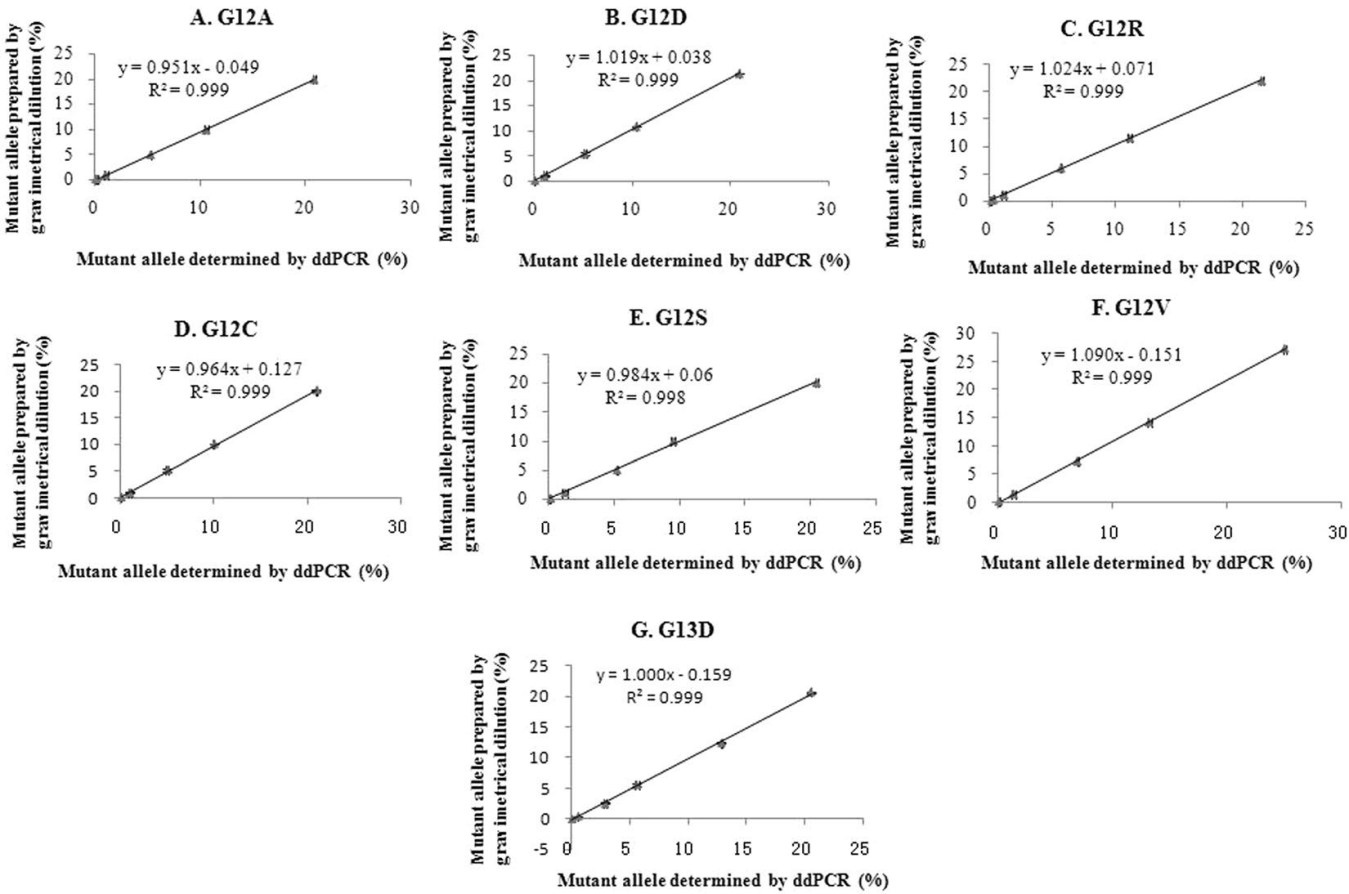
Linearity range and correlation of KRAS mutant allele measurement between gravimetrical dilution and ddPCR (**A**) G12A; (**B**) G12D; (**C**) G12R; (**D**) G12C; (**E**) G12S; (**F**) G12V; (**G**) G13D.

To demonstrate the dynamic interval of the ddPCR assays for each *KRAS* mutation, DNA isolated from each of the 7 tumor cell lines was gravimetrically diluted with wild type 293 T DNA to produce mixtures with 20%, 10%, 5%, 1% and 0.1% mutant alleles (Table S4). The theoretical mutant allele percentage of each mixture can be calculated by the *KRAS* copy number concentration of 293 T and mutant DNA and the amount of DNA of each type used to prepare the mixture. As a general characterization of all assays, the measured concentration of DNA matches the anticipated concentration over the range of 20–0.1%. A good linearity between the measured fraction of each mutant and the prepared value in each tested interval was observed for the 7 assays (R^2^ ≥ 0.999, Fig. 4). Concordance (*k*) between the two methods, ddPCR and the gravimetrical dilution, was >0.95 for all 7 mutant assays, indicating a high accuracy of ddPCR measurement for *KRAS* mutation with a level of mutant allele ≥0.1%. Therefore, the limit of quantification for all *KRAS* mutant assays was defined as 0.1%.

To determine the limit of detection of the ddPCR assay for each *KRAS* mutation, 0.05% or 0.01% mutant alleles for assay G12D and G12C were prepared. Notably, 0.01% mutant allele could be detected by ddPCR, but showed a higher percentage than anticipated (Table S4). Such results are caused by the counting of false-positive droplets; therefore, the LOD of ddPCR was based on the limit of blank (LOB), which is defined as the frequency of positive droplets measured in wild type samples or in no DNA template controls (NTCs). This method has previously been defined by Taly *et al*.^24^ on a picoliter droplet digital PCR platform. According to the wild type DNA controls in our nanoliter droplet digital PCR, the LOB of each of the 7 *KRAS* assays was 2, 4, 1, 1, 7, 3 and 1 for G12A, G12D, G12R, G12C, G12S, G12V, and G13D, respectively (Fig. 2).

### Validation of NGS measurement

Different NGS library preparations for two cell lines DNA, NCI-H157 and A549 were analyzed in Table S5. As cell line NCI-H157 was heterozygosis mutation of G12R and A549 was homozygous mutation of G12S identified by Sanger sequencing (Table S3), the theoretical allele percentage of G12R in NCI-H157 and G12S in A549 should be 50% and 100%, respectively. Library preparation with NIM PCR primer pair combing with 25 cycles results to an allele percentage of 49.72% for NCI-H157 and 99.98% for A549, which are close the theoretical values. Therefore, the primer pair of NIM and 25 cycles was chosen for the NGS library preparation.

To determine where to set the limit of detection, we first quantified the “noise” level in the identified wild type DNA (negative control) sample of 293 T. Among the six replicated measurements, the first three were from one library preparation and the rest were from another library preparation. Based on of the effects of sequencing depth on the noise level, two different levels of sequencing depths with an average of 43,000 and 196,000× were performed to measure the background (Table 2). A T test revealed that there was no significant difference in noise level for all 7 mutations between the two different depth levels and the two library preparations. The NGS noise level for G12A, G12D, G12R, G12C, G12S, G12V and G13D was 0.06%, 0.01%, 0.02%, 0.18%, 0.03%, 0.38% and 0.02%, respectively. The detection limit was defined as the mean of noise level plus 3 SD, whereas the defined limit of detection for G12A, G12D, G12R, G12C, G12S, G12V and G13D was 0.13%, 0.02%, 0.11%, 0.4%, 0.04%, 0.56% and 0.03%, respectively. This limit of detection was comparable with another different NGS platform, Ion Torrent, for *KRAS* mutation detection, for which a limit of detection of 2% was reported^17^.

**Table 2:** Sequencing noise for *KRAS* mutation by next generation sequencing.

Mutation		Replicates					
		1	2	3	4	5	6
G12S	Noise level	0.04%	0.03%	0.03%	0.03%	0.03%	0.03%
	Sequencing Depth	198412	179009	211438	42934	44453	41823
G12R	Noise level	0.01%	0.01%	0.01%	0.01%	0.02%	0.02%
	Sequencing Depth	198412	179009	211438	42934	44453	41823
G12C	Noise level	0.03%	0.20%	0.20%	0.22%	0.18%	0.23%
	Sequencing Depth	198412	179009	211438	42934	44453	41823
G12V	Noise level	0.48%	0.37%	0.33%	0.40%	0.32%	0.39%
	Sequencing Depth	198894	179017	211446	42932	44462	41830
G12D	Noise level	0.01%	0.01%	0.01%	0.02%	0.02%	0.01%
	Sequencing Depth	198894	179017	211446	42932	44462	41830
G12A	Noise level	0.09%	0.07%	0.06%	0.08%	0.04%	0.04%
	Sequencing Depth	198894	179017	211446	42932	44462	41830
G13D	Noise level	0.02%	0.02%	0.02%	0.01%	0.02%	0.01%
	Sequencing Depth	198878	179005	211425	42934	44469	41818

Given that the limit of detection for each *KRAS* mutation ranged from 0.02% to 0.56%, it was expected that all mutant allele percentages lower than this limit of detection could not be detected by NGS. Interestingly, no samples with mutation fractions lower than its corresponding detection limit were detected (Table 3). For example, for G12C, no target mutation was observed in the two samples with mutation fractions of 0.06% and 0.01%. To validate the dynamic range of NGS quantification of *KRAS* mutation, two serial dilutions containing different mutant alleles were analyzed separately (Table 3). For the two serial dilutions of G12A and G12V, the NGS result was consistent with the prepared value and the concordance rate was higher than 0.93. The linearity range for G12A and G12V of 1% to 50% (R^2^ ≥ 0.998) indicates that samples with mutant allele levels higher than 1.0% can be reliably quantified by our proposed NGS method.

**Table 3:** *KRAS* mutant alleles in serial dilution determined by next generation sequencing.

Dilution	Mutant allele percentage prepared by gravimetrical (%)	Sequence coverage	Mutant allele percentage determined by NGS (%)
G12A-S4	0.98	8684.19	0.89%
G12A-S5	0.19	8097.67	0.16
G12A-S6	0.10	7968.80	ND
G12D-S1	21.29	1075.02	20.00
G12D-S2	10.75	966.58	10.10
G12D-S3	5.41	1135.16	5.20
G12D-S4	1.06	8899.51	1.10
G12D-S5	0.10	9986.04	0.21
G12D-S7	0.01	8703.11	ND
G12C-S4	0.97	9510.11	1.0%
G12C-S6	0.06	8857.25	ND
G12C-S7	0.01	9268.47	ND
G12S-S4	1.1	8426.12	0.69
G12S-S5	0.09	8283.83	0.15
G12V-S0	59.96	950.43	61.80
G12V-S1	27.27	1091.55	25.90
G12V-S2	14.22	1133.44	14.70
G12V-S3	7.23	953.18	6.10
G12V-S4	1.53	9214.20	1.40
G12V-S5	0.17	7547.37	ND

### Characterization of *KRAS* reference material

The preparation, homogeneity and stability assessment and uncertainty evaluation is provided in the supplemental material (Tables S6–S11, Figure S9). The candidate reference material was characterized by the established ddPCR and NGS. Five vials were randomly selected from the batch and each was analyzed in three replicates. Measurements were carried out by quantifying the copy number of mutants (MU) and wide-types (WT) using an established ddPCR method (Table S12). The results of NGS of all 7 *KRAS* mutation fractions are shown in Table S13. The Shapiro-Wilk test was first used to check whether the data followed a normal distribution. Grubbs’s and Dixon’s test showed no outliers existed. Because the NGS and ddPCR were found to be in equal precision when checked by Cochran’s test, the asymmetric mean value of the NGS and ddPCR were used as the reference value according to the ISO guide 35^27^ (Table S14). The characterized fraction of mutant allele of NIM-KRAS-8 for G12A, G12D, G12R, G12C, G12S, G12V and G13D was determined to be 1.06%, 1.03%, 1.04%, 1.09%, 1.05%, 1.04% and 1.03%, respectively. The mutant allele percentage of NIM-KRAS-9 for G12A, G12D, G12R, G12C, G12S, G12V and G13D was determined to be 5.01%, 4.98%, 4.99%, 5.13%, 4.89%, 4.92% and 5.09%, respectively. The mutant allele percentage of NIM-KRAS-8 and NIM-KRAS-9 for each mutant agreed well with the preparation value determined by gravimetrical dilution, suggesting that the ddPCR and NGS measurement was accurate.

### Verification of limit of detection (LOD) of KRAS detection kits

LOD is a very important parameter, especially for clinical diagnostic methods, and should therefore be stated in the instructions of any commercial kit. The LOD of three KRAS commercial kits from different manufacturers was tested using the NIM-KRAS-8 reference material. The performance acceptance criterion for the limit of detection was established to be 95% of positive, corresponding to 19 out of 20. As a general characterization of all assays, most of the measurements for each kit were amplified with a rational Cq value (Tables S15–17). However, because the criteria for determining positive and negative results differed among the three manufacturers, the decision cannot be easily made simply by measuring amplification with a Cq.

For manufacturer A, an assay with a Cq value smaller than 35 was simply determined as positive, while a Cq value larger than 38 was deemed negative, and a Cq between 35 and 38 was regarded as a possible positive that needed to be further confirmed by repeating the measurement. However, because a well-characterized reference material of 1% of mutant allele was used in this case, all the values between 35 and 38 were considered positive. According to these criteria, there were two false negatives for assay G12V and one false negative for G12D and G13D, respectively. For the remaining assays, no false negatives were observed. In accordance with the acceptance criteria we set, the LOD of all test assays except G12V agreed with those claimed by the manufacturer A. For manufacturer B, for assay G12C, G12R, G12D, G12A, G12S, a Cq < 38 as well as Cq_sample_-Cq_control_ <8 was considered positive. For assay G13D and G12V, a Cq < 38 as well as Cq_sample_-Cq_control_ <9 was considered positive. All other values were considered negative. According to these criteria, G12A had a 100% positive rate, but a significant discrepancy was observed for all other assays, with only 5%, 65%, 60%, 20%, 5%, and 10% being positive for assay G12C, G12S, G12R, G12V, G12D and G13D, respectively. With the exception of assay G12A, the LOD for all other assays disagreed with that claimed by manufacturer B. For manufacturer C, the Cq < 38 and Cq_sample_-Cq_control_ ≤8 were considered positive for all 7 assays. According to this criterion, G12A had a 100% positive rate, while G12R and G12D had a 95% positive rate. A 50%, 10%, 25% and 10% false negative rate was observed for assays G12C, G12S, G12V and G13D, respectively. In collusion, only three assays (G12A, G12R and G12D) with a LOD of 1% were confirmed to be the same as the manufacturer claimed. After communication with each manufacturer, the discrepancy was attributed to the quality control material used to validate their assay. All the three manufacturers used their own quality control material derived from an in-house dilution of a cell line or a plasmid containing target mutant. If the value of the quality control material is not correct, for example, higher than 1%, the LOD of each assay will be overestimated, resulting in incorrect judgment criteria. Therefore, it is necessary and important to use a traceable reference material with a known correct value to validate all assays, especially those employed for clinical use. The RM characterized by two different principle methods in the present study is suitable for this purpose.

## Conclusion

Digital PCR and NGS can provide a robust and accurate quantitative measure of the fraction of *KRAS* mutant alleles in characterization of a reference material. The LOD was different from the claimed LOD of the testing kits for some assays indicates the usage of a traceable reference material was important for setting up the criteria regarding the LOD for the commercial kit. The reference material we proposed was suitable for method validation and verification of *KRAS* mutation detection.

## Electronic supplementary material

Supplemental Material
