# Supplementary material for: Evaluation of droplet digital PCR and next generation sequencing for characterizing DNA reference material for KRAS mutation detection
Source: Sci Rep. 2018 Nov 30;8:9650. doi: 10.1038/s41598-018-27368-3 (PMC6269532; doi:10.1038/s41598-018-27368-3)
Supplement: Supplementary file 1 — Supplemental Material [file 41598_2018_27368_MOESM1_ESM.doc]

**Evaluation of droplet digital PCR and next generation sequencing for characterizing DNA reference material for KRAS mutation detection**

Lianhua Dong^1^, Shangjun Wang^2^, Boqiang Fu^1^, Jing Wang^1^,

1. National Institute of Metrology, Beijing, 100013, P.R.China,

2. Nanjing Institute of Measurement and Testing Technology, Nanjing, 210049, P.R.China

**Preparation of DNA reference material**

Qualified genomic DNA materials were diluted to a concentration of 50 ng/µL with TE buffer (pH=8.0) in a 200 mL flask, then placed on a slow rotary shaker for 30 min. The solution was then gently stirred for 1 h using a magnetic stirrer, after which it was used to prepare 7 mutant mixtures of *KRAS* reference material. Two mixtures containing 1% and 5% of each *KRAS* mutant allele (NIM-KRAS-8 and NIM-KRAS-9) were prepared by weighing the proper amount of each mutant DNA and wild type DNA solution (table S6–S7). The mixture was stirred gently on a magnetic stir plate for 1 h, after which sterile 1.5 mL screw cap vials were opened and placed in a laminar flow hood in 100 unit storage boxes. Each vial was then filled with approximately 100 µL of component solution using a pipette, capped and stored at -70°C until needed.

**Homogeneity assessment**

One of the important parameter of a reference material is the homogeneity. Between-bottle homogeneity assessment of the KRAS reference materials is conducted according to ISO guide 35. The measurand was defined as the target mutant allele percentage. Eleven units were randomly selected from the batch and were analyzed by ddPCR (table S8-S11). Shapiro-Wilk test was firstly used to check if the data was following in a normal distribution. Grubbs's and Dixon's test was then applied to check if outlier existed. The results of an F-test for all RM were smaller than 2.3, the F critical value (based on 10 and 22 degrees of freedom for the between- and within-group terms). Therefore, it was firmly concluded that reference material were homogeneous within the measurement uncertainty.

**Stability assessment**

Stability of the produced batch of material was assessed using a classical stability study design following ISO guide 35. Short-term stability was conducted at -70 °C, 4°C and 25 °C for 2 weeks to simulate the transport conditions with dry ice. Long-term stability at -70 °C has been under continuous monitoring for 12 months at the time of the study. Both short- and long-term stability data were obtained from ddPCR quantification of the percentage of each KRAS mutant from three randomly selected units at each time point tested. Results were assessed for trends in the calculated percentage of mutant allele using a t-test for short term stability data and the regression slope over time for long term stability data. There was no significant change over time for the three test temperature for up to 2 weeks (p-values ranging from 0.11 to 0.58, G12R RM was shown in Figure S9).

There was no significant linear trend over time for any reference value of the KRAS reference materials when stored at -70 °C for 12 months (Table S12-13). The slop (b1) of the regression line of the measured value at each storage time and standard deviation of the slop ($s(b1)$) was calculated for 2 reference materials. According to the ISO guide 35, all 7 mutations were stable since the $\left|b1\right|$ < $s(b1)*t_{0.95,n-2}$, whereas “$t_{0.95,n-2}$” is the t-factor of T test based on n-2 degrees of freedom for the tested time point at 95% confidence level.

**Measurement uncertainty of the reference material value**

The uncertainty of the reference value (*u_T_*, *_rel_*) comprises three major components; namely, uncertainty of characterization (*u_char_*, *_rel_*), homogeneity (*u_bb, rel_*) and stability (*u_s,rel_*). The measurement uncertainty associated with batch characterization comprises several aspects related to the procedure used to quantify the mutant allele. This parameter includes the uncertainty associated with ddPCR and NGS method precision (*u_a_*, *_rel_*), as wells as the uncertainty associated with gravimetric dilutions and Type B uncertainty of a droplet volume, as previously described ^1, 2^. Uncertainty due to stability was calculated by the standard deviation of the regression coefficient ($s(b1)$) multiplying storage time (12 months) according to ISO guide 35. Uncertainty related to homogeneity accounts for the variation between units. The relative standard uncertainties were combined to give the combined relative standard uncertainty of the property value (eq 1). A summary of factors contributing to the relative standard uncertainty estimate is presented in Table S14. The combined standard uncertainty for each mutation was expanded to provide a level of confidence of 95% using a coverage factor of 2. The expanded uncertainty for each mutation of the two reference materials ranged from 4.5% to 10%.

$u_{T,rel}=\sqrt{u^{2}_{char,rel}+u^{2}_{bb,rel}+u^{2}_{s,rel}}$ (1)

Reference

1. Dong, L., Yoo, H.B., Wang, J. & Park, S.R. Accurate quantification of supercoiled DNA by digital PCR. *Scientific Reports* **6**, 24230 (2016).

2. Dong, L. *et al.* Comparison of four digital PCR platforms for accurate quantification of DNA copy number of a certified plasmid DNA reference material. *Scientific Reports* **5**, 13174-13185 (2015).

Table S1. Primer and probe sequences used in the study

| Primer/  Probe | Sequence 5’—3’ | Concentration  nM | Reference |
| --- | --- | --- | --- |
| KRAS-F | GCCTGCTGAAAATGACTGAATATAAACT | 250 | 24 |
| KRAS-R | GCTGTATCGTCAAGGCACTCTT | 250 | 24 |
| KRAS-1P | FAM-tggagctgCtggcgt-MGB | 100 | This study |
| KRAS-2P | FAM- ttggagctgAtggcgta-MGB | 100 | 23 |
| KRAS-3P | FAM-tggagctCgtggcgt-MGB | 100 | This study |
| KRAS-4P | FAM-tggagctTgtggcgta-MGB | 100 | This study |
| KRAS-5P | FAM-ttggagctAgtggcgta-MGB | 100 | This study |
| KRAS-6P | FAM-ttggagctgTtggcgta-MGB | 100 | This study |
| KRAS-7P | FAM-tggagctggtgAcgta-MGB | 100 | This study |
| WT-P | VIC-ttggagctgGtggcgta-MGB | 100 | This study |

Table S2 primer sequence used for next generation sequencing

| Name | Sequence | Amplicon (bp) | |
| --- | --- | --- | --- |
| MGN | Forward：AGTGTATTAACCTTATGTGTGACA | 140^a^ | |
|  | Reverse：TGTATCGTCAAGGCACTCTT |  |  |
| NIM | Forward：CTGGTGGAGTATTTGATAGTGTA | 282^b^ | |
|  | Reverse：TGAAAATGGTCAGAGAAACCTTTA |  |  |
| Universal | Forward: CCCTACACGACGCTCTTCCGATCT | 189^a1^ | 331^b1^ |
|  | Reverse：GTTCAGACGTGTGCTCTTCCGATCT |  |  |
| PE1.0 (adaptor) | Forward: AATGATACGGCGACCACCGAGATCTACACTCTTTCCCTACACGACGCTCTTCCGATCT | 284^a2^ | 406^b2^ |
| Index | Reverse: CAAGCAGAAGACGGCATACGAGATNNNNNNNNGTGACTGGAGTTCAGACGTGTGCTCTTCCGATC |  |  |

^a^, the length of PCR product amplified by MGN;

^b^, the length of PCR product amplified by NIM;

^a1^, the length of first round PCR product amplified with MGN+Universal;

^a2^, the length of second round PCR product amplified with MGN+Universal;

^b1^, the length of first round PCR product amplified with NIM+Universal;

^b2^: the length of second round PCR product amplified with NIM+Universal.

Table S3. *KRAS* mutant type in seven cell lines confirmed by Sanger sequencing

| **Cell lines** | ***KRAS* gene Chr12-25398285** | |
| --- | --- | --- |
| **RPMI-8226** | 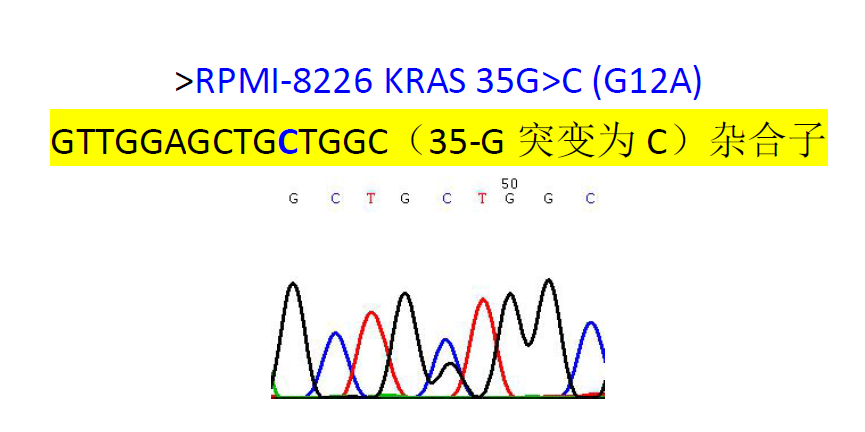 | |
| **SUN-C2B** | 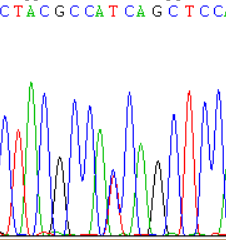 | |
| **NCI-157** | 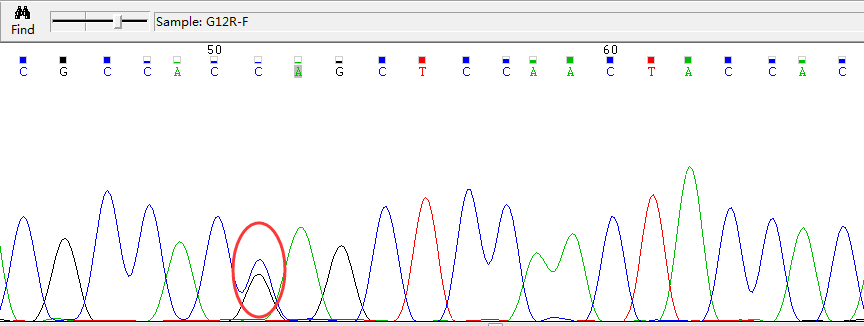 | |
| **SW1573** | 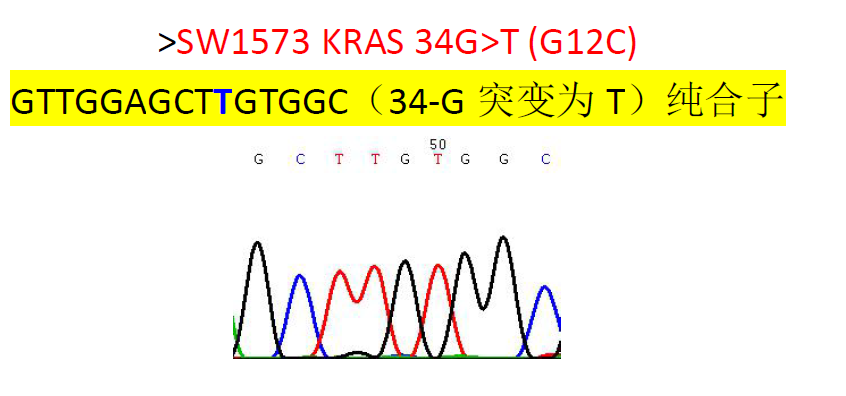 | |
| **A549** | 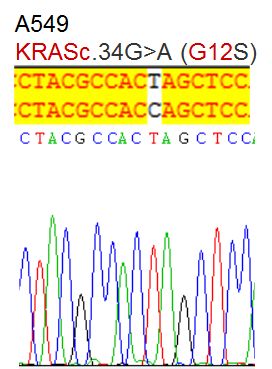 | |
| **SW620** | 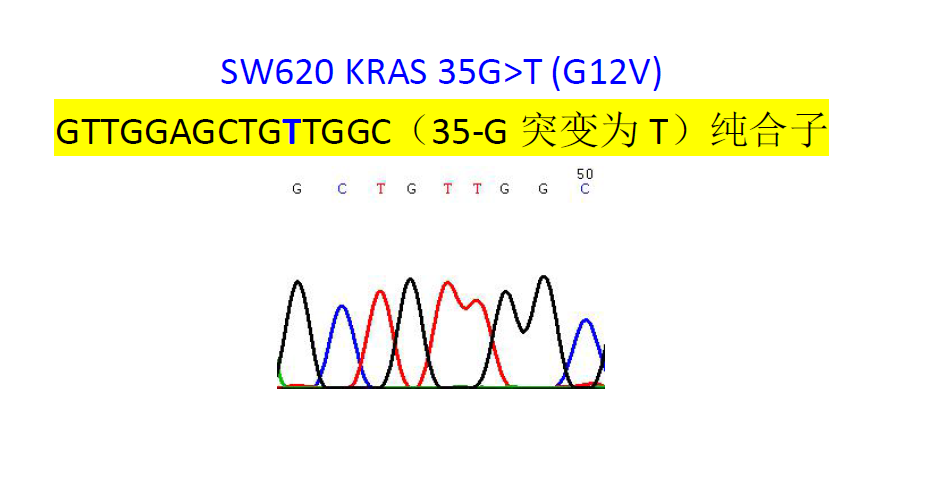 | |
| **HCT-116** | 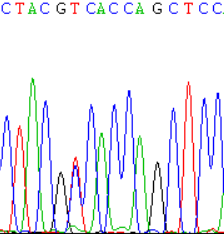 | |
| **293T** | | 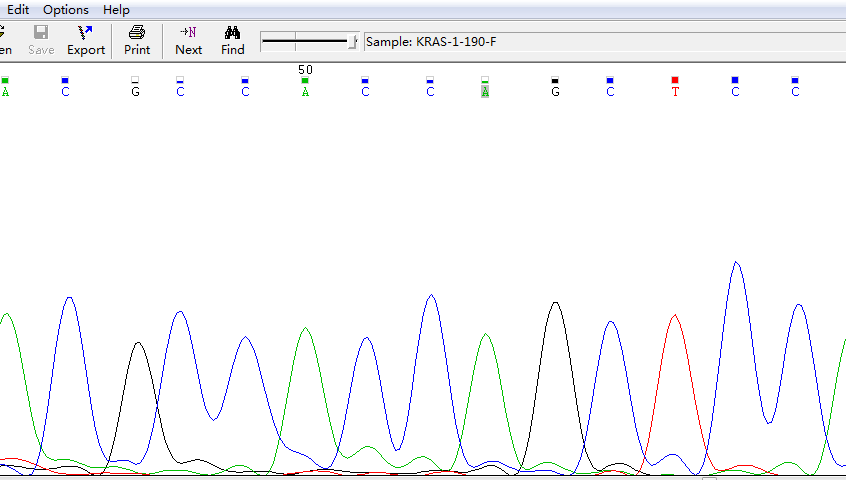 |

Table S4. Preparation of *KRAS* mutant allele in wild-type by gravimetrical dilution and determination by ddPCR

| Dilution | 293T  (mg) | Mutant DNA (mg) | Mutant allele percentage prepared by gravimetric^#^（%） | Mutant allele percentage determined by  ddPCR（%） |
| --- | --- | --- | --- | --- |
| G12A-S1 | 253.93 | 99.42 | 19.84 | 20.81 |
| G12A-S2 | 307.35 | 49.36 | 9.87 | 10.57 |
| G12A-S3 | 267.69 | 19.77 | 4.94 | 5.32 |
| G12A-S4 | 356.82 | 4.93 | 0.98 | 1.05 |
| G12A-S5 | 202.23 | 49.47 | 0.19 | 0.21 |
| G12A-S6 | 450.91 | 49.78 | 0.10 | 0.10 |
| G12D-S1 | 117.02 | 100.04 | 21.29 | 20.91 |
| G12D-S2 | 165.31 | 49.84 | 10.75 | 10.40 |
| G12D-S3 | 152.75 | 20.09 | 5.41 | 5.19 |
| G12D-S4 | 210.46 | 4.91 | 1.06 | 1.07 |
| G12D-S5 | 191.11 | 19.64 | 0.10 | 0.11 |
| G12D-S6* | 201.75 | 9.91 (S4) | 0.05 | 0.08 |
| G12D-S7* | 89.64 | 10.15 (S5) | 0.01 | 0.04 |
| G12R-S1 | 220.52 | 99.56 | 22.02 | 21.53 |
| G12R-S2 | 289.67 | 49.98 | 11.55 | 11.09 |
| G12R-S3 | 259.94 | 20.51 | 6.03 | 5.63 |
| G12R-S4 | 350.98 | 4.9 | 1.18 | 1.11 |
| G12R-S5 | 80.37 | 19.87 | 0.27 | 0.30 |
| G12R-S6 | 180.42 | 19.91 | 0.14 | 0.14 |
| G12C-S1 | 166.23 | 103.05 | 20.17 | 20.93 |
| G12C-S2 | 187.59 | 51.2 | 10.01 | 10.02 |
| G12C-S3 | 158.8 | 21.33 | 5.19 | 5.05 |
| G12C-S4 | 207.43 | 4.97 | 0.97 | 1.01 |
| G12C-S5 | 177.4 | 19.56 | 0.11 | 0.12 |
| G12C-S6* | 180.4 | 10.26 | 0.06 | 0.06 |
| G12C-S7* | 470.94 | 4.94 (S5) | 0.01 | 0.03 |
| G12S-S1 | 461.86 | 99.85 | 19.96 | 20.42 |
| G12S-S2 | 520.59 | 49.71 | 9.93 | 9.48 |
| G12S-S3 | 437.37 | 20.01 | 5.01 | 5.16 |
| G12S-S4 | 570.12 | 5.54 | 1.11 | 1.19 |
| G12S-S5 | 224.01 | 19.69 | 0.09 | 0.10 |
| G12V-S1 | 982.15 | 99.72 | 27.27 | 25.06 |
| G12V-S2 | 1104.79 | 49.58 | 14.22 | 13.25 |
| G12V-S3 | 933.43 | 19.71 | 7.23 | 7.00 |
| G12V-S4 | 1216.43 | 5.11 | 1.53 | 1.46 |
| G12V-S5 | 181.03 | 20.01 | 0.17 | 0.18 |
| G13D-S1 | 220.23 | 169.12 | 20.62 | 20.56 |
| G13D-S2 | 219.57 | 79.42 | 12.42 | 12.89 |
| G13D-S3 | 288.96 | 39.74 | 5.58 | 5.64 |
| G13D-S4 | 258.91 | 15.2 | 2.55 | 2.90 |
| G13D-S5 | 479.16 | 5.34 | 0.50 | 0.60 |
| G13D-S6 | 80.13 | 14.62 | 0.08 | 0.08 |

*Dilution used for testing the LOD.

^#^ calculated by (*C_MU_* **m_MU_*)/(C_MU_* *m_MU_*+ *C_293_** *m_293_* +*C_WT_** *m_MU_*)*100%, where *C_MU_* is the *KRAS* mutant copy number concentration in each mutant DNA, *m_MU_* is the amount of each mutant DNA, C_293_ is the *KRAS* wild type copy number concentration in 293T DNA, *m_293_* is the amount of 293T DNA, *C_WT_* is the *KRAS* wild type copy number concentration in each mutant DNA.

Table S5. Result of different NGS library preparation

| Sample | Protocol | Mutant | Primer pair | Replicates 1  AF% | Replicates 2  AF% | Replicates 3  AF% |
| --- | --- | --- | --- | --- | --- | --- |
| NCI-H157 | Protocol 1  (25cycles) | G12R | MGN-2 | 44.47% | 44.69% | 44.60% |
|  |  | G12R | NIM-1 | 49.67% | 49.79% | 49.69% |
|  | Protocol 2 (35cycles) | G12R | MGN-2 | 34.41% | 36.19% | 35.70% |
|  |  | G12R | NIM-1 | 45.93% | 46.88% | 45.07% |
| A549 | Protocol 1 (25cycles) | G12S | MGN-2 | 99.95% | 99.90% | 99.92% |
|  |  | G12S | NIM-1 | 99.94% | 99.95% | 99.98% |
|  | Protocol 2 (35cycles) | G12S | MGN-2 | 99.87% | 99.94% | 99.88% |
|  |  | G12S | NIM-1 | 99.89% | 99.95% | 99.98% |

Table S6. Preparation of candidate reference material (KRAS-8) of seven *KRAS* mutant with 1% mutant alleles

| Cell line | Mutation | Amount *m*_x_  (mg) | In each cell line | | In the mixture | | Mutant alleles in the mixture (%) |
| --- | --- | --- | --- | --- | --- | --- | --- |
|  |  |  | Mutant con. *C*_MU-x_ (cp/mg) | Wild type con. *C*_WT-x_ (cp/mg) | Mutant copy number (cp) | Wild type copy number (cp) |  |
| SW1573 | 12C | 241 | 16178 | 0 | 3898898 | 0 | 1.0 |
| SNU-C2B | 12D | 517 | 7515 | 8937 | 3885257 | 4620412 | 1.0 |
| NCI-H157 | 12R | 278 | 14006 | 13898 | 3893704 | 3863570 | 1.0 |
| A549 | 12S | 209 | 18599 | 0 | 3887176 | 0 | 1.0 |
| SW620 | 12V | 66 | 59435 | 0 | 3922732 | 0 | 1.0 |
| HTC116 | 13D | 514 | 7570 | 7759 | 3890980 | 3988126 | 1.0 |
| RPMI-8226 | 12A | 334 | 11633 | 5833 | 3885335 | 1948226 | 1.0 |
| 293T | WT | 21248 | 0 | 16315 | 0 | 346661120 | 93.0 |
| Total | - | 23407 | - | - | 27264081 | 361081454 | 100 |

Each mutant allele in the mixture (%) was calculated by：

*m*_x_**C*_MU-x_/(*m*_12C_**C*_MU-12C_+*m*_12D_**C*_MU-12D_+*m*_12D_**C*_WT-12D_+*m*_12R_**C*_MU-12R_+*m*_12R_**C*_WT-12R_ +*m*_12S_**C*_MU-12S_+*m*_12V_**C*_MU-12V_+*m*_13D_**C*_MU-13D_+*m*_13D_**C*_WT-13D_+*m*_12A_**C*_MU-12A_+*m*_12A_**C*_WT-12A_+ *m*_293T_**C*_WT-293T_)*100%

Table S7. Preparation of candidate reference material (KRAS-9) of seven *KRAS* mutant with 5% mutant alleles

| Cell line | Mutation | Amount  *m*_x_  (mg) | In each cell line | | In the mixture | | Mutant alleles in the mixture (%) |
| --- | --- | --- | --- | --- | --- | --- | --- |
|  |  |  | Mutant con. *C*_MU-x_ (cp/mg) | Wild type con. *C*_WT-x_ (cp/mg) | Mutant copy number (cp) | Wild type copy number (cp) |  |
| SW1573 | 12C | 699 | 16178 | 0 | 11308422 | 0 | 5.0 |
| SNU-C2B | 12D | 1506 | 7515 | 8937 | 11317595 | 13459072 | 5.0 |
| NCI-H157 | 12R | 807 | 14006 | 13898 | 11302946 | 11215472 | 5.0 |
| A549 | 12S | 608 | 18599 | 0 | 11308149 | 0 | 5.0 |
| SW620 | 12V | 192 | 59435 | 0 | 11411584 | 0 | 5.0 |
| HTC116 | 13D | 1496 | 7570 | 7759 | 11324720 | 11607464 | 5.0 |
| RPMI-8226 | 12A | 973 | 11633 | 5833 | 11318655 | 5675521 | 5.0 |
| 293T | WT | 6538 | 0 | 16315 | 0 | 1.07E+08 | 65.0 |
| Total | - | 12819 | - | - | 27264081 | 361081454 | 100 |

Each mutant allele % was calculated by：

*m*_x_**C*_MU-x_/(*m*_12C_**C*_MU-12C_+*m*_12D_**C*_MU-12D_+*m*_12D_**C*_WT-12D_+*m*_12R_**C*_MU-12R_+*m*_12R_**C*_WT-12R_ +*m*_12S_**C*_MU-12S_+*m*_12V_**C*_MU-12V_+*m*_13D_**C*_MU-13D_+*m*_13D_**C*_WT-13D_+*m*_12A_**C*_MU-12A_+*m*_12A_**C*_WT-12A_+ *m*_293T_**C*_WT-293T_)*100%

Table S8. Homogeneity assessment of NIM-KRAS-8

| Units | G12A（%） | | G12D（%） | | G12R（%） | | G12C（%） | | G12S（%） | | G12V（%） | | G13D（%） | |
| --- | --- | --- | --- | --- | --- | --- | --- | --- | --- | --- | --- | --- | --- | --- |
| 1 | 1.09 | 1.12 | 1.02 | 1.00 | 1.01 | 1.03 | 1.02 | 1.03 | 1.15 | 1.15 | 1.14 | 1.08 | 1.01 | 1.03 |
| 2 | 1.10 | 1.10 | 0.95 | 1.05 | 1.03 | 1.02 | 1.04 | 1.04 | 1.05 | 1.17 | 0.98 | 1.05 | 1.05 | 1.01 |
| 3 | 0.97 | 1.08 | 0.99 | 1.03 | 0.92 | 1.16 | 1.14 | 1.02 | 1.07 | 1.06 | 1.04 | 1.15 | 1.03 | 1.05 |
| 4 | 1.08 | 1.10 | 1.19 | 1.00 | 1.08 | 1.26 | 1.04 | 1.07 | 1.08 | 1.09 | 1.08 | 1.06 | 1.05 | 1.10 |
| 5 | 1.08 | 1.10 | 0.99 | 1.03 | 1.16 | 1.01 | 1.05 | 1.05 | 1.05 | 1.09 | 1.05 | 1.03 | 1.06 | 1.02 |
| 6 | 1.10 | 0.99 | 1.09 | 1.01 | 1.01 | 1.00 | 1.04 | 1.15 | 1.06 | 1.01 | 1.06 | 1.06 | 1.03 | 1.04 |
| 7 | 1.19 | 1.19 | 1.05 | 1.05 | 0.99 | 1.15 | 1.04 | 1.04 | 1.07 | 1.08 | 1.00 | 1.09 | 1.07 | 1.02 |
| 8 | 1.10 | 1.10 | 1.01 | 1.05 | 1.12 | 1.02 | 1.02 | 1.05 | 1.07 | 1.09 | 1.00 | 1.02 | 1.01 | 0.98 |
| 9 | 1.10 | 1.10 | 1.09 | 1.05 | 1.15 | 1.02 | 1.05 | 1.14 | 1.06 | 1.08 | 1.05 | 1.06 | 0.95 | 1.04 |
| 10 | 1.11 | 1.10 | 1.03 | 1.00 | 1.12 | 1.01 | 1.05 | 1.05 | 1.05 | 1.09 | 1.01 | 1.06 | 1.02 | 0.97 |
| 11 | 1.02 | 1.19 | 1.00 | 1.05 | 1.08 | 1.03 | 1.04 | 1.14 | 1.07 | 1.08 | 0.98 | 1.04 | 1.05 | 1.02 |
| Q_1_ | 0.0337 | | 0.0177 | | 0.0395 | | 0.0132 | | 0.0168 | | 0.0218 | | 0.0124 | |
| V_1_ | 10 | | 10 | | 10 | | 10 | | 10 | | 10 | | 10 | |
| S_1_^2^ | 0.0034 | | 0.0018 | | 0.0040 | | 0.0013 | | 0.0017 | | 0.0022 | | 0.0012 | |
| Q_2_ | 0.0274 | | 0.0313 | | 0.0901 | | 0.0232 | | 0.0106 | | 0.0181 | | 0.0107 | |
| V_2_ | 11 | | 11 | | 11 | | 11 | | 11 | | 11 | | 11 | |
| S_2_^2^ | 0.0025 | | 0.0028 | | 0.0082 | | 0.0021 | | 0.0009 | | 0.0016 | | 0.0009 | |
| F | 1.35 | | 0.62 | | 0.48 | | 0.63 | | 1.74 | | 1.33 | | 1.27 | |
| F_0.05(10,22)_ | 2.85 | | 2.85 | | 2.85 | | 2.85 | | 2.85 | | 2.85 | | 2.85 | |
|  | 2.2% | | 2.4% | | 3.9% | | 2.0% | | 1.4% | | 1.8% | | 1.4% | |

Table S9. Homogeneity assessment of NIM-KRAS-9

| Units | G12A（%） | | G12D（%） | | G12R（%） | | G12C（%） | | G12S（%） | | G12V（%） | | G13D（%） | |
| --- | --- | --- | --- | --- | --- | --- | --- | --- | --- | --- | --- | --- | --- | --- |
| 1 | 5.04 | 4.98 | 4.86 | 5.06 | 5.13 | 5.06 | 5.24 | 5.09 | 4.72 | 4.64 | 4.93 | 4.86 | 5.15 | 5.32 |
| 2 | 4.92 | 5.12 | 4.94 | 4.96 | 4.89 | 4.99 | 5.14 | 5.24 | 4.63 | 4.83 | 4.88 | 4.75 | 5.17 | 5.46 |
| 3 | 4.75 | 5.03 | 5.10 | 5.15 | 5.11 | 4.68 | 5.21 | 5.19 | 4.54 | 5.21 | 4.55 | 5.03 | 5.25 | 5.18 |
| 4 | 5.29 | 4.98 | 5.02 | 4.94 | 4.98 | 5.05 | 5.22 | 5.16 | 5.22 | 4.68 | 4.85 | 5.25 | 5.44 | 5.23 |
| 5 | 5.12 | 4.95 | 4.64 | 5.15 | 5.07 | 5.06 | 5.29 | 5.24 | 4.68 | 4.38 | 4.71 | 4.84 | 5.19 | 5.31 |
| 6 | 5.02 | 5.37 | 4.94 | 4.78 | 5.12 | 4.99 | 5.04 | 5.22 | 4.49 | 4.89 | 4.79 | 4.77 | 5.58 | 5.02 |
| 7 | 5.30 | 5.26 | 5.06 | 4.78 | 4.89 | 5.18 | 5.25 | 5.21 | 4.39 | 4.98 | 4.97 | 4.98 | 5.18 | 5.25 |
| 8 | 5.02 | 4.95 | 5.06 | 4.64 | 5.06 | 4.48 | 5.23 | 5.17 | 5.08 | 4.93 | 4.98 | 4.50 | 5.23 | 5.44 |
| 9 | 4.99 | 5.20 | 4.95 | 5.15 | 4.99 | 5.01 | 5.14 | 5.06 | 4.89 | 4.39 | 5.22 | 4.92 | 5.19 | 5.32 |
| 10 | 5.08 | 4.75 | 5.15 | 4.78 | 5.18 | 5.02 | 5.34 | 5.14 | 4.93 | 4.79 | 4.84 | 4.88 | 5.58 | 5.34 |
| 11 | 5.02 | 5.27 | 4.78 | 4.96 | 4.87 | 5.12 | 5.04 | 5.19 | 4.68 | 5.07 | 4.75 | 4.88 | 5.19 | 5.34 |
| Q_1_ | 0.2815822 | | 0.1404 | | 0.1903 | | 0.0799 | | 0.4307 | | 0.2612 | | 0.1028 | |
| V_1_ | 10 | | 10 | | 10 | | 10 | | 10 | | 10 | | 10 | |
| S_1_^2^ | 0.0282 | | 0.0141 | | 0.0190 | | 0.0008 | | 0.0477 | | 0.0261 | | 0.0103 | |
| Q_2_ | 0.29575 | | 0.3995 | | 0.3653 | | 0.3374 | | 0.9146 | | 0.38425 | | 0.318 | |
| V_2_ | 11 | | 11 | | 11 | | 11 | | 11 | | 11 | | 11 | |
| S_2_^2^ | 0.0269 | | 0.0363 | | 0.0332 | | 0.0307 | | 0.0831 | | 0.0349 | | 0.0289 | |
| F | 1.05 | | 0.39 | | 0.57 | | 0.26 | | 0.52 | | 0.89 | | 0.36 | |
| F_0.05(10,22)_ | 2.85 | | 2.85 | | 2.85 | | 2.85 | | 2.85 | | 2.85 | | 2.85 | |
|  | 1.50% | | 1.77% | | 1.68% | | 1.53% | | 2.78% | | 1.77% | | 1.48% | |

Table S10. Result of long-term stability study for NIM-KRAS-8

| Time | KRAS-8 | | | | | | |
| --- | --- | --- | --- | --- | --- | --- | --- |
|  | G12A | G12D | G12R | G12C | G12S | G12V | G13D |
| 1 | 1.07% | 1.03% | 1.04% | 1.09% | 1.06% | 1.04% | 1.03% |
| 2 | 1.07% | 1.03% | 1.04% | 1.08% | 1.06% | 1.04% | 1.04% |
| 3 | 1.07% | 1.03% | 1.04% | 1.09% | 1.06% | 1.04% | 1.03% |
| 6 | 1.07% | 1.03% | 1.04% | 1.09% | 1.07% | 1.04% | 1.03% |
| 12 | 1.07% | 1.03% | 1.04% | 1.09% | 1.06% | 1.04% | 1.03% |
| $\left\vert b1\right\vert$ | 1.00E-07 | 2.00E-06 | 2.00E-07 | 1.00E-05 | 1.00E-05 | 1.00E-05 | 1.00E-05 |
| $s$ | 1.21E-04 | 5.32E-05 | 2.87E-05 | 1.29E-04 | 1.37E-04 | 8.77E-05 | 1.11E-04 |
| $s(b1)*t_{0.95,3}$ | 3.96E-05 | 1.74E-05 | 9.37E-06 | 4.22E-05 | 4.48E-05 | 2.86E-05 | 3.61E-05 |
| Conclusion | Stable | Stable | Stable | Stable | Stable | Stable | Stable |

*s*, is the standard deviation of each point of the regression line

Table S11. Result of long-term stability study for NIM-KRAS-9

| Time | KRAS-9 | | | | | | |
| --- | --- | --- | --- | --- | --- | --- | --- |
|  | G12A | G12D | G12R | G12C | G12S | G12V | G13D |
| 1 | 5.01% | 4.95% | 4.99% | 5.24% | 4.86% | 4.82% | 5.16% |
| 2 | 4.99% | 4.94% | 4.96% | 5.25% | 4.75% | 4.86% | 5.15% |
| 3 | 4.98% | 4.96% | 4.98% | 5.23% | 4.84% | 4.84% | 5.18% |
| 6 | 4.99% | 4.94% | 4.98% | 5.22% | 4.76% | 4.85% | 5.14% |
| 12 | 5.02% | 4.96% | 4.97% | 5.26% | 4.85% | 4.83% | 5.15% |
| $\left\vert b1\right\vert$ | 2.00E-05 | 2.00E-05 | 2.00E-05 | 1.00E-05 | 3.00E-05 | 1.00E-05 | 3.00E-05 |
| $s$ | 3.11E-04 | 4.31E-04 | 2.59E-04 | 4.00E-04 | 1.14E-03 | 3.42E-04 | 3.38E-04 |
| $s(b1)*t_{0.95,3}$ | 1.02E-04 | 1.41E-04 | 8.46E-05 | 1.31E-04 | 3.71E-04 | 1.12E-04 | 1.10E-04 |
| Conclusion | Stable | Stable | Stable | Stable | Stable | Stable | Stable |

Table S12 Mutant allele percentage determined by ddPCR (%)

| Replicates | | Unit 1 | | | Unit 2 | | | Unit 3 | | | Unit 4 | | | Unit 5 |  |
| --- | --- | --- | --- | --- | --- | --- | --- | --- | --- | --- | --- | --- | --- | --- | --- |
|  |  | NIM-KRAS-8-G12A | | | | | | | | | | | | |  |
| 1 | | 1.09 | | 1.06 | | | 1.02 | | | 1.10 | | | 1.10 | |  |
| 2 | | 1.10 | | 1.10 | | | 1.08 | | | 1.10 | | | 1.10 | |  |
| 3 | | 0.97 | | 1.03 | | | 1.10 | | | 0.86 | | | 1.11 | |  |
| Mean | | 1.06 | | | | | | | | | | | | |  |
| SD | | 0.07 | | | | | | | | | | | | |  |
| *u_a_* | | 0.03 | | | | | | | | | | | | |  |
| *u_a_*,*_rel_* | | 2.88 | | | | | | | | | | | | |  |
| Replicates | | NIM-KRAS-8-G12D | | | | | | | | | | | | |  |
| 1 | | 1.02 | | 1.00 | | | 1.19 | | | 1.00 | | | 1.01 | |  |
| 2 | | 0.95 | | 1.05 | | | 0.99 | | | 1.03 | | | 1.09 | |  |
| 3 | | 0.99 | | 1.03 | | | 1.09 | | | 1.01 | | | 1.03 | |  |
| Mean | | 1.03 | | | | | | | | | | | | |  |
| SD | | 0.06 | | | | | | | | | | | | |  |
| *u_a_* | | 0.03 | | | | | | | | | | | | |  |
| *u_a_*,*_rel_* | | 2.47 | | | | | | | | | | | | |  |
| Replicates | | NIM-KRAS-8-G12R | | | | | | | | | | | | |  |
| 1 | | 1.03 | | 1.02 | | | 1.16 | | | 1.01 | | | 1.06 | |  |
| 2 | | 0.99 | | 1.16 | | | 1.01 | | | 1.01 | | | 1.12 | |  |
| 3 | | 1.08 | | 1.16 | | | 0.99 | | | 1.15 | | | 1.08 | |  |
| Mean | | 1.07 | | | | | | | | | | | | |  |
| SD | | 0.07 | | | | | | | | | | | | |  |
| *u_a_* | | 0.03 | | | | | | | | | | | | |  |
| *u_a_*,*_rel_* | | 2.77 | | | | | | | | | | | | |  |
| Replicates | | NIM-KRAS-8-G12C | | | | | | | | | | | | |  |
| 1 | | 1.02 | | 1.03 | | | 1.07 | | | 1.04 | | | 1.05 | |  |
| 2 | | 1.04 | | 1.04 | | | 1.05 | | | 1.05 | | | 1.05 | |  |
| 3 | | 1.14 | | 1.02 | | | 1.15 | | | 1.14 | | | 1.04 | |  |
| Mean | | 1.06 | | | | | | | | | | | | |  |
| SD | | 0.04 | | | | | | | | | | | | |  |
| *u_a_* | | 0.02 | | | | | | | | | | | | |  |
| *u_a_*,*_rel_* | | 1.85 | | | | | | | | | | | | |  |
| Replicates | | NIM-KRAS-8-G12S | | | | | | | | | | | | |  |
| 1 | | 1.15 | | 1.09 | | | 1.09 | | | 1.08 | | | 1.06 | |  |
| 2 | | 0.89 | | 1.05 | | | 1.09 | | | 1.01 | | | 1.05 | |  |
| 3 | | 1.07 | | 1.06 | | | 1.01 | | | 1.08 | | | 0.98 | |  |
| Mean | | 1.05 | | | | | | | | | | | | |  |
| SD | | 0.06 | | | | | | | | | | | | |  |
| *u_a_* | | 0.03 | | | | | | | | | | | | |  |
| *u_a_*,*_rel_* | | 2.57 | | | | | | | | | | | | |  |
| Replicates | | NIM-KRAS-8-G12V | | | | | | | | | | | | |  |
| 1 | | 1.14 | | 1.08 | | | 1.08 | | | 1.06 | | | 1.00 | |  |
| 2 | | 0.98 | | 1.05 | | | 1.05 | | | 1.03 | | | 1.05 | |  |
| 3 | | 1.04 | | 1.15 | | | 1.06 | | | 1.06 | | | 1.01 | |  |
| Mean | | 1.06 | | | | | | | | | | | | |  |
| SD | | 0.05 | | | | | | | | | | | | |  |
| *u_a_* | | 0.02 | | | | | | | | | | | | |  |
| *u_a_*,*_rel_* | | 1.93 | | | | | | | | | | | | |  |
| Replicates | | NIM-KRAS-8-G13D | | | | | | | | | | | | |  |
| 1 | | 1.01 | | 1.03 | | | 1.05 | | | 1.1 | | | 1.01 | |  |
| 2 | | 1.05 | | 1.01 | | | 1.06 | | | 1.02 | | | 0.95 | |  |
| 3 | | 1.03 | | 1.05 | | | 1.03 | | | 1.04 | | | 1.02 | |  |
| Mean | | 1.03 | | | | | | | | | | | | |  |
| SD | | 0.03 | | | | | | | | | | | | |  |
| *u_a_* | | 0.01 | | | | | | | | | | | | |  |
| *u_a_*,*_rel_* | | 1.42 | | | | | | | | | | | | |  |
| Replicates | NIM-KRAS-9-G12A | | | | | | | | | | | | | | |
| 1 | 5.04 | | 4.98 | | | 5.29 | | | 4.98 | | | 5.02 | | | |
| 2 | 4.92 | | 5.12 | | | 5.12 | | | 4.95 | | | 4.99 | | | |
| 3 | 4.75 | | 5.03 | | | 5.02 | | | 5.37 | | | 5.08 | | | |
| Mean | 5.04 | | | | | | | | | | | | | | |
| SD | 0.15 | | | | | | | | | | | | | | |
| *u_a_* | 0.07 | | | | | | | | | | | | | | |
| *u_a_*,*_rel_* | 1.30 | | | | | | | | | | | | | | |
| Replicates | NIM-KRAS-9-G12D | | | | | | | | | | | | | | |
| 1 | 4.86 | | 5.06 | | | 5.02 | | | 4.94 | | | 5.06 | | | |
| 2 | 4.94 | | 4.96 | | | 4.64 | | | 5.15 | | | 4.95 | | | |
| 3 | 5.10 | | 5.15 | | | 4.94 | | | 4.78 | | | 5.15 | | | |
| Mean | 4.98 | | | | | | | | | | | | | | |
| SD | 0.14 | | | | | | | | | | | | | | |
| *u_a_* | 0.06 | | | | | | | | | | | | | | |
| *u_a_*,*_rel_* | 1.30 | | | | | | | | | | | | | | |
| Replicates | NIM-KRAS-9-G12R | | | | | | | | | | | | | | |
| 1 | 4.89 | | 4.99 | | | 5.07 | | | 5.06 | | | 4.99 | | | |
| 2 | 5.11 | | 4.68 | | | 5.12 | | | 4.99 | | | 5.18 | | | |
| 3 | 4.98 | | 5.05 | | | 5.01 | | | 5.18 | | | 4.87 | | | |
| Mean | 5.01 | | | | | | | | | | | | | | |
| SD | 0.13 | | | | | | | | | | | | | | |
| *u_a_* | 0.06 | | | | | | | | | | | | | | |
| *u_a_*,*_rel_* | 1.15 | | | | | | | | | | | | | | |
| Replicates | NIM-KRAS-9-G12C | | | | | | | | | | | | | | |
| 1 | 5.24 | | 5.51 | | | 5.09 | | | 5.16 | | | 5.51 | | | |
| 2 | 4.78 | | 5.05 | | | 5.06 | | | 4.87 | | | 5.19 | | | |
| 3 | 5.14 | | 4.89 | | | 5.01 | | | 5.32 | | | 4.68 | | | |
| Mean | 5.10 | | | | | | | | | | | | | | |
| SD | 0.24 | | | | | | | | | | | | | | |
| *u_a_* | 0.11 | | | | | | | | | | | | | | |
| *u_a_*,*_rel_* | 2.10 | | | | | | | | | | | | | | |
| Replicates | NIM-KRAS-9-G12S | | | | | | | | | | | | | | |
| 1 | 4.52 | | 5.21 | | | 5.01 | | | 4.48 | | | 5.17 | | | |
| 2 | 4.93 | | 4.66 | | | 5.28 | | | 5.16 | | | 4.69 | | | |
| 3 | 4.94 | | 5.01 | | | 4.96 | | | 4.69 | | | 4.93 | | | |
| Mean | 4.91 | | | | | | | | | | | | | | |
| SD | 0.25 | | | | | | | | | | | | | | |
| *u_a_* | 0.11 | | | | | | | | | | | | | | |
| *u_a_*,*_rel_* | 2.28 | | | | | | | | | | | | | | |
| Replicates | NIM-KRAS-9-G12V | | | | | | | | | | | | | | |
| 1 | 4.73 | | 4.65 | | | 4.65 | | | 5.22 | | | 4.78 | | | |
| 2 | 5.13 | | 4.99 | | | 4.91 | | | 5.41 | | | 5.12 | | | |
| 3 | 5.12 | | 4.83 | | | 4.59 | | | 4.89 | | | 4.86 | | | |
| Mean | 4.93 | | | | | | | | | | | | | | |
| SD | 0.24 | | | | | | | | | | | | | | |
| *u_a_* | 0.11 | | | | | | | | | | | | | | |
| *u_a_*,*_rel_* | 2.15 | | | | | | | | | | | | | | |
| Replicates | NIM-KRAS-9-G13D | | | | | | | | | | | | | | |
| 1 | 5.15 | | 5.02 | | | 5.44 | | | 5.23 | | | 4.79 | | | |
| 2 | 5.07 | | 5.04 | | | 5.19 | | | 4.97 | | | 5.19 | | | |
| 3 | 4.68 | | 5.18 | | | 5.08 | | | 5.02 | | | 4.89 | | | |
| Mean | 5.06 | | | | | | | | | | | | | | |
| SD | 0.19 | | | | | | | | | | | | | | |
| *u_a_* | 0.08 | | | | | | | | | | | | | | |
| *u_a_*,*_rel_* | 1.65 | | | | | | | | | | | | | | |

Table S13. Mutant allele percentage determined by Next generation sequencing (%)

| Replicates | Unit 1 | | Unit 2 | | Unit 3 | | Unit 4 | | Unit 5 |
| --- | --- | --- | --- | --- | --- | --- | --- | --- | --- |
|  | NIM-KRAS-8-G12A | | | | | | | | |
| 1 | 1.02 | 1.05 | | 1.00 | | 1.03 | | 1.16 | |
| 2 | 1.09 | 1.05 | | 1.03 | | 1.01 | | 1.03 | |
| 3 | 1.09 | 1.04 | | 1.09 | | 1.04 | | 1.21 | |
| Mean | 1.06 | | | | | | | | |
| SD | 0.06 | | | | | | | | |
| *u_a_* | 0.03 | | | | | | | | |
| *u_a_*,*_rel_* | 2.43 | | | | | | | | |
| Replicates | NIM-KRAS-8-G12D | | | | | | | | |
| 1 | 1.00 | 1.00 | | 1.02 | | 1.10 | | 1.02 | |
| 2 | 0.99 | 0.96 | | 0.99 | | 1.06 | | 0.91 | |
| 3 | 1.04 | 0.97 | | 1.02 | | 1.17 | | 1.04 | |
| Mean | 1.02 | | | | | | | | |
| SD | 0.06 | | | | | | | | |
| *u_a_* | 0.03 | | | | | | | | |
| *u_a_*,*_rel_* | 2.68 | | | | | | | | |
| Replicates | NIM-KRAS-8-G12R | | | | | | | | |
| 1 | 0.92 | 0.98 | | 0.98 | | 1.08 | | 0.98 | |
| 2 | 0.99 | 0.86 | | 0.97 | | 0.97 | | 1.17 | |
| 3 | 1.08 | 0.98 | | 0.98 | | 1.08 | | 1.08 | |
| Mean | 1.01 | | | | | | | | |
| SD | 0.08 | | | | | | | | |
| *u_a_* | 0.03 | | | | | | | | |
| *u_a_*,*_rel_* | 3.44 | | | | | | | | |
| Replicates | NIM-KRAS-8-G12C | | | | | | | | |
| 1 | 1.19 | 1.09 | | 1.10 | | 1.09 | | 1.10 | |
| 2 | 1.19 | 1.13 | | 1.12 | | 1.13 | | 1.12 | |
| 3 | 1.14 | 1.11 | | 1.11 | | 1.11 | | 1.11 | |
| Mean | 1.12 | | | | | | | | |
| SD | 0.03 | | | | | | | | |
| *u_a_* | 0.01 | | | | | | | | |
| *u_a_*,*_rel_* | 1.23 | | | | | | | | |
| Replicates | NIM-KRAS-8-G12S | | | | | | | | |
| 1 | 0.97 | 1.06 | | 1.02 | | 1.01 | | 1.12 | |
| 2 | 0.91 | 1.02 | | 1.03 | | 1.04 | | 1.03 | |
| 3 | 1.05 | 1.04 | | 1.04 | | 1.08 | | 1.14 | |
| Mean | 1.04 | | | | | | | | |
| SD | 0.05 | | | | | | | | |
| *u_a_* | 0.02 | | | | | | | | |
| *u_a_*,*_rel_* | 2.36 | | | | | | | | |
| Replicates | NIM-KRAS-8-G12V | | | | | | | | |
| 1 | 0.96 | 1.00 | | 0.97 | | 1.11 | | 0.99 | |
| 2 | 0.95 | 0.97 | | 1.06 | | 1.09 | | 1.03 | |
| 3 | 1.04 | 1.04 | | 1.01 | | 1.13 | | 1.01 | |
| Mean | 1.02 | | | | | | | | |
| SD | 0.06 | | | | | | | | |
| *u_a_* | 0.02 | | | | | | | | |
| *u_a_*,*_rel_* | 2.40 | | | | | | | | |
| Replicates | NIM-KRAS-8-G13D | | | | | | | | |
| 1 | 0.95 | 1.01 | | 1.03 | | 1.03 | | 1.03 | |
| 2 | 1.00 | 1.02 | | 1.02 | | 1.12 | | 1.02 | |
| 3 | 1.03 | 1.09 | | 0.97 | | 1.09 | | 0.97 | |
| Mean | 1.03 | | | | | | | | |
| SD | 0.05 | | | | | | | | |
| *u_a_* | 0.02 | | | | | | | | |
| *u_a_*,*_rel_* | 2.02 | | | | | | | | |
| Replicates | NIM-KRAS-9-G12A | | | | | | | | |
| 1 | 5.02 | 5.5 | | 4.61 | | 4.69 | | 4.71 | |
| 2 | 5.26 | 5.08 | | 4.9 | | 4.97 | | 4.89 | |
| 3 | 5.05 | 4.67 | | 5.04 | | 5.22 | | 5.13 | |
| Mean | 4.98 | | | | | | | | |
| SD | 0.25 | | | | | | | | |
| *u_a_* | 0.11 | | | | | | | | |
| *u_a_*,*_rel_* | 2.22 | | | | | | | | |
| Replicates | NIM-KRAS-9-G12D | | | | | | | | |
| 1 | 4.94 | 4.81 | | 5.29 | | 5.11 | | 5.23 | |
| 2 | 4.77 | 5.17 | | 4.73 | | 4.99 | | 4.93 | |
| 3 | 4.98 | 4.88 | | 4.85 | | 4.95 | | 4.88 | |
| Mean | 4.97 | | | | | | | | |
| SD | 0.17 | | | | | | | | |
| *u_a_* | 0.07 | | | | | | | | |
| *u_a_*,*_rel_* | 1.50 | | | | | | | | |
| Replicates | NIM-KRAS-9-G12R | | | | | | | | |
| 1 | 5.09 | 4.86 | | 4.90 | | 4.96 | | 4.99 | |
| 2 | 5.01 | 4.96 | | 5.15 | | 4.88 | | 5.05 | |
| 3 | 4.97 | 4.85 | | 5.00 | | 4.75 | | 5.08 | |
| Mean | 4.97 | | | | | | | | |
| SD | 0.11 | | | | | | | | |
| *u_a_* | 0.05 | | | | | | | | |
| *u_a_*,*_rel_* | 0.95 | | | | | | | | |
| Replicates | NIM-KRAS-9-G12C | | | | | | | | |
| 1 | 5.48 | 5.02 | | 5.10 | | 5.08 | | 5.03 | |
| 2 | 5.06 | 5.21 | | 5.40 | | 5.58 | | 4.91 | |
| 3 | 5.04 | 5.16 | | 4.86 | | 5.24 | | 5.12 | |
| Mean | 5.15 | | | | | | | | |
| SD | 0.20 | | | | | | | | |
| *u_a_* | 0.09 | | | | | | | | |
| *u_a_*,*_rel_* | 1.75 | | | | | | | | |
| Replicates | NIM-KRAS-9-G12S | | | | | | | | |
| 1 | 4.92 | 5.12 | | 4.93 | | 4.89 | | 4.62 | |
| 2 | 4.98 | 4.89 | | 4.60 | | 4.71 | | 4.88 | |
| 3 | 5.01 | 4.87 | | 4.80 | | 5.21 | | 4.74 | |
| Mean | 4.88 | | | | | | | | |
| SD | 0.17 | | | | | | | | |
| *u_a_* | 0.08 | | | | | | | | |
| *u_a_*,*_rel_* | 1.55 | | | | | | | | |
| Replicates | NIM-KRAS-9-G12V | | | | | | | | |
| 1 | 4.85 | 4.61 | | 4.8 | | 4.98 | | 4.98 | |
| 2 | 4.96 | 4.98 | | 4.83 | | 4.98 | | 4.83 | |
| 3 | 5.21 | 4.88 | | 5.12 | | 4.83 | | 4.93 | |
| Mean | 4.92 | | | | | | | | |
| SD | 0.14 | | | | | | | | |
| *u_a_* | 0.06 | | | | | | | | |
| *u_a_*,*_rel_* | 1.29 | | | | | | | | |
| Replicates | NIM-KRAS-9-G13D | | | | | | | | |
| 1 | 5.01 | 5.19 | | 5.48 | | 5.09 | | 5.08 | |
| 2 | 5.17 | 4.97 | | 5.21 | | 4.99 | | 5.11 | |
| 3 | 5.14 | 5.08 | | 5.15 | | 4.68 | | 5.25 | |
| Mean | 5.11 | | | | | | | | |
| SD | 0.17 | | | | | | | | |
| *u_a_* | 0.08 | | | | | | | | |
| *u_a_*,*_rel_* | 1.50 | | | | | | | | |

Table S14. Reference value, factors contributing to the relative standard uncertainty and expanded uncertainty of the *KRAS* mutant reference material

| NIM-KRAS-8 | | | | | | | |
| --- | --- | --- | --- | --- | --- | --- | --- |
| Mutant | G12A | G12D | G12R | G12C | G12S | G12V | G13D |
| RMV* (%) | **1.06** | **1.03** | **1.04** | **1.09** | **1.05** | **1.04** | **1.03** |
| *u_S_* (%) | 1.39 | 0.64 | 0.34 | 1.46 | 1.59 | 1.04 | 1.33 |
| *u_bb_* (%) | 2.20 | 2.40 | 3.90 | 2.00 | 1.40 | 1.80 | 1.40 |
| *u_c_*_har_ (%) | **4.3** | **4.2** | **4.9** | **3.1** | **4.1** | **3.8** | **3.3** |
| *u_c_* (%) | 5.06 | 4.91 | 6.28 | 3.96 | 4.62 | 4.30 | 3.80 |
| *U*（*k*=2）(%) | 10 | 10 | 13 | 8 | 10 | 9 | 8 |
| NIM-KRAS-9 | | | | | | | |
| Mutant | G12A | G12D | G12R | G12C | G12S | G12V | G13D |
| RMV (%) | **5.01** | **4.98** | **4.99** | **5.13** | **4.89** | **4.92** | **5.09** |
| *u_S_* (%) | 0.77 | 1.07 | 0.64 | 0.94 | 2.90 | 0.87 | 0.81 |
| *u_bb_* (%) | 1.50 | 1.77 | 1.68 | 1.53 | 2.78 | 1.77 | 1.48 |
| *u_c_*_har_ (%) | **3.3** | **2.9** | **2.6** | **2.8** | **2.7** | **2.5** | **2.6** |
| *u_c_* (%) | 3.7 | 3.6 | 3.2 | 3.3 | 4.8 | 3.2 | 3.1 |
| *U*（*k*=2）(%) | 8 | 8 | 7 | 7 | 10 | 7 | 7 |

*the reference value of the reference material; *u_S_*, uncertainty of the long term stability; *u_bb_*, uncertainty of homogeneity; *u_c_*_har_, uncertainty of characterization by ddPCR and NGS; *u_c_*, the combined uncertainty of the above three components.

Table S15. Verification result of limit of detection for KRAS from manufactory A

| Plate 1 | NTC | K1 | K2 | K3 | K4 | K5 | K6 | K7 | K8 | K9 | K10 | NTC |
| --- | --- | --- | --- | --- | --- | --- | --- | --- | --- | --- | --- | --- |
| 12C | N | 35.38 | 34.96 | 35.30 | 35.96 | 34.94 | 35.39 | 35.86 | 35.30 | 34.52 | 36.61 | N |
| 12S | N | 36.67 | 34.79 | 36.50 | 35.56 | 36.28 | 36.85 | 36.37 | 35.58 | 36.05 | 35.41 | N |
| 12R | N | 33.38 | 32.64 | 33.21 | 33.29 | 32.99 | 33.49 | 32.72 | 33.26 | 33.31 | 32.68 | N |
| 12V | N | 37.22 | 37.41 | 37.67 | N | 36.93 | 37.63 | N | 37.11 | 37.71 | 35.00 | N |
| 12D | N | 35.99 | 36.76 | 36.85 | 37.52 | 36.90 | 37.27 | 36.71 | 37.55 | N | 37.29 | N |
| 12A | N | 32.29 | 31.77 | 32.51 | 32.50 | 32.71 | 33.03 | 31.73 | 32.86 | 31.85 | 32.40 | N |
| 13D | N | 36.56 | 36.89 | 37.75 | 36.45 | 35.21 | 36.21 | N | 36.20 | 35.97 | 36.01 | N |
| Control | 21.37 | 21.22 | 21.41 | 21.63 | 21.56 | 21.26 | 21.25 | 21.32 | 21.30 | 21.22 | 21.27 | 21.23 |
| Plate 2 | NTC | K11 | K12 | K13 | K14 | K15 | K16 | K17 | K18 | K19 | K20 | NTC |
| 12C | N | 34.40 | 34.39 | 35.90 | 34.74 | 35.48 | 35.31 | 35.55 | 36.44 | 34.63 | 35.34 | N |
| 12S | N | 35.56 | 34.74 | 35.71 | 36.14 | 34.78 | 36.30 | 35.19 | 35.61 | 35.39 | 35.67 | N |
| 12R | N | 33.24 | 33.46 | 33.48 | 33.29 | 32.62 | 32.92 | 33.41 | 32.58 | 33.57 | 33.22 | N |
| 12V | N | 37.35 | 37.41 | 36.58 | 36.13 | 35.97 | 36.62 | 37.99 | 37.53 | 35.43 | 37.94 | N |
| 12D | N | 37.31 | 36.71 | 36.44 | 37.59 | 37.71 | 36.98 | 36.95 | 35.70 | 36.92 | 36.39 | N |
| 12A | N | 31.96 | 31.90 | 31.92 | 32.29 | 31.61 | 31.82 | 32.29 | 32.23 | 32.46 | 31.96 | N |
| 13D | N | 35.92 | 35.21 | 35.61 | 36.10 | 36.00 | 36.46 | 36.41 | 35.63 | 36.45 | 34.74 | N |
| Control | 20.50 | 21.16 | 21.18 | 21.39 | 21.37 | 21.28 | 20.82 | 20.99 | 21.36 | 20.97 | 20.94 | 21.08 |

Table S16. Verification result of limit of detection for KRAS from manufactory B

| Plate 1 | NTC | K1 | K2 | K3 | K4 | K5 | K6 | K7 | K8 | K9 | K10 | NTC |
| --- | --- | --- | --- | --- | --- | --- | --- | --- | --- | --- | --- | --- |
| 12C | N | 33.44 | 33.62 | 33.41 | 33.56 | 34.13 | 34.22 | 33.94 | 33.82 | 34.00 | 33.47 | N |
| 12S | N | 32.75 | 32.22 | 32.00 | 32.20 | 33.44 | 32.95 | 32.61 | 33.50 | 31.91 | 33.22 | N |
| 12R | N | 30.78 | 31.31 | 31.86 | 31.83 | 32.43 | 32.82 | 33.03 | 32.65 | 32.27 | 32.48 | N |
| 12V | N | 33.87 | 33.99 | 33.93 | 33.57 | 33.72 | 33.70 | 35.06 | 35.17 | 34.23 | N | N |
| 12D | N | 33.38 | 33.95 | 35.39 | 34.15 | 34.81 | 36.55 | 34.25 | 36.32 | 35.32 | 33.43 | N |
| 12A | N | 30.64 | 30.32 | 30.63 | 30.40 | 32.63 | 30.54 | 30.28 | 30.48 | 30.49 | 30.23 | N |
| 13D | N | 33.72 | 34.33 | 36.53 | 34.96 | N | N | N | 32.43 | 34.68 | 35.00 | N |
| Control | N | 24.35 | 24.34 | 24.58 | 24.88 | 24.80 | 25.43 | 24.43 | 24.67 | 24.61 | 23.65 | N |
| Plate 2 | NTC | K11 | K12 | K13 | K14 | K15 | K16 | K17 | K18 | K19 | K20 | NTC |
| 12C | N | 33.81 | 34.14 | 39.34 | 38.77 | 39.23 | 38.75 | 35.57 | 35.41 | 34.69 | 35.39 | N |
| 12S | 37.20 | 32.74 | 32.54 | 35.50 | 35.29 | 35.47 | 35.29 | 33.32 | 33.29 | 32.46 | 33.10 | N |
| 12R | N | 32.77 | 32.61 | 34.85 | 34.93 | 36.29 | 36.85 | 33.13 | 32.70 | 32.67 | 32.46 | N |
| 12V | N | 35.01 | 34.63 | 37.20 | 36.82 | 36.89 | 37.04 | 34.43 | 35.58 | 34.84 | 35.06 | N |
| 12D | N | 33.90 | 33.71 | 36.85 | 37.38 | 36.88 | 36.49 | 35.42 | 34.90 | 34.88 | 35.73 | N |
| 12A | N | 31.87 | 31.80 | 33.68 | 33.30 | 33.26 | 33.68 | 31.68 | 31.78 | 31.61 | 31.84 | N |
| 13D | N | 32.90 | 32.88 | 36.62 | 34.92 | 36.28 | 37.79 | N | N | N | N | N |
| Control | 24.53 | 25.28 | 25.32 | 32.93 | 27.55 | 27.16 | 26.86 | 25.69 | 25.78 | 25.77 | 25.68 |  |

Table S17. Verification result of limit of detection for KRAS from manufactory C

| Plate 1 | NTC | K1 | K2 | K3 | K4 | K5 | K6 | K7 | K8 | K9 | K10 | NTC |
| --- | --- | --- | --- | --- | --- | --- | --- | --- | --- | --- | --- | --- |
| 12A | N | 33.07 | 33.35 | 32.88 | 33.58 | 33.11 | 33.10 | 32.90 | 32.10 | 33.15 | 33.21 | N |
| 12D | N | 33.92 | 34.10 | 34.08 | 34.10 | 34.29 | 33.76 | 33.77 | 33.62 | 33.63 | 33.90 | N |
| 12R | N | 32.56 | 33.77 | 34.43 | 33.74 | 33.59 | 36.60 | 33.87 | 33.57 | 33.86 | 34.06 | N |
| 12C | N | 35.40 | 37.72 | 35.95 | 35.68 | 35.93 | 35.97 | 35.58 | 35.42 | 35.23 | 35.45 | N |
| 12S | N | 33.95 | 34.60 | 34.32 | 34.55 | 34.62 | 34.84 | 34.27 | 34.41 | 34.77 | 34.31 | N |
| 12V | N | 35.51 | 34.69 | 34.90 | 36.13 | 35.33 | 35.93 | 35.50 | 35.14 | 35.45 | 34.63 | N |
| 13D | N | 35.15 | 34.72 | 34.83 | 37.50 | No Ct | 34.74 | 34.67 | 33.93 | 33.90 | 33.78 | N |
| Control | N | 27.82 | 27.75 | 27.48 | 27.55 | 27.59 | 26.97 | 26.92 | 26.79 | 27.75 | 27.66 | N |
| Plate 2 | NTC | K11 | K12 | K13 | K14 | K15 | K16 | K17 | K18 | K19 | K20 | NTC |
| 12A | N | 32.74 | 32.43 | 32.92 | 32.97 | 33.09 | 32.81 | 33.08 | 32.98 | 32.97 | 31.82 | N |
| 12D | N | 33.49 | 31.48 | 34.31 | 33.97 | 33.53 | 33.58 | 33.54 | 33.55 | 33.80 | N | N |
| 12R | N | 32.80 | 33.49 | 34.34 | 34.19 | 33.00 | 35.16 | 33.47 | 34.34 | 33.20 | 31.63 | N |
| 12C | N | 35.88 | 35.80 | 36.79 | 35.48 | 34.73 | 35.12 | 34.95 | 36.29 | 35.66 | 34.35 | N |
| 12S | N | 35.56 | 34.73 | 34.88 | 34.20 | 34.27 | 33.68 | 34.06 | 33.75 | 33.84 | 32.93 | N |
| 12V | N | 33.60 | 33.75 | 34.85 | 34.40 | 34.60 | 34.36 | 33.51 | 34.23 | 34.25 | 24.10 | N |
| 13D | N | 33.91 | 32.81 | 33.69 | 34.00 | 34.56 | 34.10 | 33.95 | 33.51 | 33.87 | 30.78 | N |
| Control | 27.89 | 26.96 | 27.28 | 27.28 | 27.53 | 26.91 | 26.82 | 26.84 | 26.48 | 25.47 | 23.69 | 25.77 |


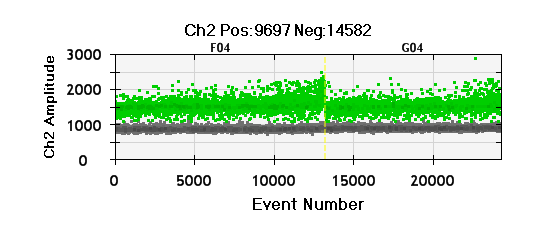

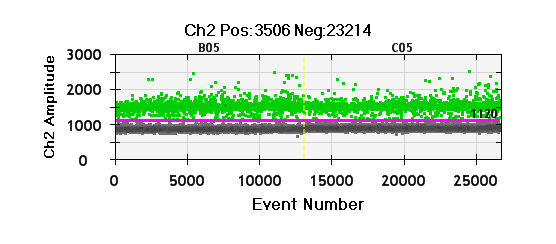


A

B

C

D


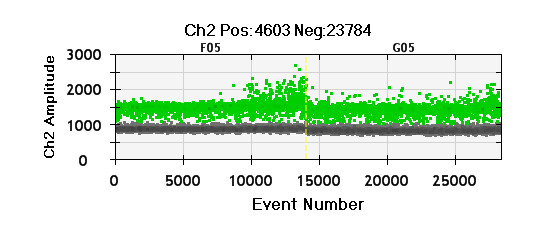

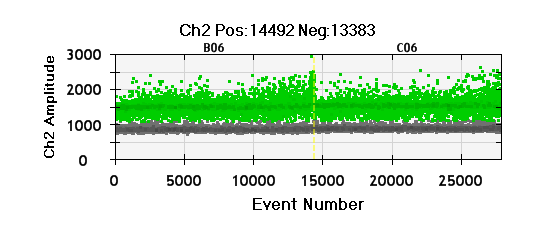


F

E


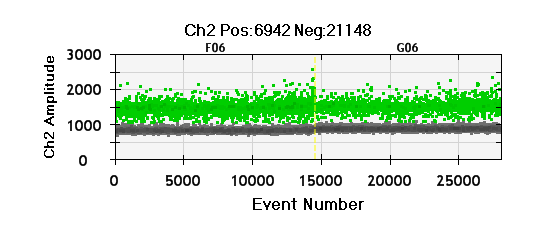

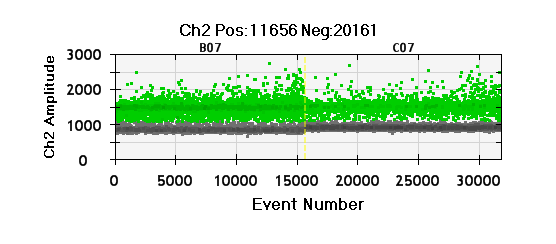


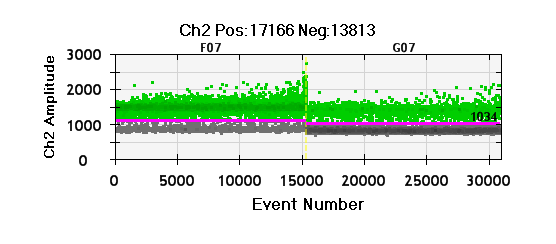


G

Figure S1. One-dimensional scatter plot for selected wells of digested (left) and undigested (right) treatment for amplify *hTERT* (A, RPMI-8226; B, SUN-C2B; C, NCI-H157; D, SW1573; E, A549; F, SW620; G, HCT-116).


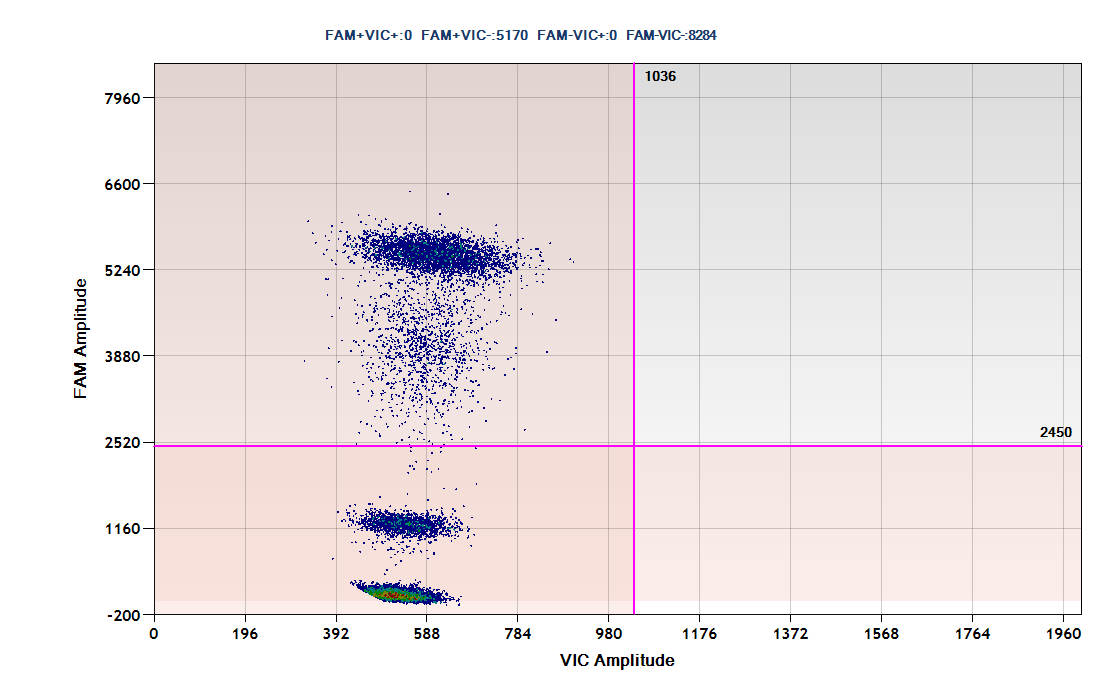

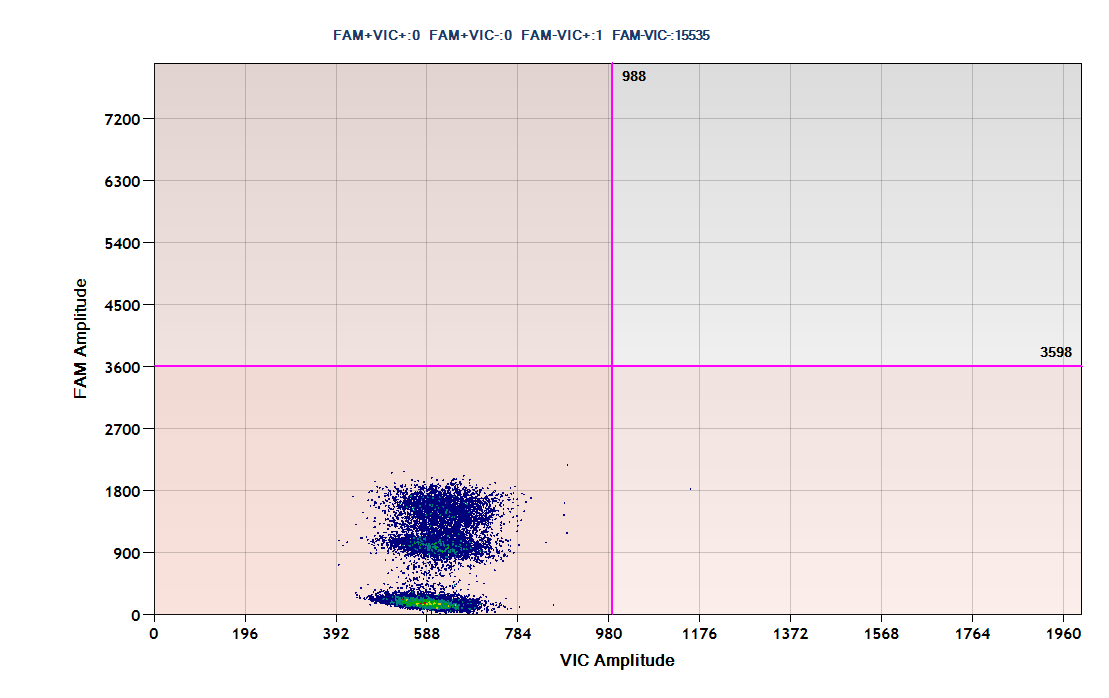

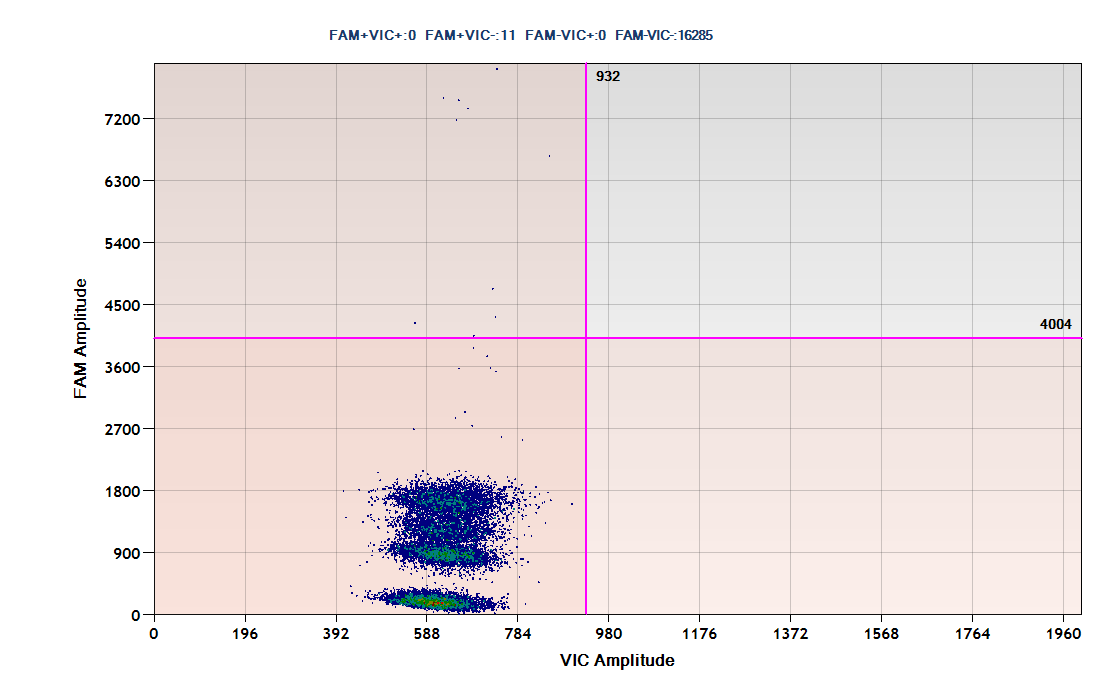

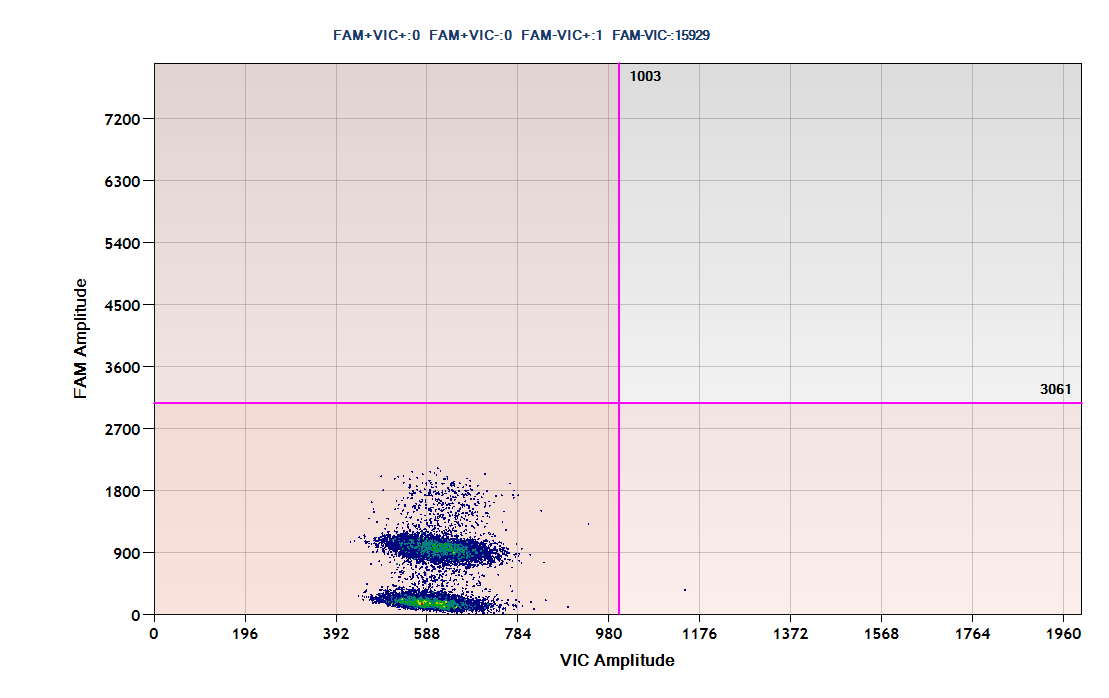


A

B

C

D

E

F


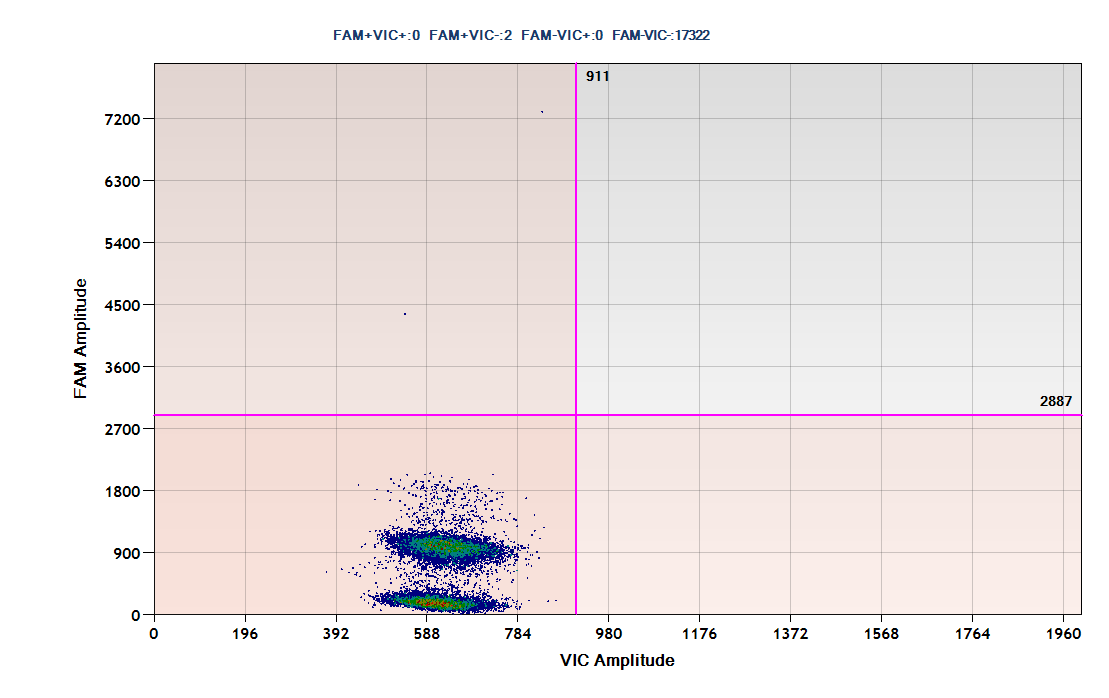

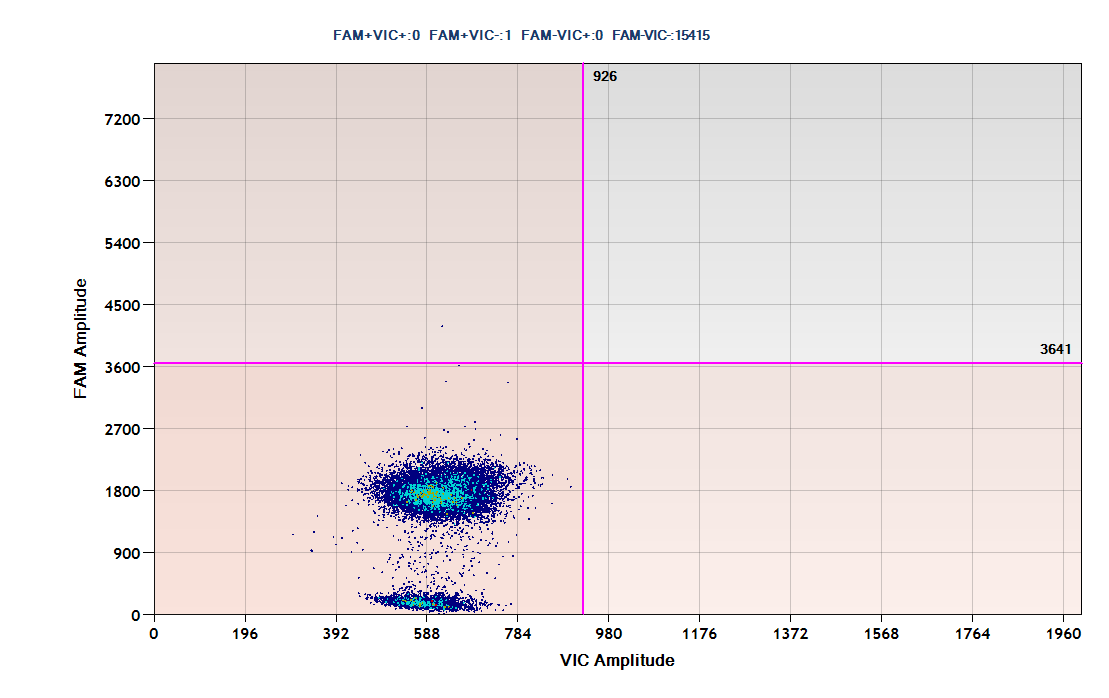


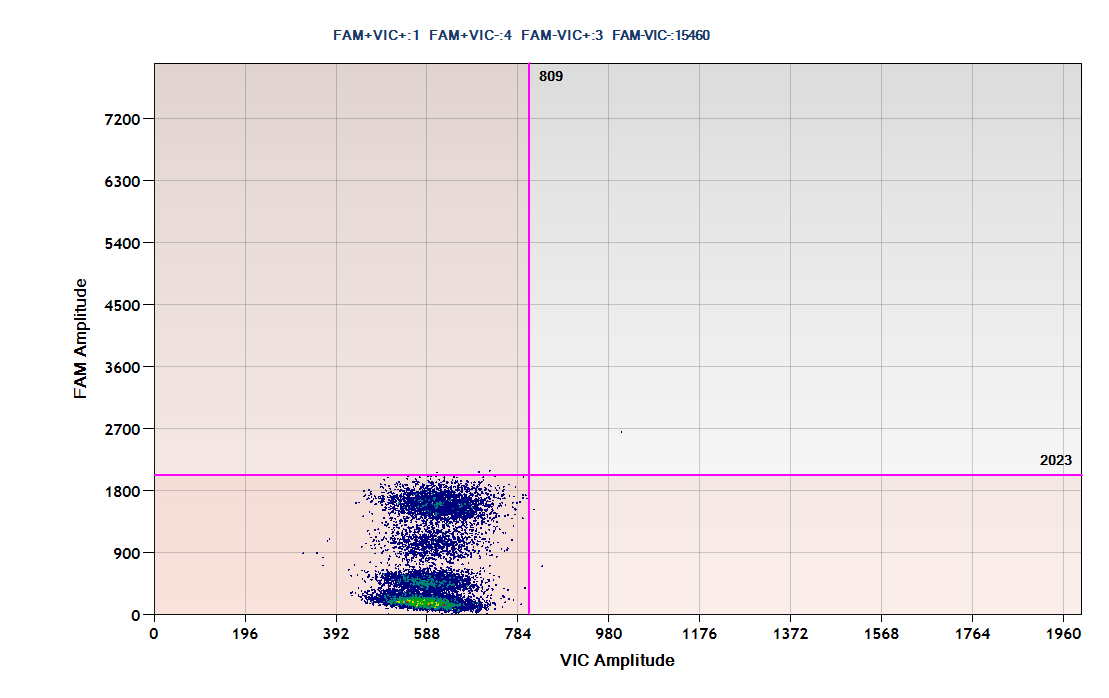


G

Figure S2. Cross reaction evaluation between G12A specific assay and each *KRAS* mutant DNA (A, RPMI-8226; B, SUN-C2B; C, NCI-H157; D, SW1573; E, A549; F, SW620; G, HCT-116).


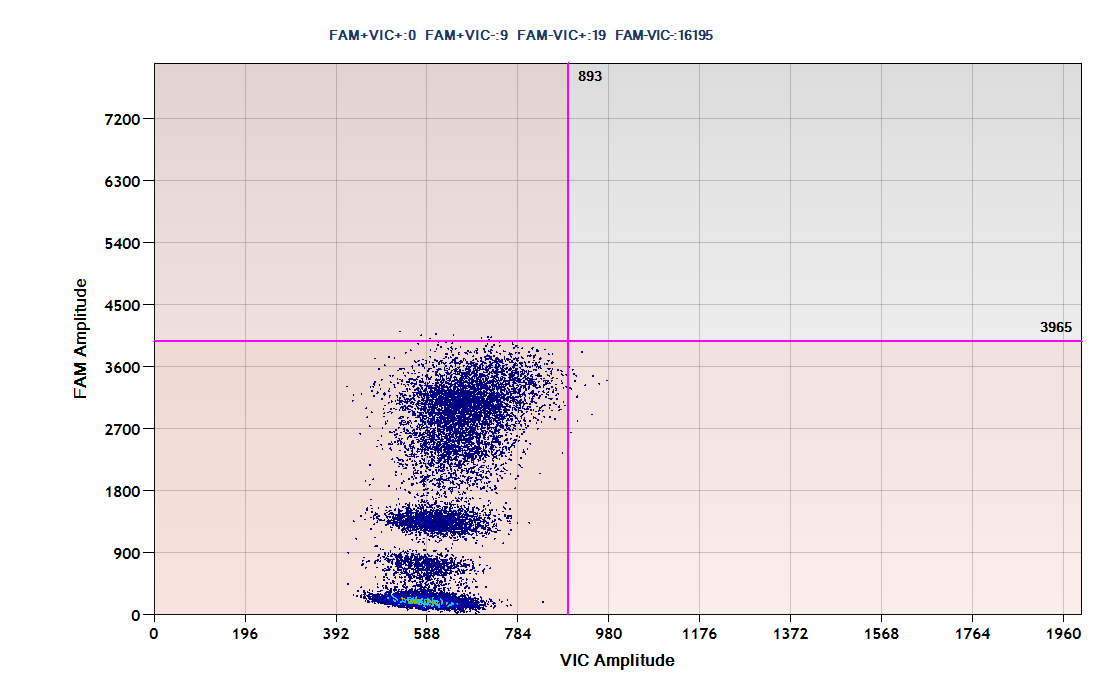

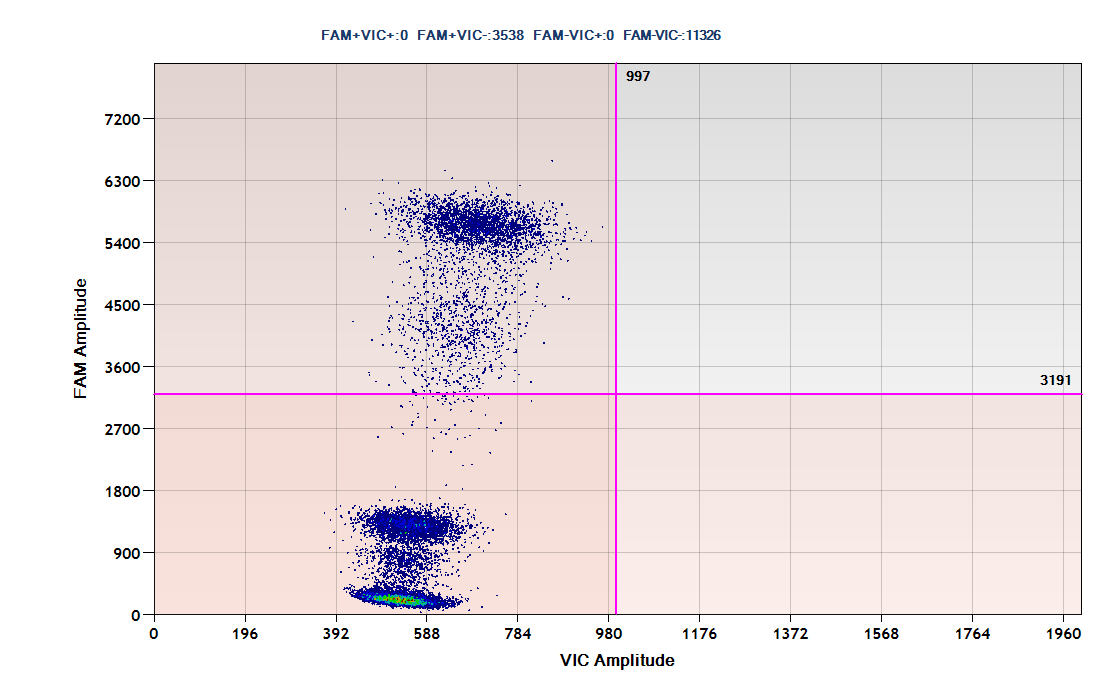


A

B

C

D

E

F


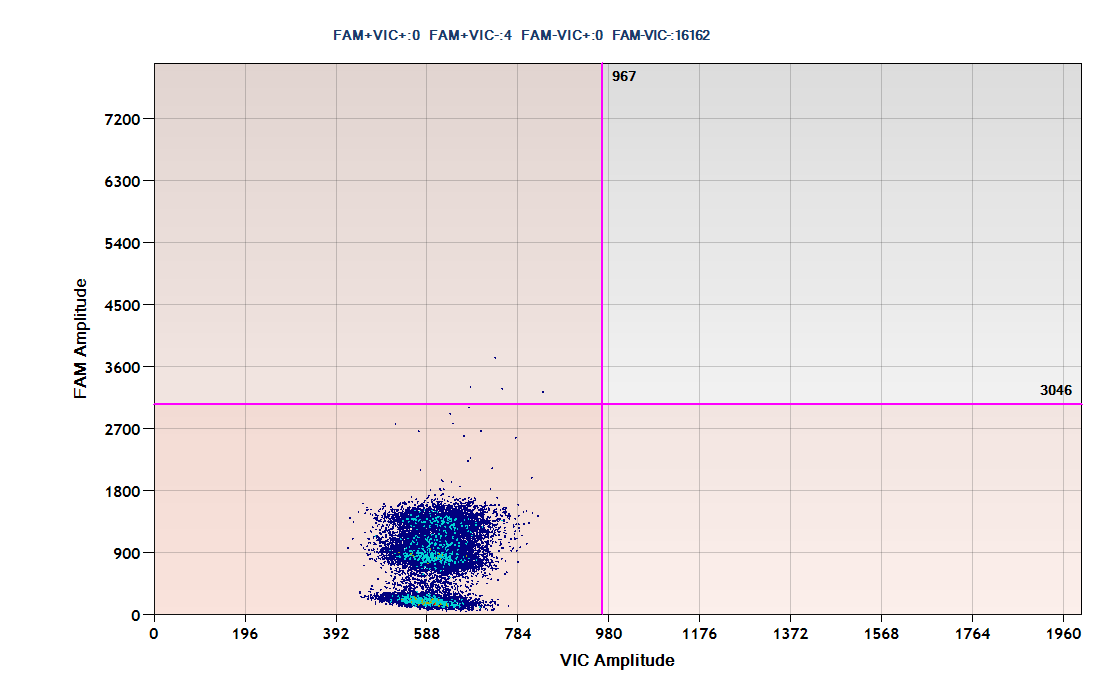

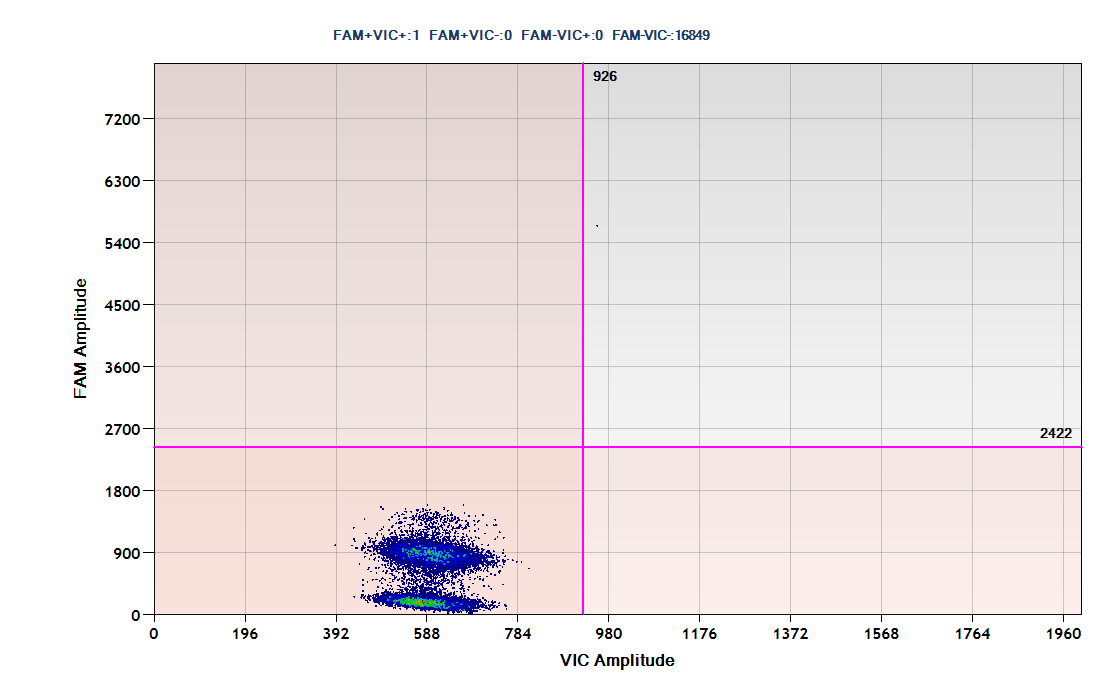


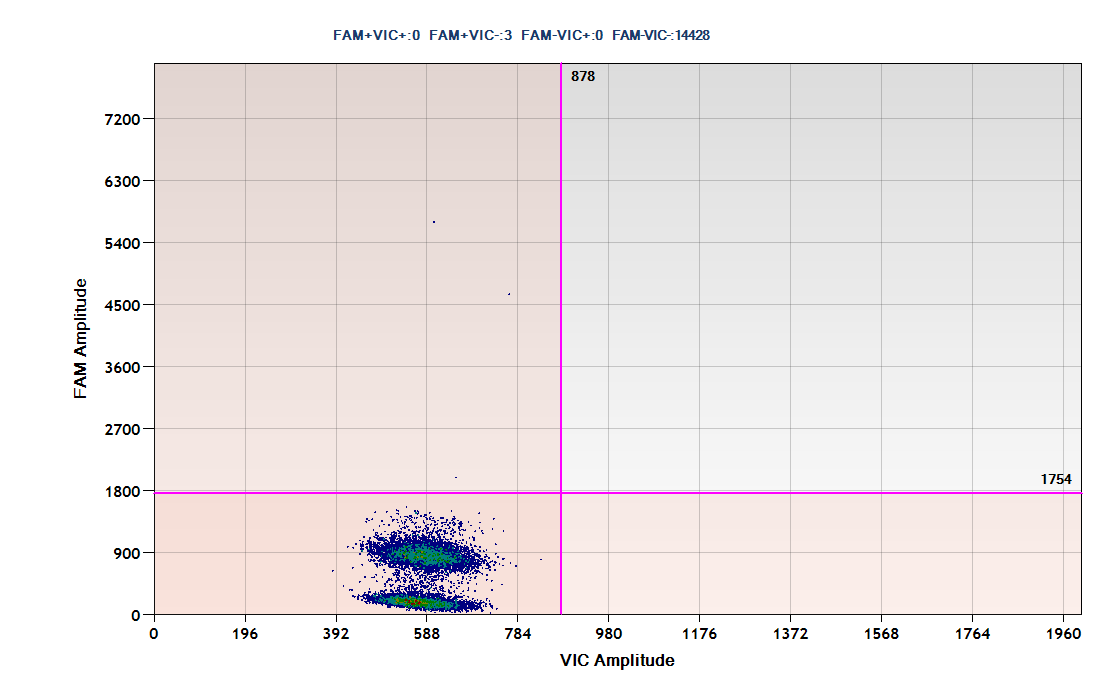

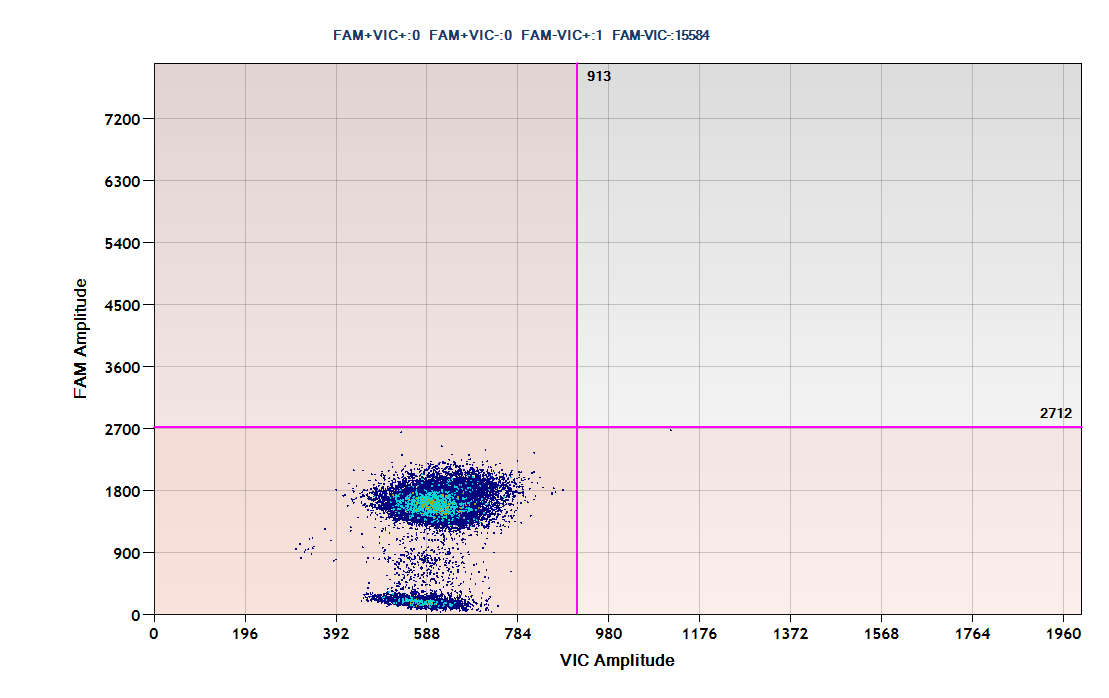


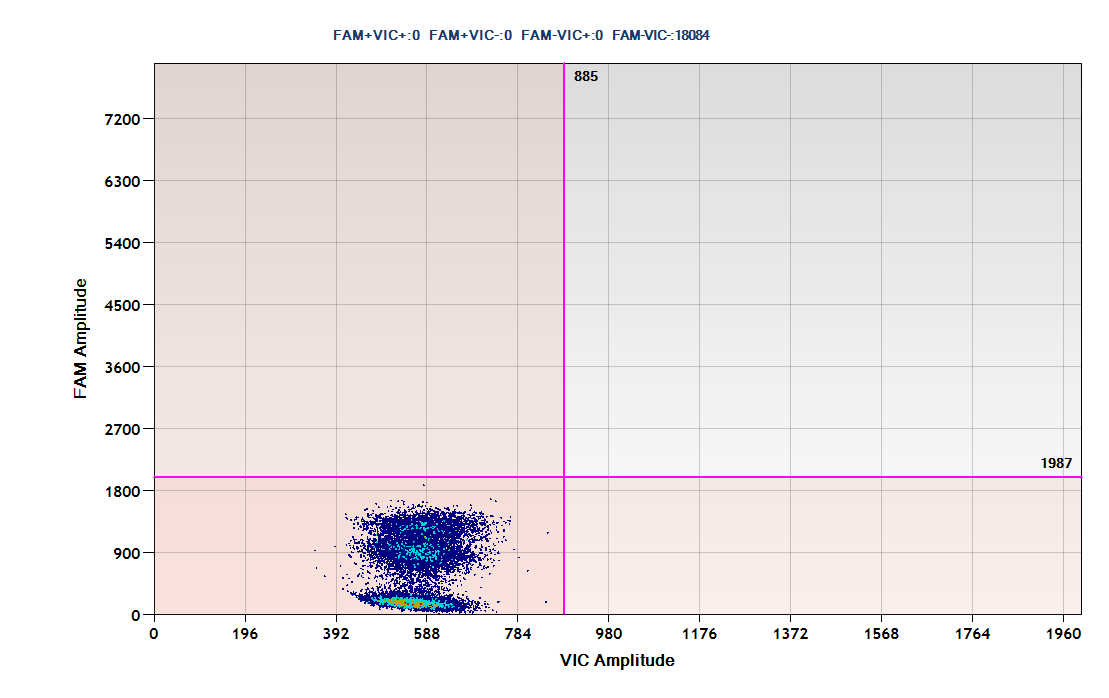


G

Figure S3. Cross reaction evaluation between G12D specific assay and each *KRAS* mutant DNA (A, RPMI-8226; B, SUN-C2B; C, NCI-H157; D, SW1573; E, A549; F, SW620; G, HCT-116).


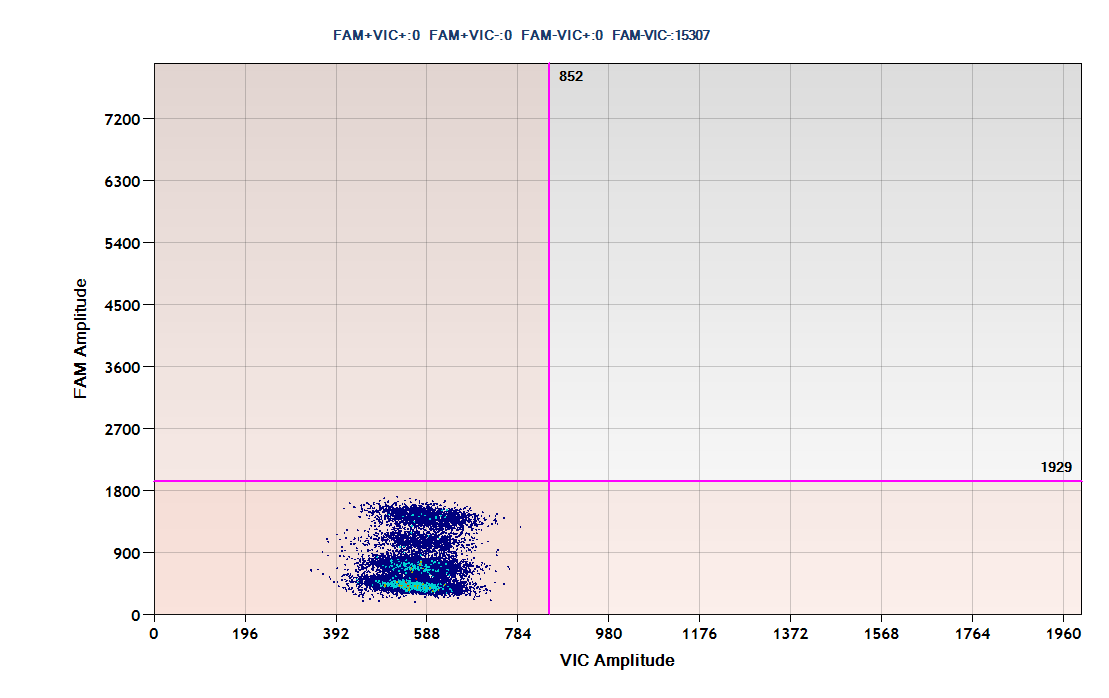

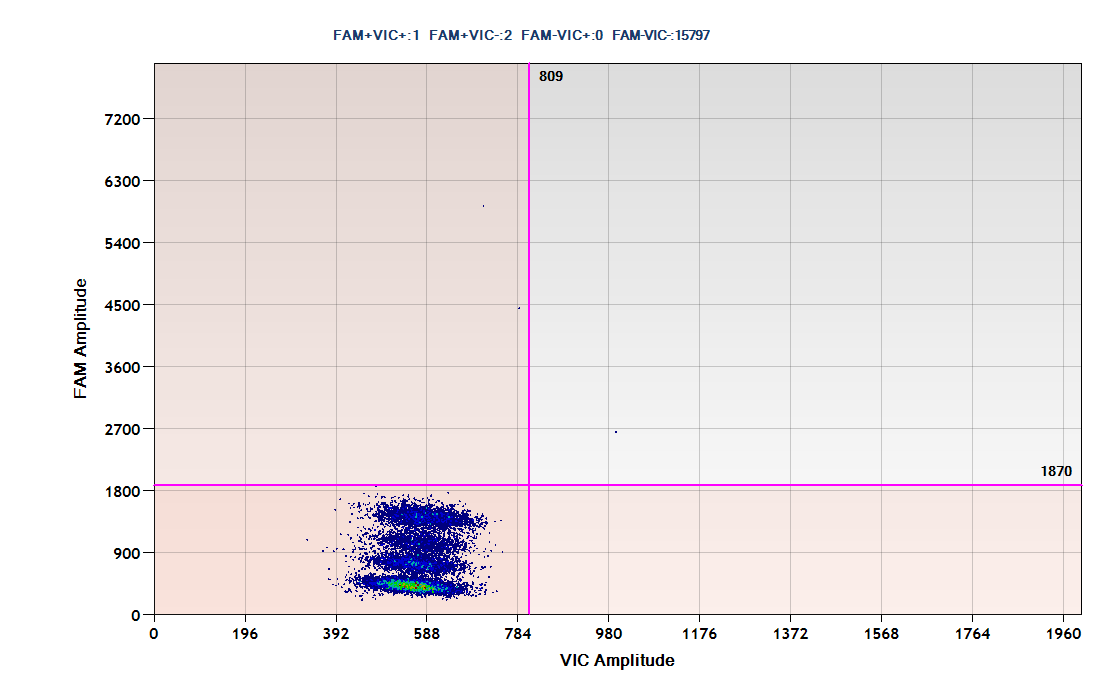

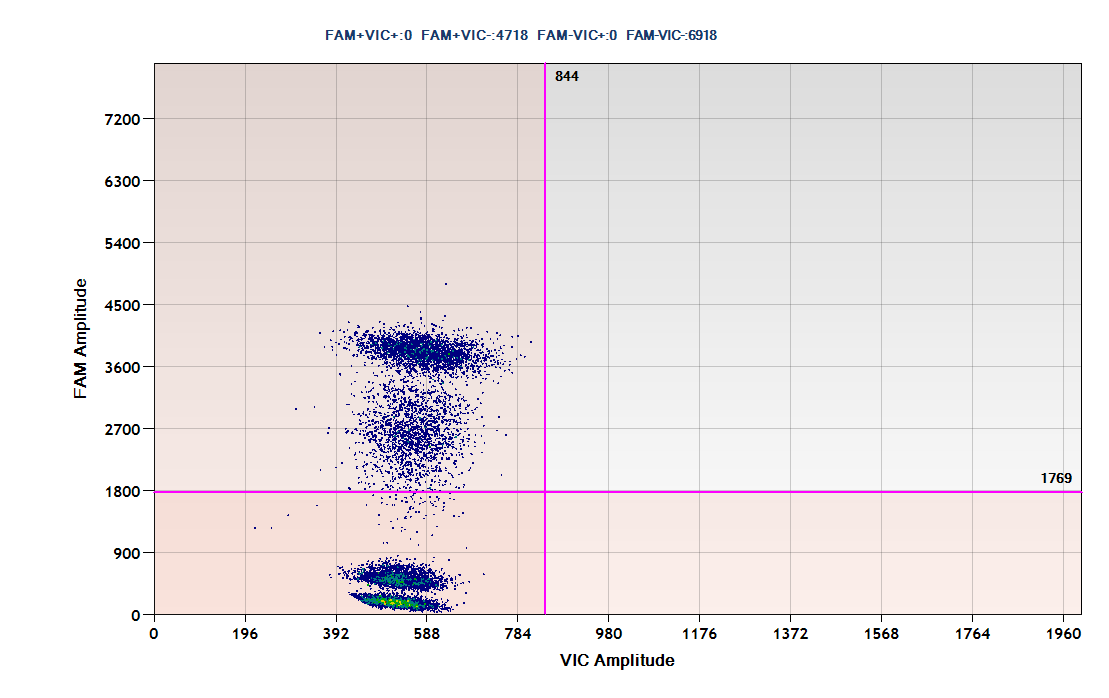

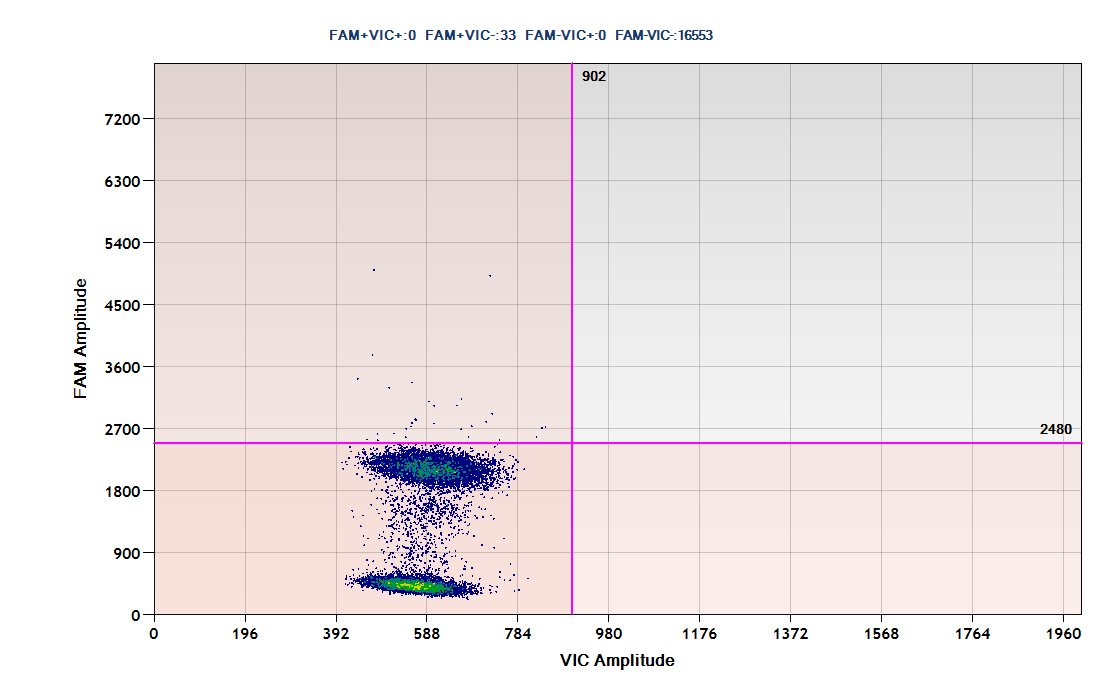


A

B

C

D

E

F


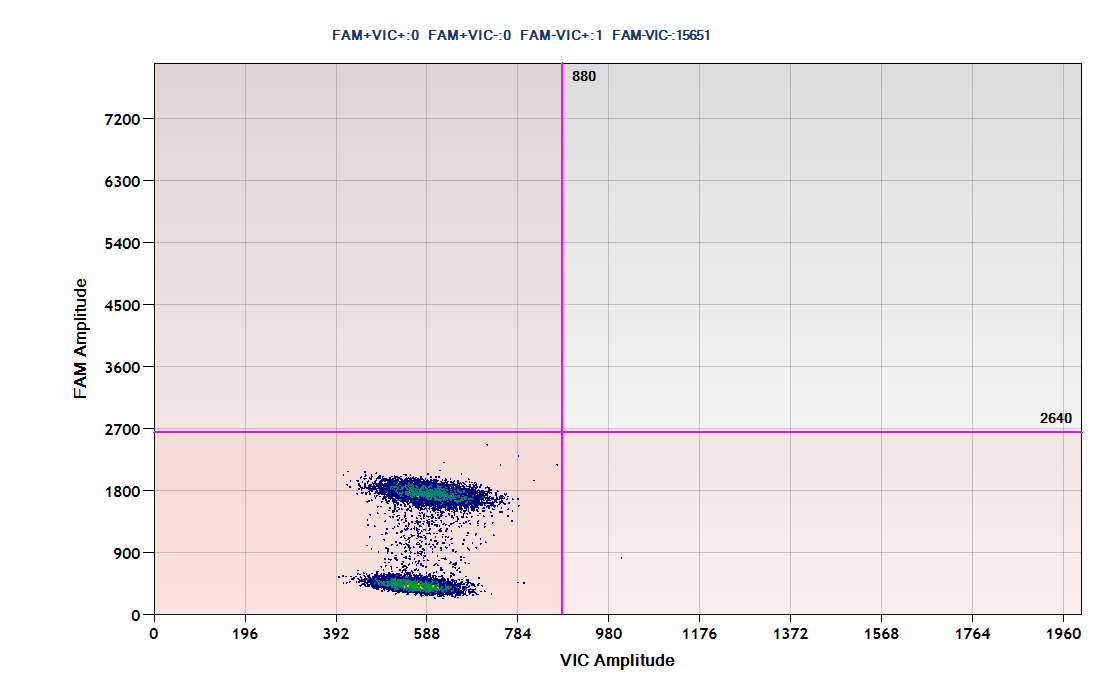

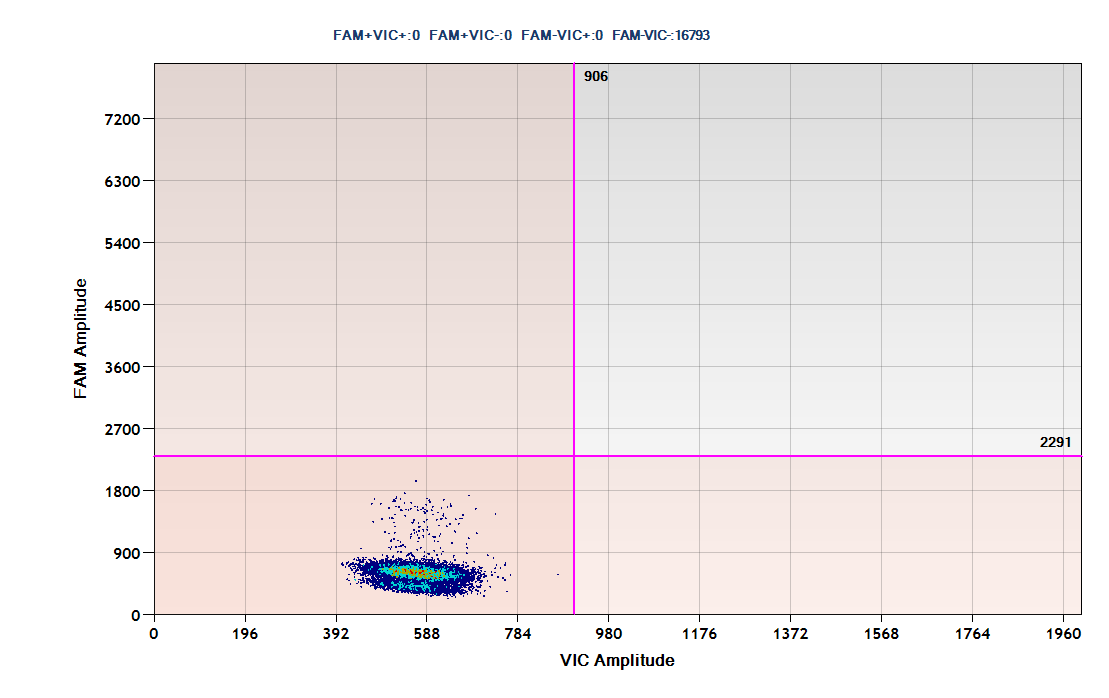


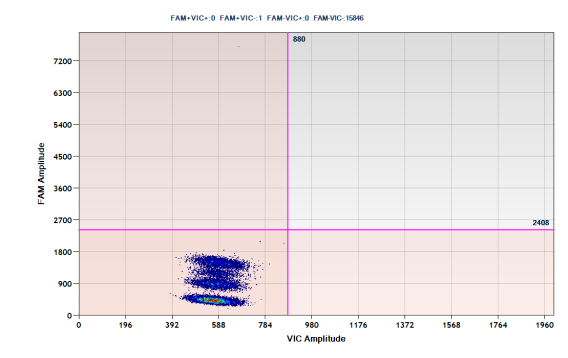


G

Figure S4. Cross reaction evaluation between G12R specific assay and each *KRAS* mutant DNA (A, RPMI-8226; B, SUN-C2B; C, NCI-H157; D, SW1573; E, A549; F, SW620; G, HCT-116).

A

B

C


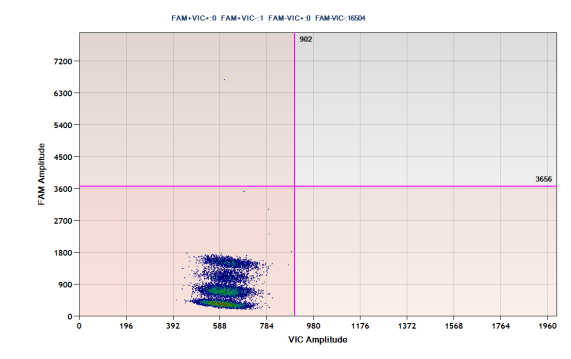

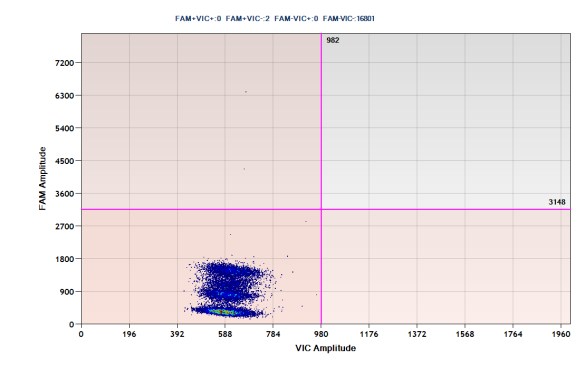


D


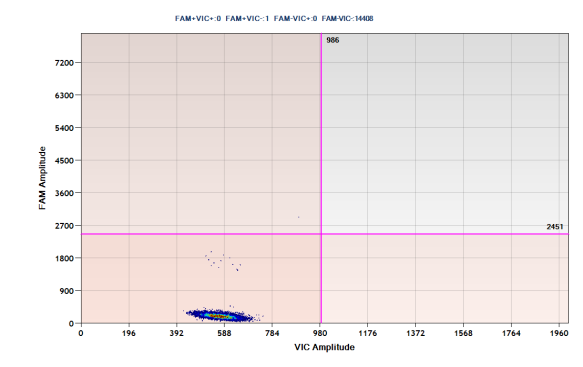

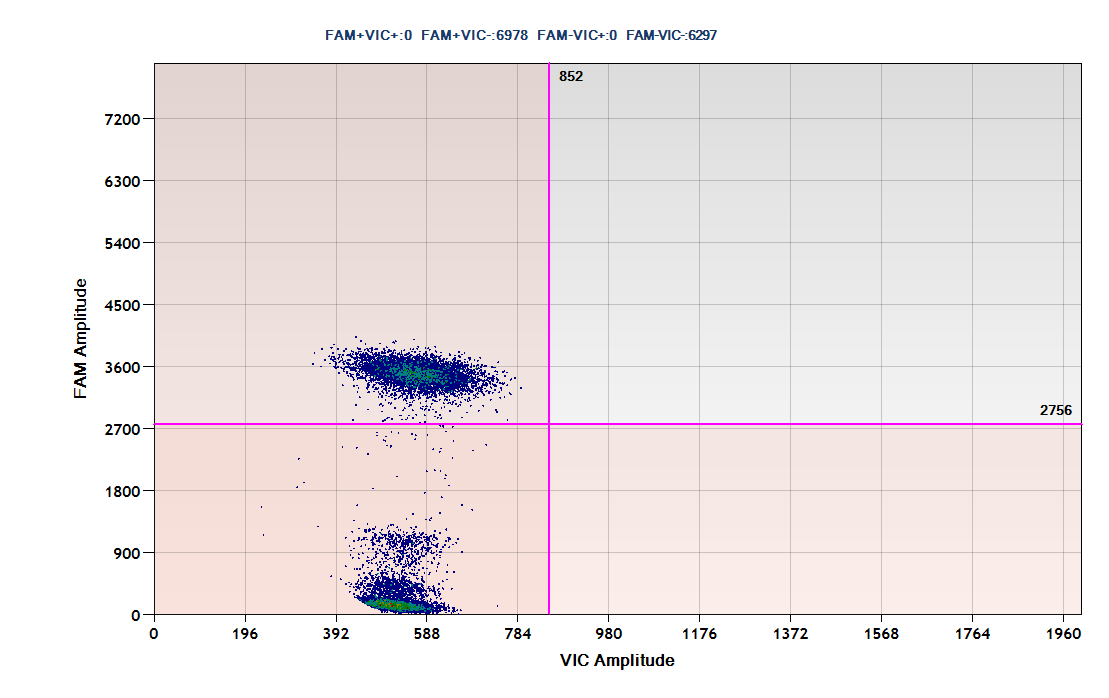


F


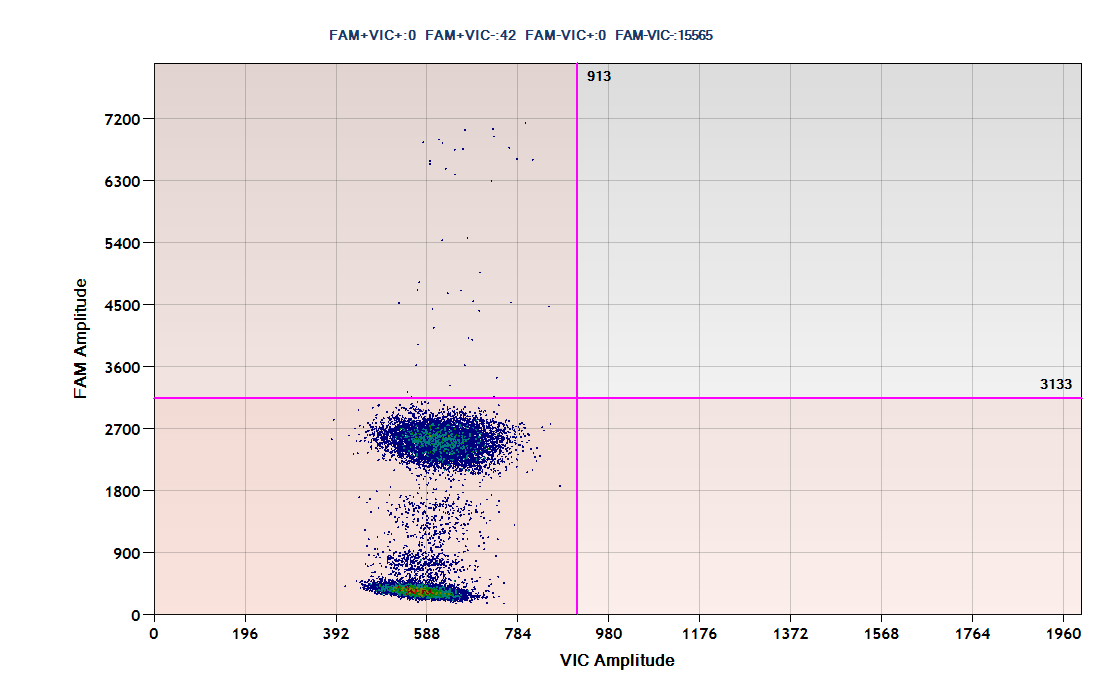

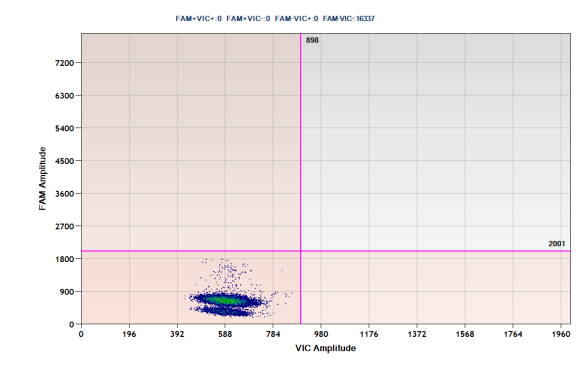


E


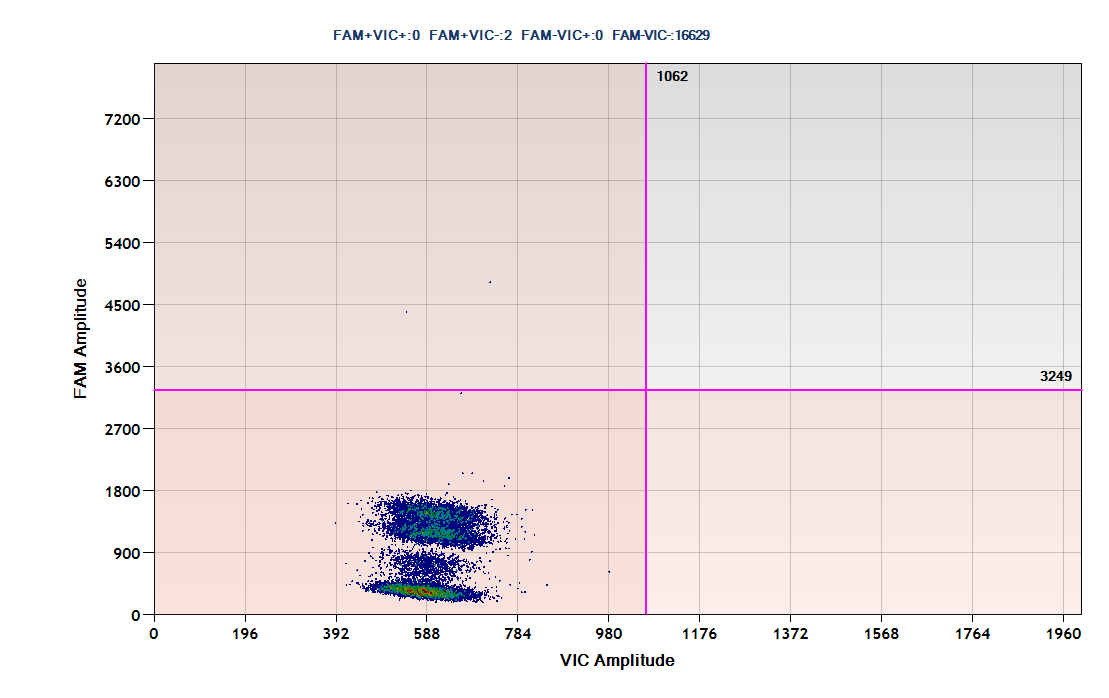


G

Figure S5. Cross reaction evaluation between G12C specific assay and each *KRAS* mutant DNA (A, RPMI-8226; B, SUN-C2B; C, NCI-H157; D, SW1573; E, A549; F, SW620; G, HCT-116).

A

B

C


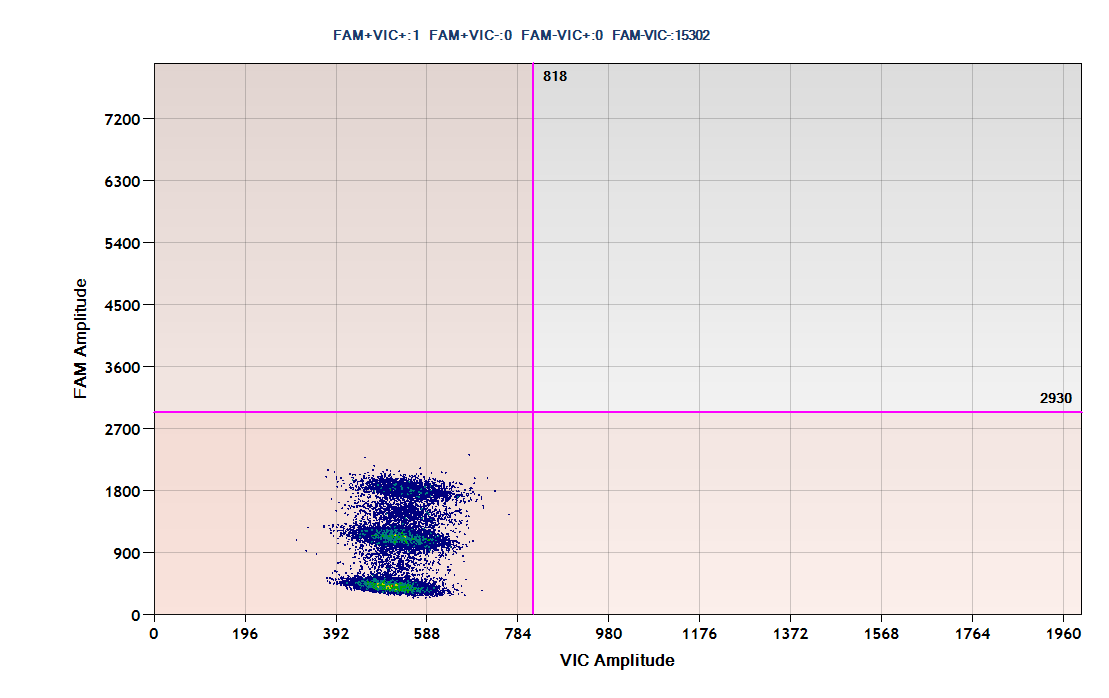

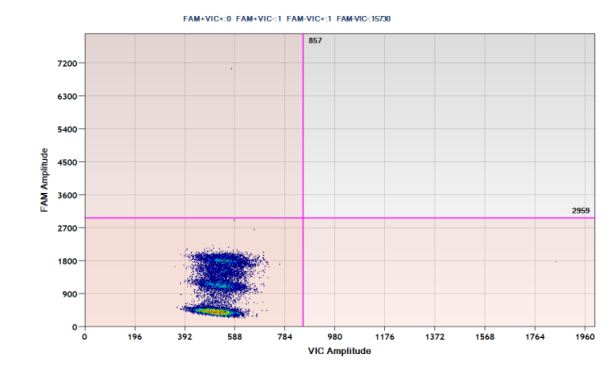


D


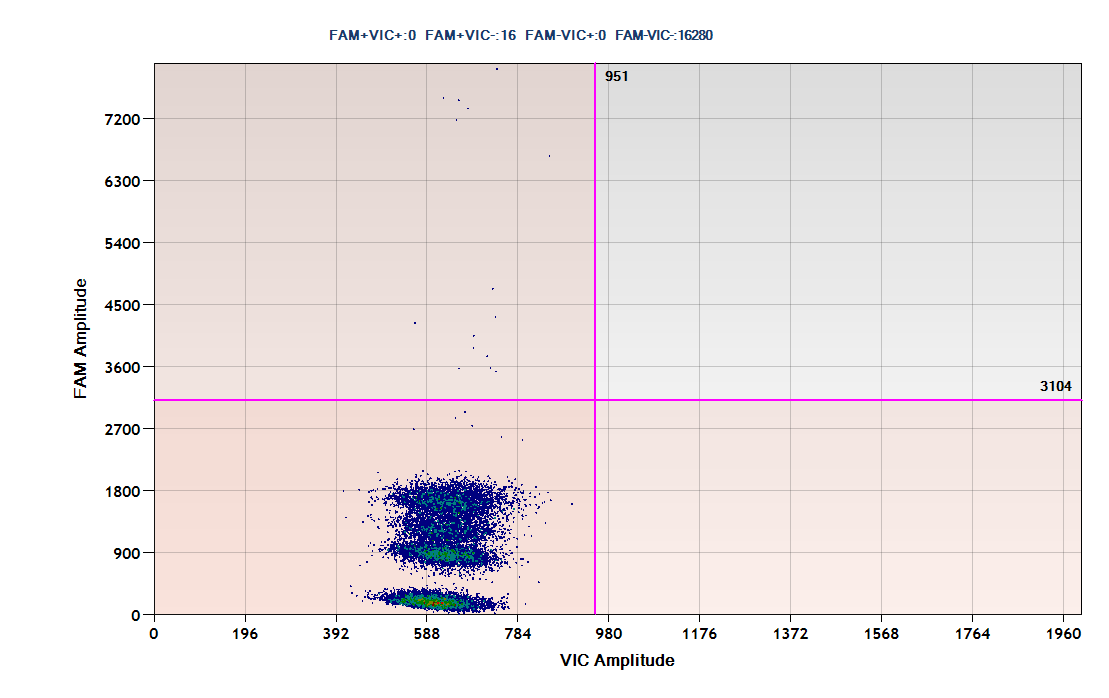

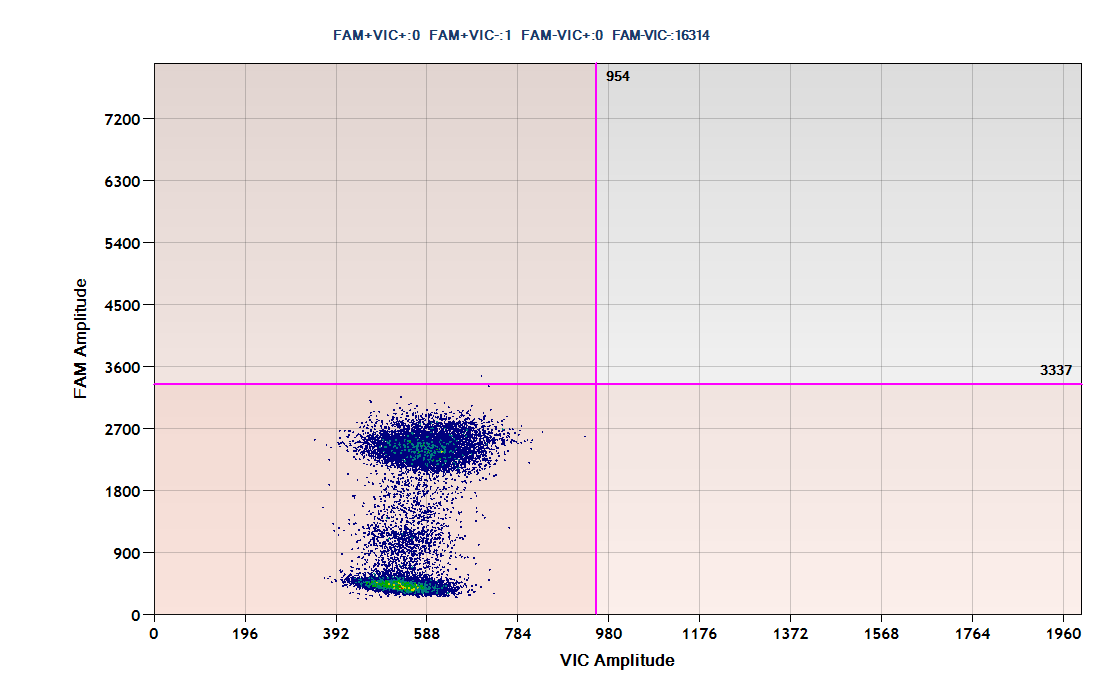


E

F


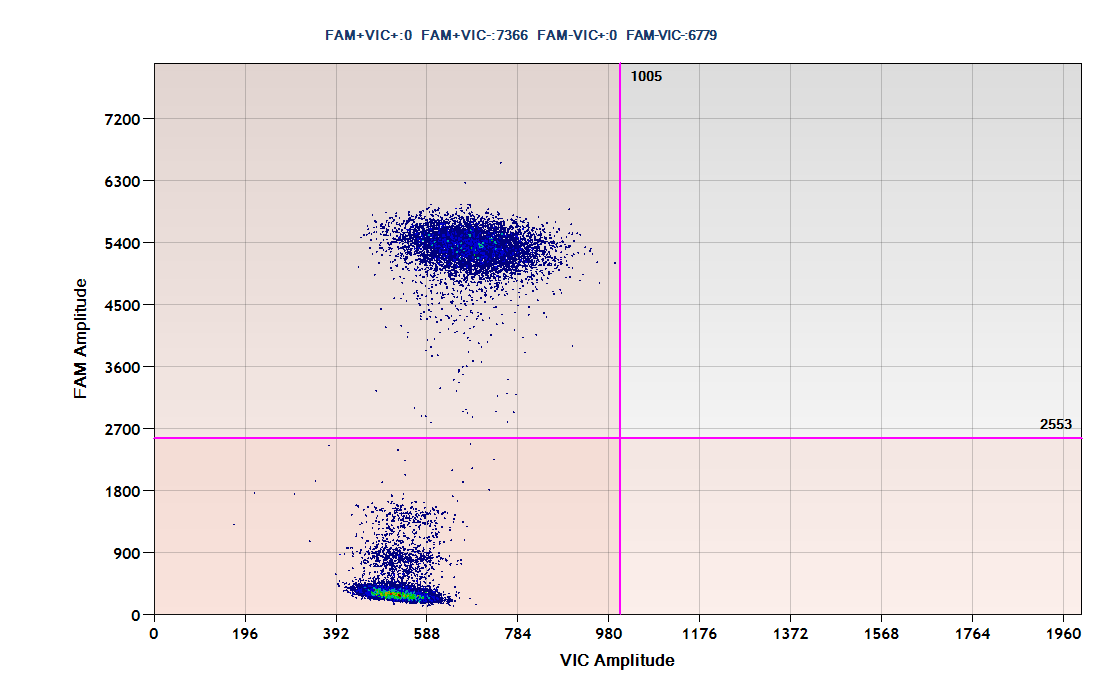

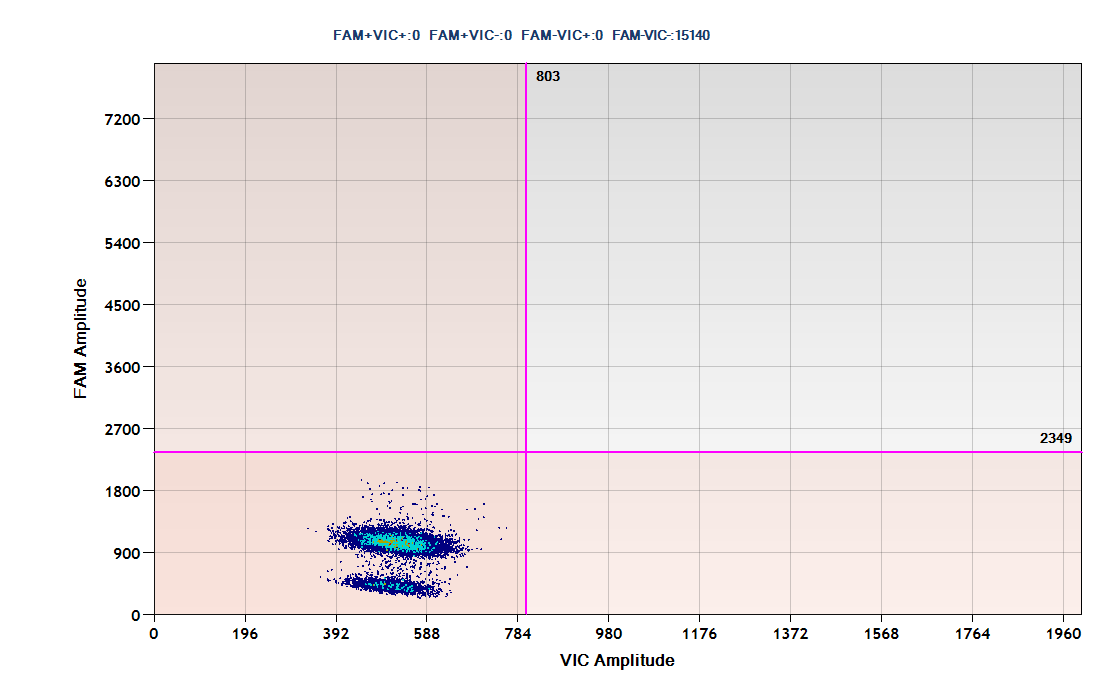


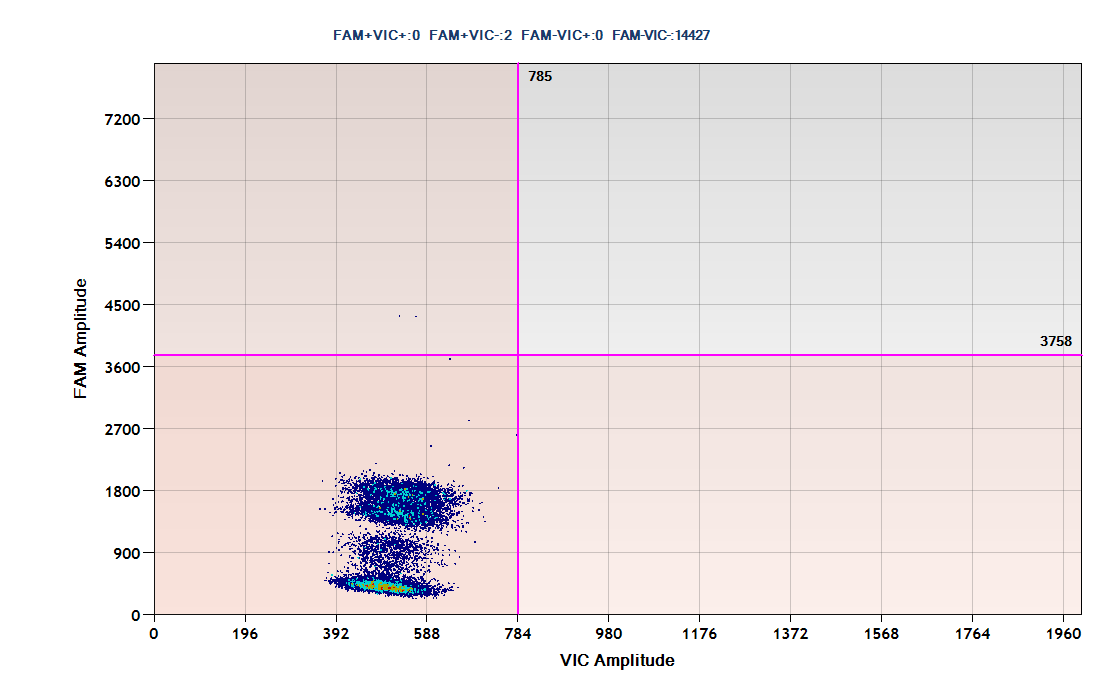


G

Figure S6. Cross reaction evaluation between G12S specific assay and each *KRAS* mutant DNA (A, RPMI-8226; B, SUN-C2B; C, NCI-H157; D, SW1573; E, A549; F, SW620; G, HCT-116).

A

B


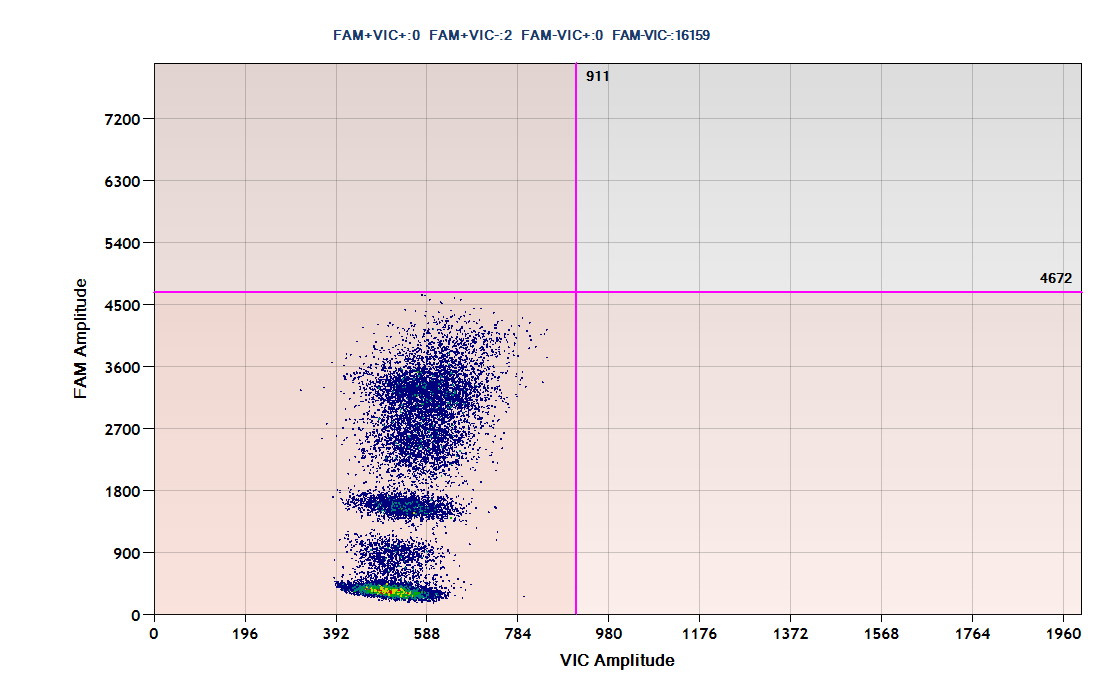

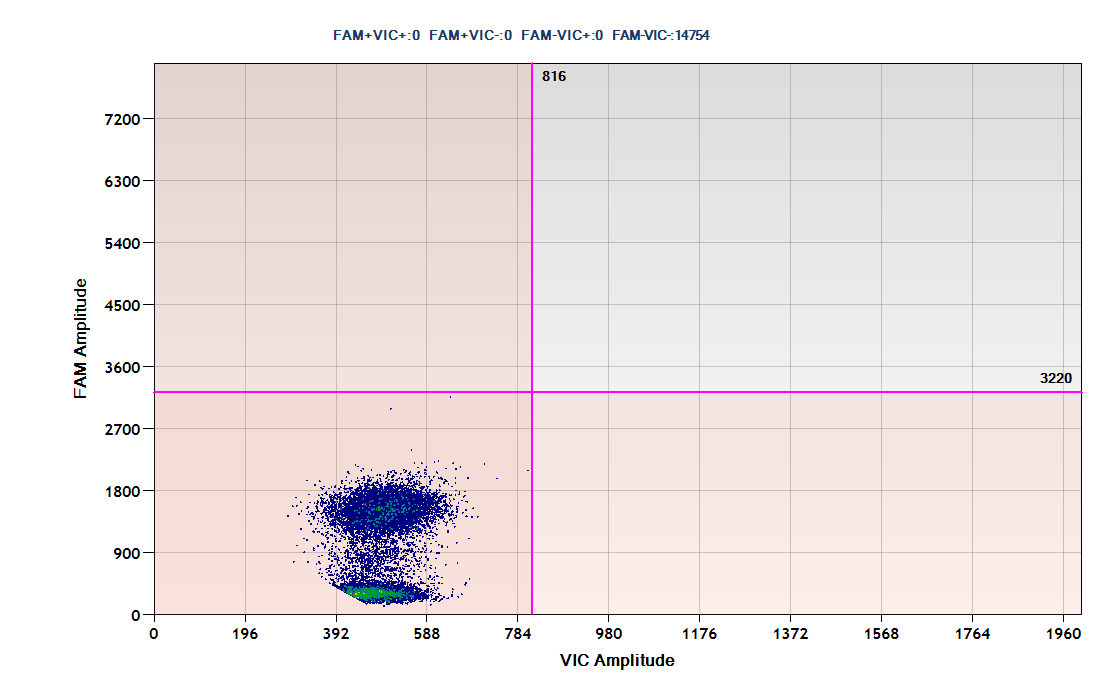


D


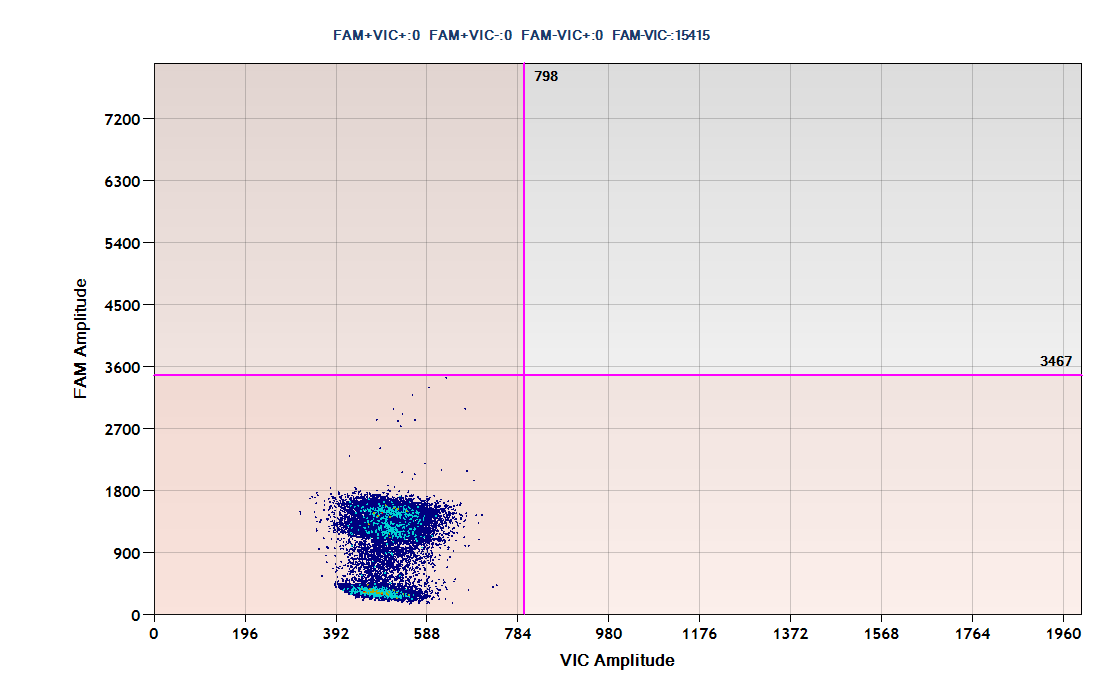

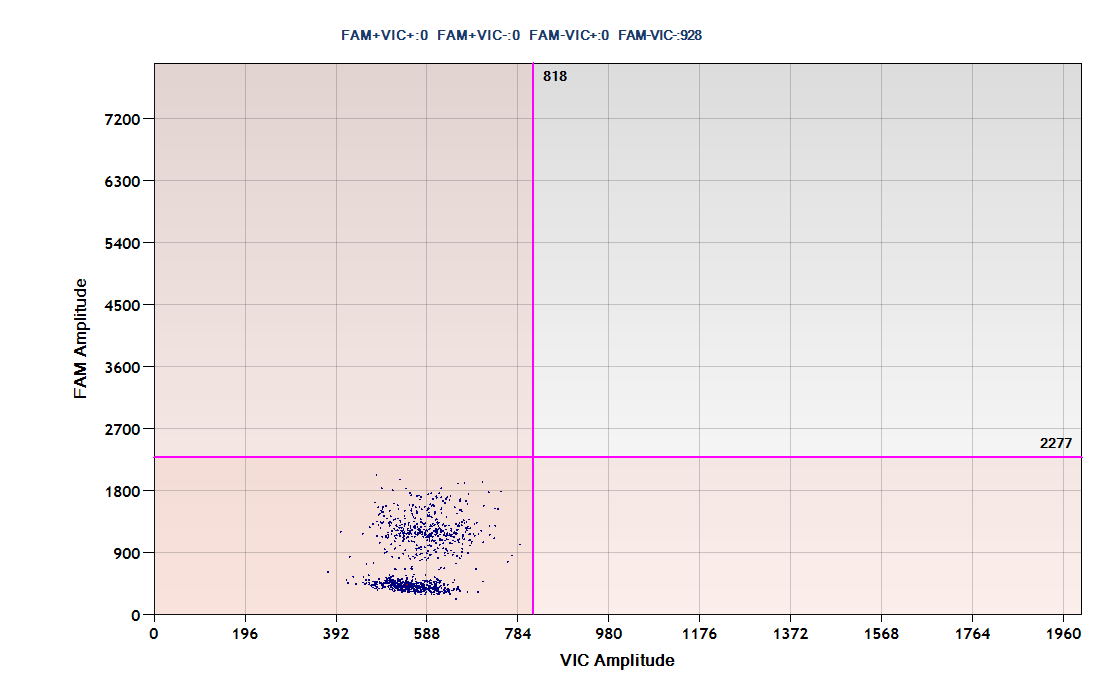


C

F

E


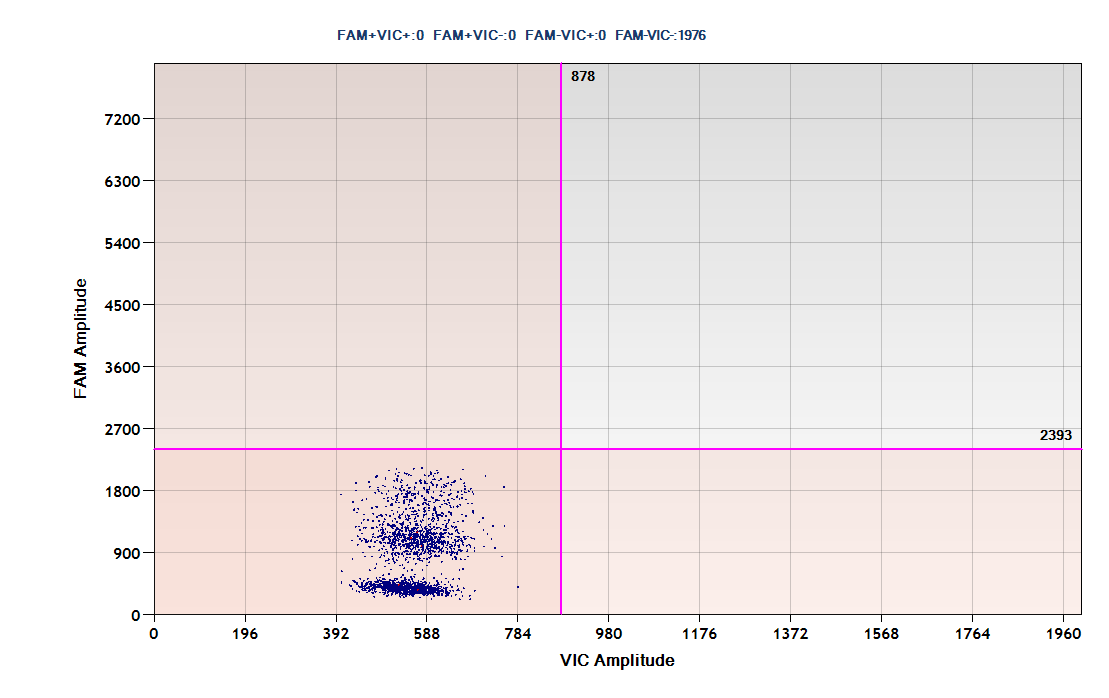

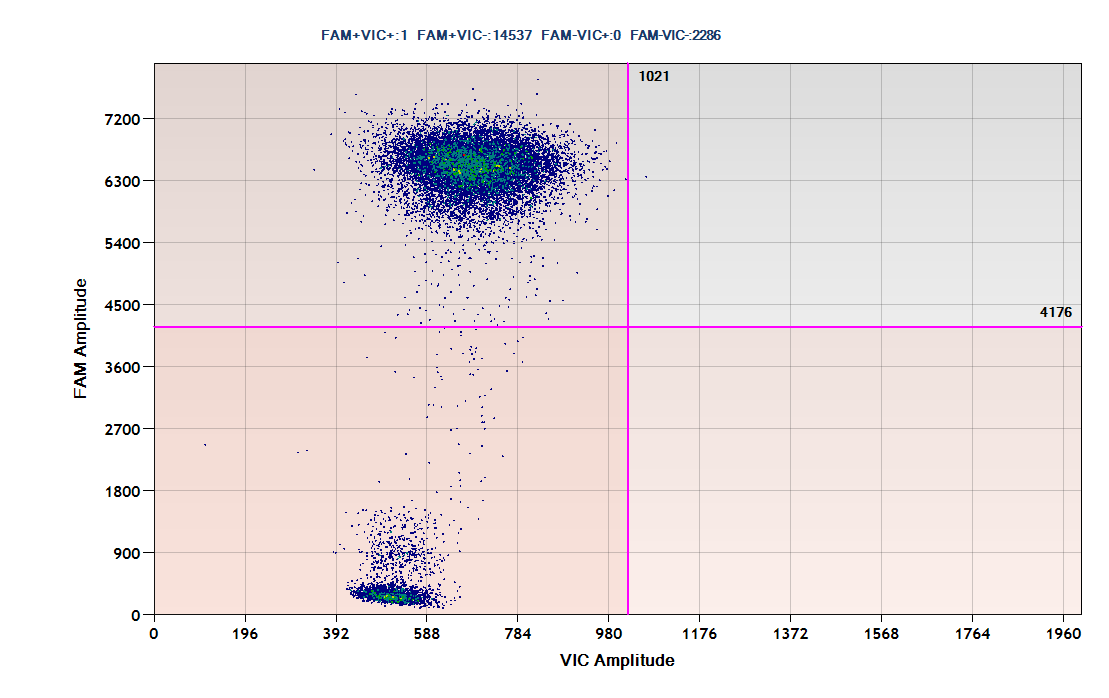


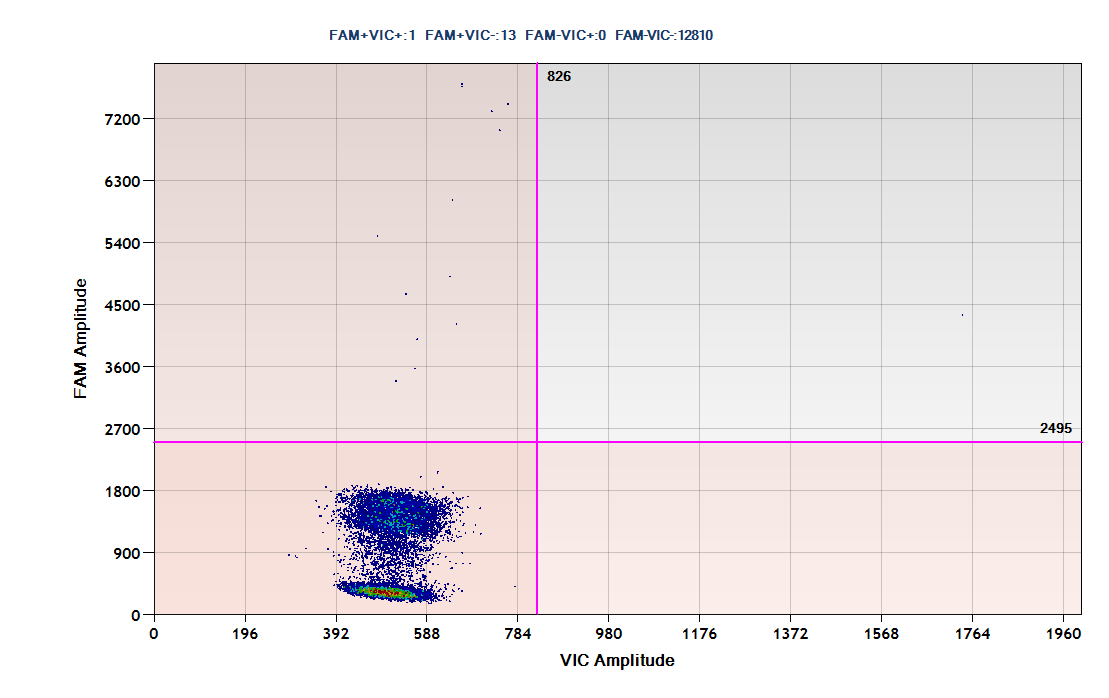


G

Figure S7. Cross reaction evaluation between G12V specific assay and each *KRAS* mutant DNA (A, RPMI-8226; B, SUN-C2B; C, NCI-H157; D, SW1573; E, A549; F, SW620; G, HCT-116).


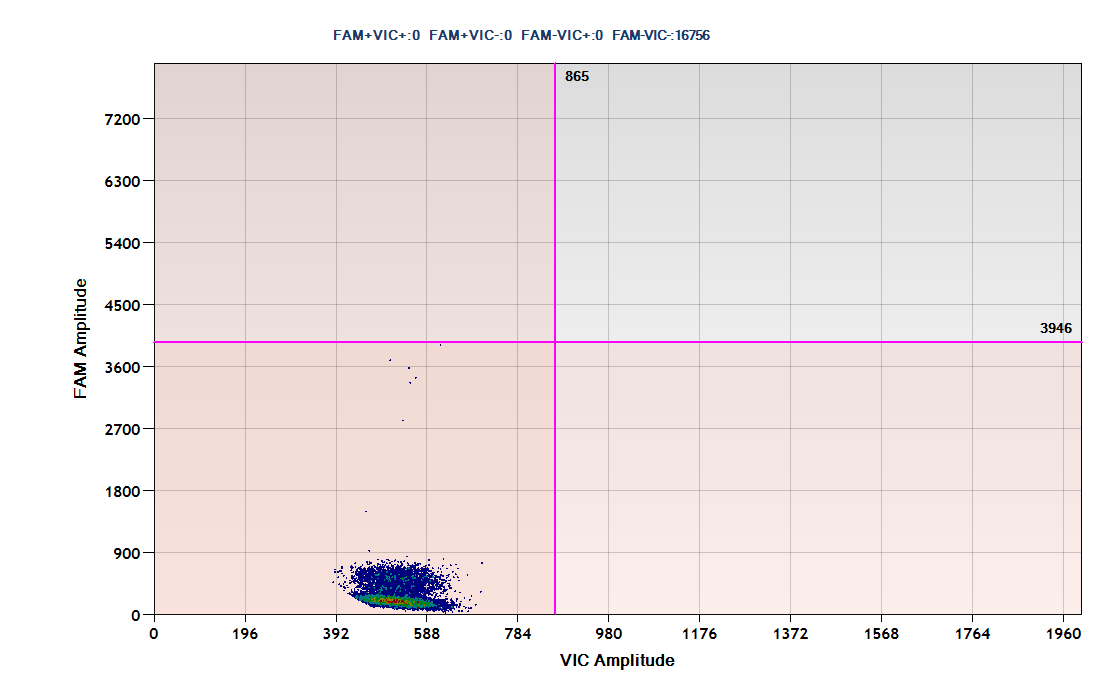

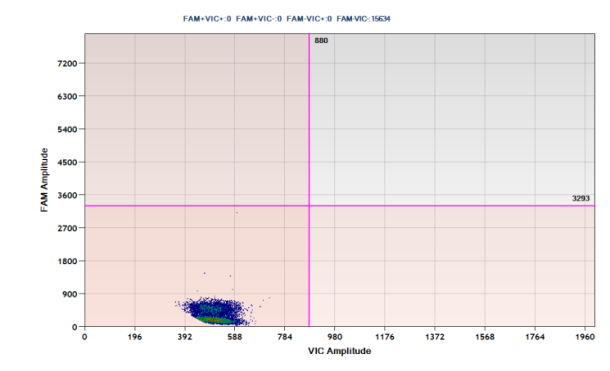


A

B

D

C


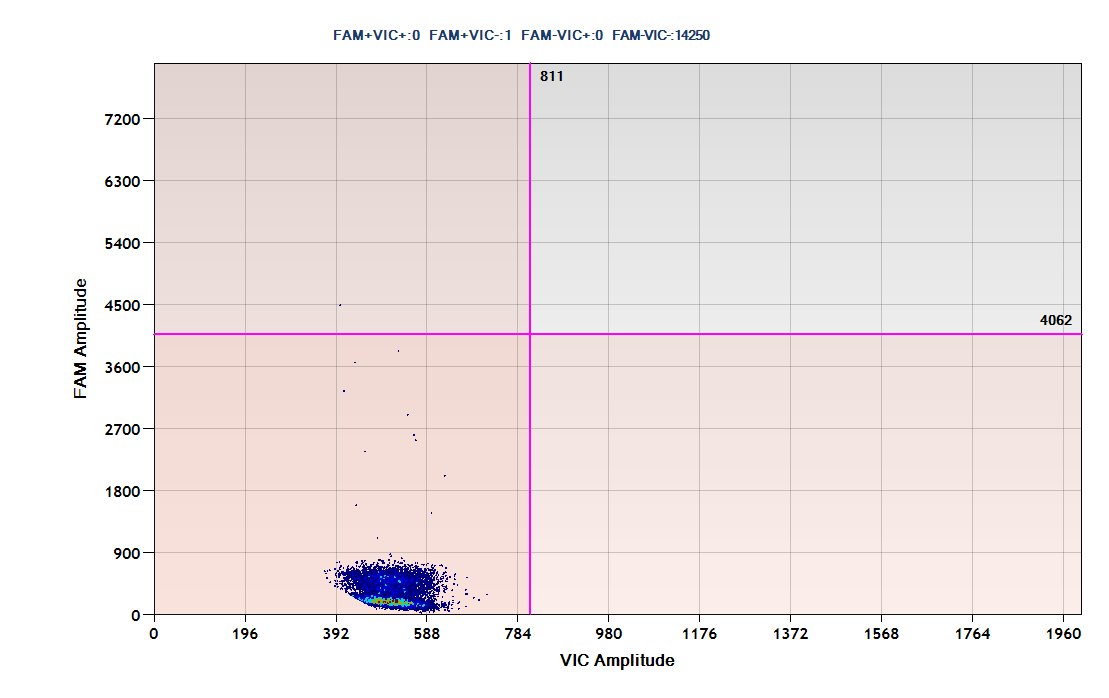

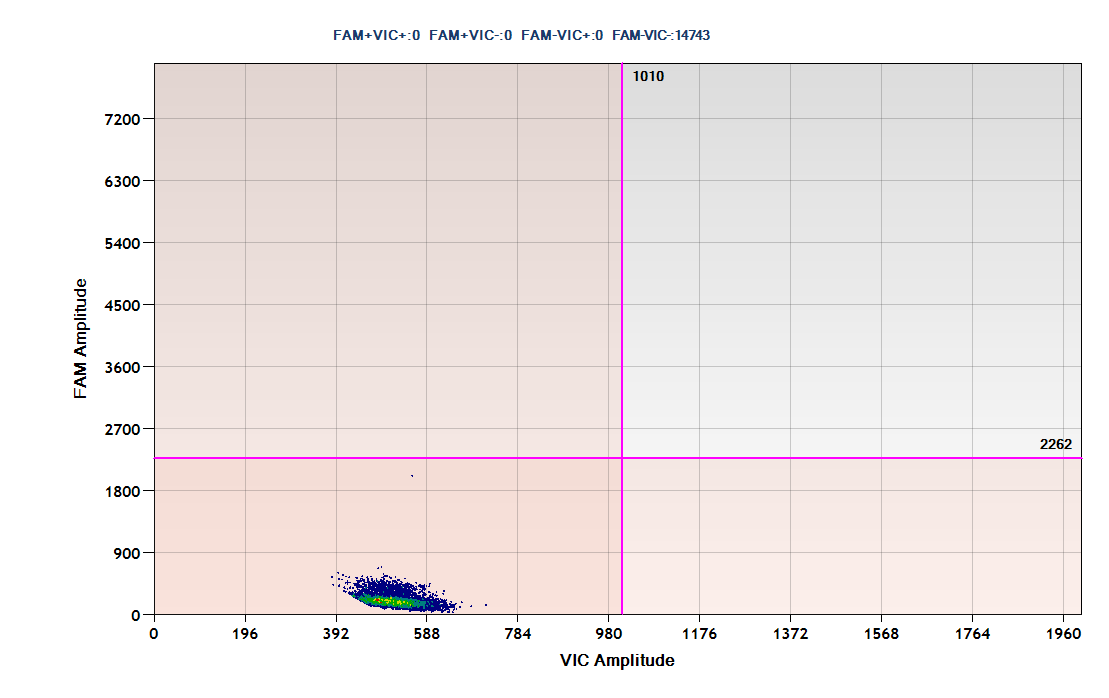


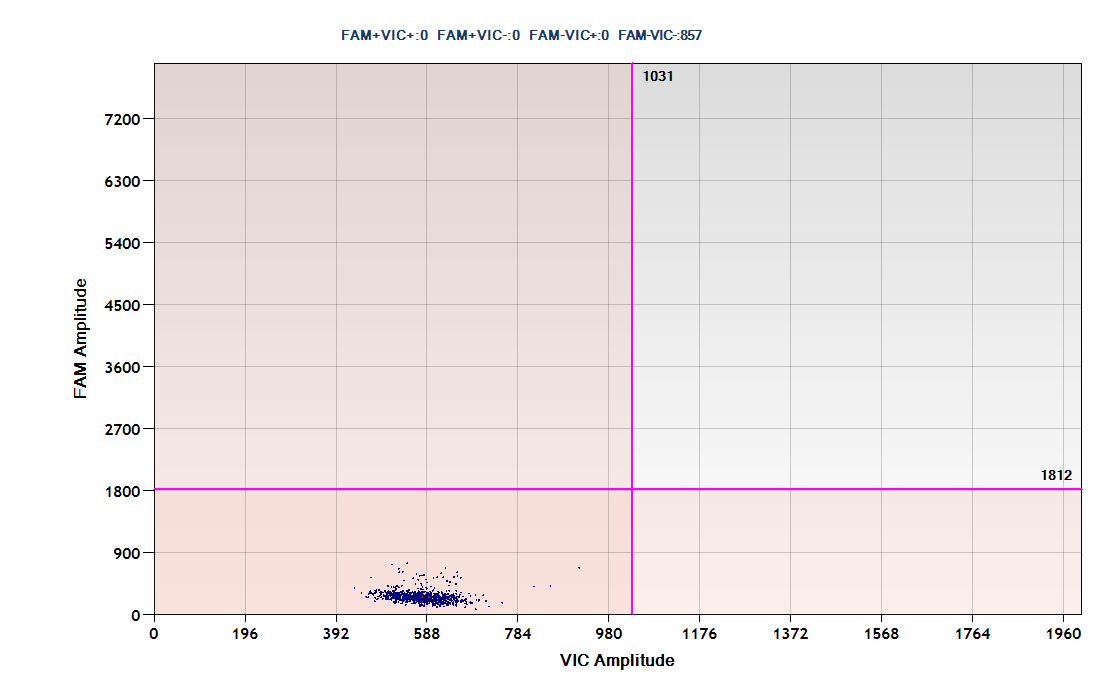

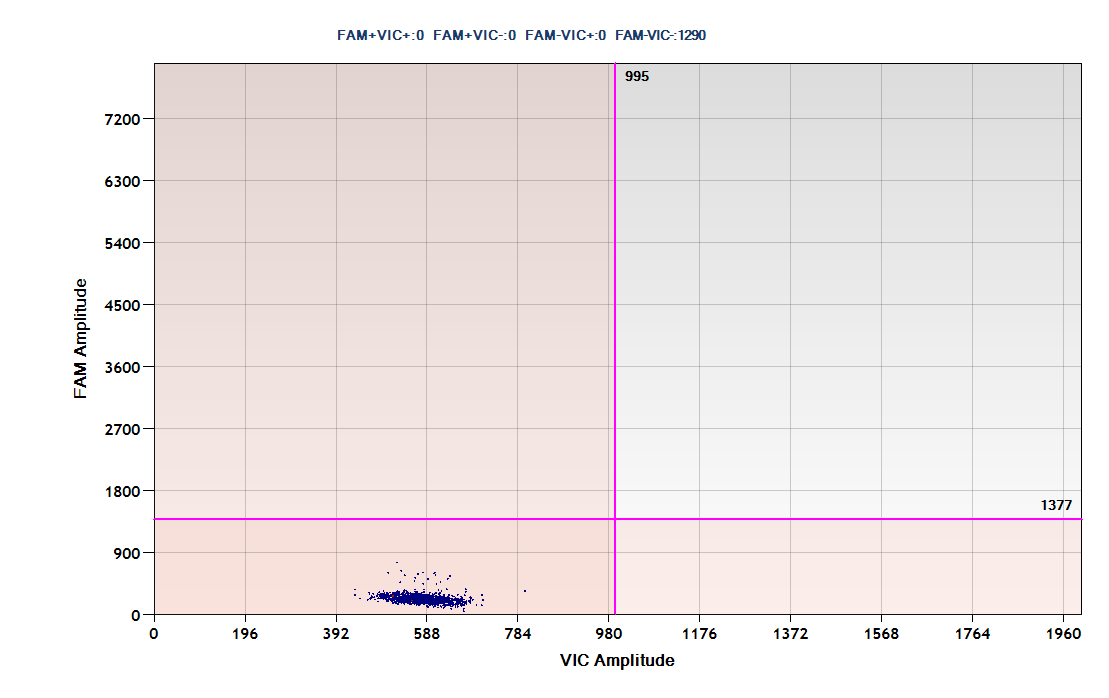


E

F


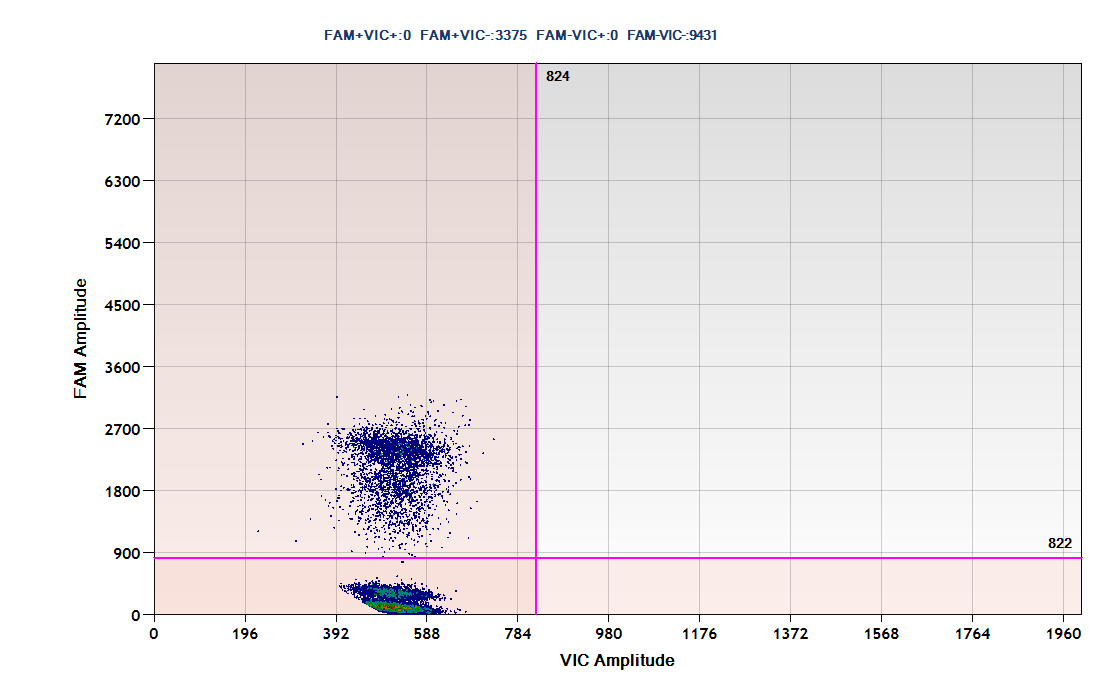


G

Figure S8. Cross reaction evaluation between G13D specific assay and each *KRAS* mutant DNA (A, RPMI-8226; B, SUN-C2B; C, NCI-H157; D, SW1573; E, A549; F, SW620; G, HCT-116).


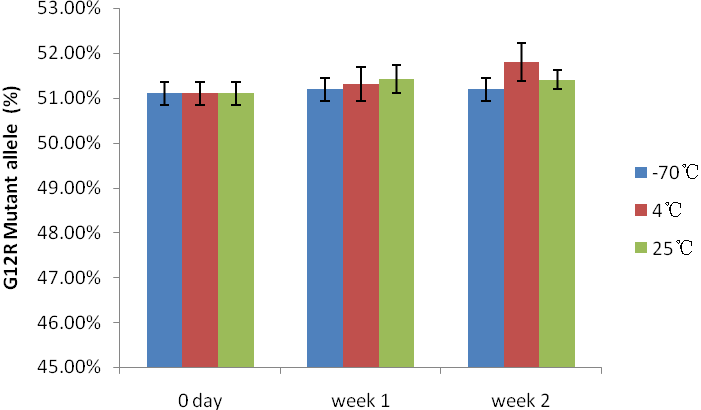


Figure S9. Short term stability data of G12R reference material determined by ddPCR
